# What is the evidence for the impact of ocean warming on subtropical and temperate corals and coral reefs? A systematic map

**DOI:** 10.1186/s13750-024-00349-y

**Published:** 2024-11-21

**Authors:** Man Lim Ho, Malgorzata Lagisz, Shinichi Nakagawa, Sarah Perkins-Kirkpatrick, Paige Sawyers, Charlotte Page, Bill Leggat, Troy Gaston, Alistair J. Hobday, Zoe Richards, Tracy Ainsworth

**Affiliations:** 1 Centre for Marine Science and Innovation, School of Biological, Earth and Environmental Sciences, University of New South Wales, Sydney, NSW Australia; 2 Evolution and Ecology Research Centre, School of Biological, Earth, Environmental Sciences, University of New South Wales, Sydney, NSW Australia; 3 Fenner School of Environment and Society, Australian National University, Canberra, Australia; 4 School of Environmental and Life Sciences, The University of Newcastle, Ourimbah, NSW Australia; 5 CSIRO Environment, Hobart, TAS Australia; 6 School of Molecular and Life Sciences, Curtin University, Perth, WA Australia

**Keywords:** Coral, Coral reefs, Scleractinian, High latitude, Marginal, Ocean warming, Climate change, DHW, MHW

## Abstract

**Background:**

Subtropical coral reefs are comparatively understudied compared to tropical coral reef ecosystems, yet also host a diverse and abundant array of marine life and provide substantial socio-economic benefits to communities. Research into the impacts of ocean warming on subtropical coral reefs has increased over the past two decades due to increase frequency and intensity of bleaching and degradation of these ecosystems. Understanding the extent of research effort and type of evidence assessing the response of subtropical corals and reefs to ocean warming provides valuable insight into global patterns in research efforts allowing critical knowledge gaps to be identified. A comprehensive understanding the impact of ocean warming on these systems will underpin our ability to predict and respond to future changes on subtropical coral reefs. Here, a systematic-map approach is used to identify recent research effort, from 2010 to 2023, and highlight patterns in the type, scale, and location of research conducted and as well as identify the availability of data and evidence reported.

**Methods:**

Primary literature was identified by searching Scopus and Science Citation Index Expanded through Web of Science Core Collection databases. The methodologies provided in a previously published systematic map protocol were applied, and 90 primary research publications were subsequently identified. Data extraction from the identified literature included bibliometric data, discipline and type of research, type of data reported and how it was recorded, and data availability.

**Findings:**

The identified literature consisted primarily of experimental (49%) and observational (39%) studies. The majority of the primary literature investigated corals in the ecoregions of Southern China (13%), Western Mediterranean (10%) and across a total of seven ecoregions grouped within Oceania (29%). Stressors reported in the literature as drivers of ocean warming reflect the standardisation of methods applied in reporting of events within the literature. Standardised metrics related to degree heating weeks (DHW) and marine heatwaves (MHW) have been reported when assessing the occurrence and severity of drivers, and are increasing in recent years, particularly in Australia. Finally, the need for increased research effort across much of the subtropics is evident, particularly for understudied regions such as the Western Indian Ocean where there are far fewer studies than other similar subtropical coral reef ecosystems.

**Conclusions:**

Climatic change, increasing ocean temperatures, and the impacts to subtropical and temperate coral reefs are of increasing concern to policy makers and researchers alike. This systematic map provides a broad overview of research topics and effort around the globe since 2010 and identifies areas where more research effort is urgently needed. Our study has identified major research clusters in Asia, Australia, the Mediterranean, and North America and gaps of research in regions such as the East Indian Oceans. Of the research conducted to date approximately one third reports on evidence related to marine protected areas and the vast majority of evidence is from close/territorial sea locations, providing important knowledge base for management of these areas. Of the 17 studies reporting on specific extreme events (rather than experimental studies which is the majority of evidence identified here) 13 have been published since 2019, with the majority reporting on events occurring in 2019/20 indicating a trend of increasing evidence in recent years (a total of 7 studies from 2010 to 2013, compared to over 10 studies published annually since 2019 up to mid-2023).

**Supplementary Information:**

The online version contains supplementary material available at 10.1186/s13750-024-00349-y.

## Background

Coral reef ecosystems, identified as reef systems dominated by corals, are some of the most valuable ecosystems in the world, where unique marine biodiversity provides a range of socio-economic benefits to communities [1]. However, coral reefs are declining globally [2, 3], threatening a loss of marine biodiversity and local species extinctions [4]. Coral species include vast populations solitary corals in polar and temperate regions, to the spectacular reef structures of the tropics. Tropical coral reefs been the focus of coral research and long-term studies have shown climate-related declines across reefs and species in these regions [5–10].

Coral bleaching occurs in response to increased water temperatures (1–2 °C above average) and in combination with high irradiance results in the breakdown of the coral host symbiosis with the dinoflagellate Symbiodinacea, exposing the coral skeleton and the coral becoming visibly white [11]. The breakdown of the coral photoendosymbiosis is a generalised stress response of the coral and other stressors such as pollution, ocean acidification also results in coral bleaching [12–14]. Since 2010, bleaching events have also been recorded on subtropical coral reef ecosystems including Hong Kong [15, 16], Japan [17], and on Australia’s Lord Howe Islands [18]. Subtropical coral reefs are unique and important habitats for a range of both subtropical and tropical marine organisms, including a number of endemic taxa [19–21]. Several hypotheses have emerged on the importance of these systems including the capability of coral reefs in subtropical regions to act as refugia for tropical species [22–24], and poleward expansion of tropical species [25, 26]. Other studies point to the higher risk of endemic species from subtropical systems due to limited connectivity with source populations and thermal susceptibility of species [27, 28]. As such, uncertainty remains in predicting survival, diversity, and coverage of subtropical coral species as ocean warming increasingly impacts many of these regions globally.

Understanding the capacity for subtropical coral reef ecosystems to preserve and continue to provide valuable ecosystem goods and services is vital for management of these systems. For example, the capacity for the regions to act as refugia depends largely on the surrounding oceanographic conditions. For instance, upwelling can protect thermally sensitive coral genera on the forereef of the subtropical atoll by decreasing water temperatures during extreme events and supporting coral survival [22, 24]. However, the cool, upwelled water will only provide refuge if there is a synchrony between the timing of ocean warming and upwelling [24]. Some studies have shown that under ocean warming, herbivorous marine organisms and corals from tropical regions are expanding into subtropical and temperate regions, and these expansions have the potential to alter the community structure of subtropical regions [25, 29]. A tropical poleward expansion of species in some regions could pose a threat to the endemic subtropical and temperate coral communities [30]. Endemic taxa may therefore face decline as the result of competition with tropical species [25]. The breadth of threats to corals and coral reefs across the subtropics, and the potential for vastly different outcomes of ocean warming in different regions highlights the challenges for managing subtropical coral reefs. Understanding available evidence highlights patterns in research findings, knowledge gaps, and identifies the type of evidence that is available across different ecosystems. Systematic literature assessment, including systematic maps, are becoming increasingly important step in understanding and developing management strategies particularly in complex systems [31].

Here, we provide a systematic map of the distribution and abundance of recent scientific evidence in relation to ocean warming impacts on subtropical corals and coral reefs [31]. We define recent scientific evidence as peer reviewed research publications within the period of 2010- 2023, with the insights provided here aimed to provide review of evidence from events occurring in the past 2 decades, identify knowledge gaps, and aid in directing future research efforts. We collate and classify the identified research evidence (2010–2023) identifying factors such as the location of the study, year of publication, study types, and the reported metrics of identifying ocean warming events. Regarding location, the Marine Ecoregions of the World (MEOW) [32] introduced a hierarchical biogeoregionalisation of coastal and shelf areas based on biogeographic assessments, ecoregional assessments, government-derived or supported systems and input from different researchers and assessments [32]. Ecoregions are defined as areas of relatively homogeneous species composition and clearly distinct from adjacent systems nested within Provinces, where provinces are large areas with presence of distinct biotas, that hold some level of endemism [32]. Using MEOW as a standardised reference for location enables a systematic approach for recording locational data in marine conservation planning and research. The most widely applied methods of recording extreme temperature events on coral reefs include degree heating weeks (DHW) [33–35] and marine heatwaves (MHW) [36]. Degree heating weeks (DHWs) is a measure of estimated accumulated thermal stress experienced by corals calculated by adding days where temperatures exceed the summertime maximum by at least 1 degree Celsius over a 12-week period [33–35]. A MHW is defined as a thermal event where SST exceeds the 90th percentile of the local climatological temperature for at least 5 consecutive days [36]. We also record which research disciplines, drivers of change, and methods of recording severity of coral responses to ocean warming that have been reported within the identified evidence. We also record information provided on the reef (location depth) and study (species). As such we provide a systematic map of the type, distribution, and abundance, of scientific evidence in relation to recent ocean warming impacts on subtropical corals and coral reefs [31].

## Objective of the review

The following questions outlined in the published protocol [37] are addressed in this systematic map:

What is the evidence, within scientific literature since 2010, on the impact of ocean warming to subtropical and temperate corals and coral reefs?

How have sea surface temperature (SST) data been recorded in subtropical and temperate coral reef locations?

Are there studies using MHW (Marine heatwaves) [36] and/or DHW (degree heating weeks) [33–35] as preferred metrics when evaluating climate events?

How are the levels of mortality and bleaching defined in the research locations?

Where, and how, have coral biological response and coral reef ecological impacts been recorded in subtropical and temperate coral reef locations?

The questions listed above concern the metrices of data collection and data categorisation of existing literature as well as the distribution of researchers around the world working on the topic. Through answering these questions, trends in the use of different temperature data, reporting metrices and publication timeline can be identified, as well as providing a summary of recent publications.

We utilised the PECO (Population, Exposure, Comparator and Outcomes) framework for specifying the scope of this systematic map:

- Population: Subtropical and Temperate Coral Systems and Coral Reefs. Coral systems are defined in this systematic map as corals belonging to the class Octocorallia or Hexacorallia.
- Exposure: Ocean Warming
- Comparator: Effects before and after recorded event/exposure. Control/exposure experimental studies are included.
- Outcomes: Mortality and bleaching, habitat changes, threats, acclimatisation of subtropical and temperate coral systems.

## Methods

### Protocol

We followed the Collaboration for Environmental Evidence Guidelines and Standards for Evidence Synthesis in Environmental Management [38] and conformed to the reporting standards for systematic evidence synthesis (ROSES) [39]. The systematic map is based on a previously published protocol registered in PROCEED, the global database of prospectively registered evidence reviews and syntheses in the environmental sector [37]. The ROSES form for this systematic map is provided with the submission of this manuscript as Additional File 1.

### Deviation from the protocol

“Comparators” in the PECO framework has been expanded as follows:

- Comparator: Effects before and after recorded event/exposure. Control/exposure experimental studies are included.

The following deviation was made in eligibility criteria for inclusion of this systematic map:

Studies including subtropical coral systems and any other component, including tropical, polar, or other organisms such as fishes, kelp, etc., were included in this systematic map, but only the subtropical coral systems data are extracted.

Corals here are defined as marine organisms belonging to the class Octocorallia and Hexacorallia. “Coral reefs” is used here to describe reefs with reef building corals.

If a study describes the impact of ocean warming on marine organisms living on a subtropical/temperate coral reef with no outcome or prediction describing corals or coral reefs, it would not be included.

Impact of cold stress is included as an ocean warming evidence in this map to highlight the research on corals exposed to cold stress, as cold stress has been shown to bleach coral and may have led to mortality of coral populations.

Studies focused on ocean acidification and its interaction with ocean warming were excluded from this systematic map. Studies addressing both ocean acidification and ocean warming independently were included, but only the ocean warming data were included for this systematic map.

In other eligibility criteria, the following were changed:

Modelling studies without observation/experiment were excluded.

Study type: Studies that include original empirical data were included. Theoretical papers were excluded from the study. Non-primary research were not included for this systematic map.

Theoretical papers were excluded from this map, and they were not discussed at a separate section of this study. They were presented alongside other excluded literature in Additional file 2.

Studies with study periods ending before 2010 were excluded. If the study period began before 2010 but ended after 2010, the study is included but only data from that study period (or any period after 2010) were included.

### Search strategy

Ten benchmark articles were selected a priori for the development of the search string.

These 10 articles were selected based on their relevance to the topic, covering both climatology and subtropical and temperate coral systems under the influence of ocean warming (Additional file 3). A series of search strings were tested to test their capability to capture the benchmark articles. The Boolean operator search string was first developed on Scopus and translated to the Web of Science database after it successfully captured all 10 benchmark articles on Scopus. To capture recent studies, the search results were limited to publications on and after 2010 and the search was limited to publications in English only. The decision of limiting search results to publications since 2010 was made to capture the snapshot of most recent research. In database search accessed on Web of Science, we have utilised Science Citation Index Expanded under the Web of Science Core Collection. Both database searches were conducted on 15th June, 2023.

### Search strings

#### Bibliographic databases

Scopus: TITLE-ABS-KEY ((coral* OR “coral reef*”)) AND TITLE-ABS-KEY ((“ocean warming” OR “marine heatwave*” OR “marine heat wave*” OR mhw* OR “degree heating week*” OR dhw* OR “heat stress*” OR stress OR tropicali?ation OR temperature* OR “climate change”)) AND TITLEABS- KEY ((mortal* OR surviv* OR health* OR diseas* OR grow* OR reprodu* OR cover* OR tropicali?* OR shift* OR habitat* OR increase* OR declin* OR decreas* OR impact* OR threat* OR bleach* OR acclimati?* OR respons*)) AND TITLE-ABS-KEY ((marginal OR “high latitude” OR temperate OR subtropic* OR extratropic*)) Followed by a removal of articles published prior to 2010.

Web of Science: (All Fields) ((coral* OR “coral reef*”)) AND (All Fields) ((“ocean warming” OR “marine heatwave*” OR “marine heat wave*” OR mhw* OR “degree heating week*” OR dhw* OR “heat stress*” OR stress OR tropicali?ation OR temperature* OR “climate change”)) AND (All Fields) ((mortal* OR surviv* OR health* OR diseas* OR grow* OR reprodu* OR cover* OR tropicali?* OR shift* OR habitat* OR increase* OR declin* OR decreas* OR impact* OR threat* OR bleach* OR acclimati?* OR respons*)) AND (All Fields) ( ( marginal OR “high latitude” OR temperate OR subtropic* OR extratropic*)) Followed by removal of articles published prior to 2010.

#### Web-based search engines

Grey literature was searched on Open Access Theses and Dissertations (OATD) database on 19th September 2023, with the following search string used, which in pilot testing has yielded 9 results: coral AND (subtropical OR temperate) AND “ocean warming”. The included results were limited to articles and reports published since 2010 to cohere with the rest of the searches. Grey literature included was limited to research theses, pre-review reports, and open access scientific studies (herein defined as grey literature selected for inclusion in systematic mapping) while government reports were excluded due to the inconsistency in availability of these reports between regions.

#### Comprehensiveness of the search

All 10 manually collected benchmark articles were returned in the Scopus search, ensuring the search string to be sensitive enough for this systematic mapping study. The list of the 10 benchmark articles and record of development of the search string for database searches can be found in Additional file 3.

#### Screening strategy

The first screening was conducted including and excluding articles by titles and abstracts using Rayyan software [40] and was undertaken by two reviewers independently on 1,218 titles and abstracts. Articles included in the first round of screening were screened again in the second phase of the screening by full text. Where full text did not meet the selection criteria, they were excluded from the data extraction. A pilot screening was performed by two of the authors to assess the coherence of articles screening agreement. Articles on Rayyan were selected at random for reviewers to quickly screen through the titles and abstracts (30/1218, 2.46%). Consistency checks were then performed for both stages on 30 titles and abstracts (79.4% agreement rate). Subsequent screening of the remaining titles and abstracts (n = 1188) was performed by the same two reviewers in parallel independently. Reviewer 1 and Reviewer 2 disagreed on 129 articles, and these were marked by the two reviewers as “Maybe” and passed on to the third independent reviewer to make a final decision. After title and abstract screening, 197 retrievable full texts were subject to the full text screening for decision on final inclusion data extraction.

Full text screening was performed using a Google Form collaboratively by 6 reviewers for data collection from articles. All 197 retrievable full texts were screened individually first on abstract level by all reviewers to decide on including or excluding the full text for data extraction, in the case of discrepancies between decisions amongst reviewers, they were flagged and discussed by all six reviewers to reach a final decision. If the full text was included, then data would be extracted. If the full text was excluded, then the exclusion reason(s) for the full text would be provided in Additional file 2. The full texts marked for inclusion were allocated and distributed to the six reviewers for data extraction using the Google Form. The inclusion/exclusion decision and data were cross checked by one author (MLH) to ensure that all extracted information is coherent. After this, 90 studies were included in the systematic map.

#### Eligibility criteria

Articles from the databases were screened manually according to the following PECO criteria:

Population: Subtropical and Temperate Coral Systems and Coral Reefs

**Fig. 1:**
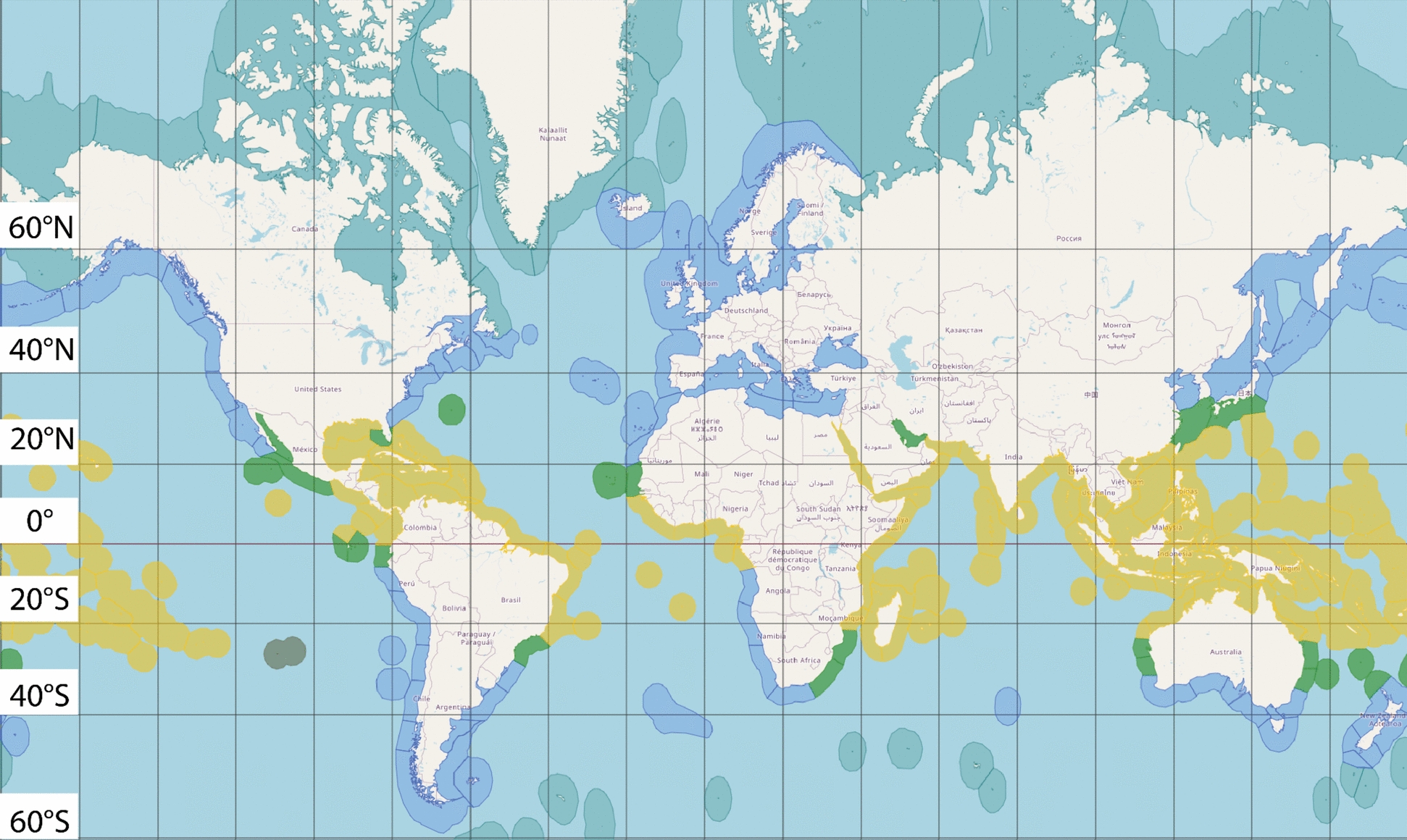
Map showing tropical ecoregions (Yellow), subtropical ecoregions (Green), temperate ecoregions (Blue) and arctic ecoregions (Light Blue). The grey area between 20°S and 40°S to the left side of the figure is the Easter Island, where both tropical and subtropical ecoregions can be found [23, 32]

- Studies focused on or including any subtropical and temperate coral systems and coral reefs were included. Subtropical and temperate coral systems and coral reefs are found in subtropical and temperate ecoregions respectively. In this systematic map, the subtropical and temperate ecoregions are defined as in Fig. 1 and Additional file 4 [23, 32]; this includes marginal and high latitude coral systems. Marginal reefs are where the environmental conditions are marginal or close to a threshold for the survival of the coral species, meaning that the living conditions are less than ideal but still feasible for coral species to survive [41]. High latitude reefs are reefs in regions above and below 28°N and 28°S respectively, referred in work by Beger et al. [23] and the henceforth figure and table (Fig. 1 and Additional file 4).
- Studies focused solely on tropical, polar and/or deep-sea corals, kelp forests and fishes were excluded.
- Corals here are defined as marine organisms belonging to the class Octocorallia and Hexacorallia. “Coral reefs” is used here to describe reefs with reef building corals.
- If a study describes the impact of ocean warming on marine organisms living on a subtropical/temperate coral reef with no outcome or prediction describing corals or coral reefs, it would not be included.

Exposure: Ocean Warming

- We included studies focused on the effects of exposure to ocean warming, specifically empirical ocean warming studies that utilise degree heating weeks and the definition of marine heatwaves as defined by Hobday et al. (2016) [36], where:
  o SST exceeds the bleaching threshold of 4 degrees Celsius.
  o SST exceeds the 90th percentile of the local climatological value for the time of year based on 30 years of climatology data [36].
- Studies focused on ocean acidification and its interaction with ocean warming were excluded from this systematic map. Studies addressing both ocean acidification and ocean warming independently were included, but only the ocean warming data were included for this systematic map.
- Impact of cold stress is included as an ocean warming evidence in this map to highlight the research on corals exposed to cold stress, as cold stress has been shown to bleach coral and may have led to mortality of coral populations [42].

Comparator: Effects before and after recorded event/exposure. Control/exposure experimental studies are included.

Outcomes: Mortality and bleaching, habitat changes, threats, acclimatisation of subtropical and temperate coral systems.

- We included all studies that address the physiological effects and ecological effects ocean warming have on the coral systems and reefs—this includes mortality, bleaching, changes in habitats (e.g., destruction of reef structures), threats (e.g., increase in species that pose threats to corals and coral reefs). Topics addressing the acclimatisation of species were also considered.
- Measured outcomes, predicted outcomes and predictions were included.

Other eligibility criteria

- Study type: Studies that include original empirical data were included. Theoretical papers were excluded from the study. Non-primary research were not included for this systematic map.
- Modelling studies without observation/experiment were excluded.
- Duplicated data (data that was reported in other publications) were excluded.
- Theoretical papers were excluded from this map, and they were not discussed at a separate section of this study. They were presented alongside other excluded literature in Additional file 2.
- Studies with study periods ending before 2010 were excluded. If the study period began before 2010 but ended after 2010, the study is included but only data from that study period (or any period after 2010) were included.

### Reporting screening outcomes

During the full-text screening, studies that were rejected from the process and the respective reasons were recorded. Reasons for exclusion of individual articles are listed in Additional file 2. A flow diagram was used to record and visualise the number of studies assessed and rejected at each stage.

### Study validity assessment

No study validity assessment of studies has been performed for this systematic map.

### Data coding strategy

**Fig. 2:**
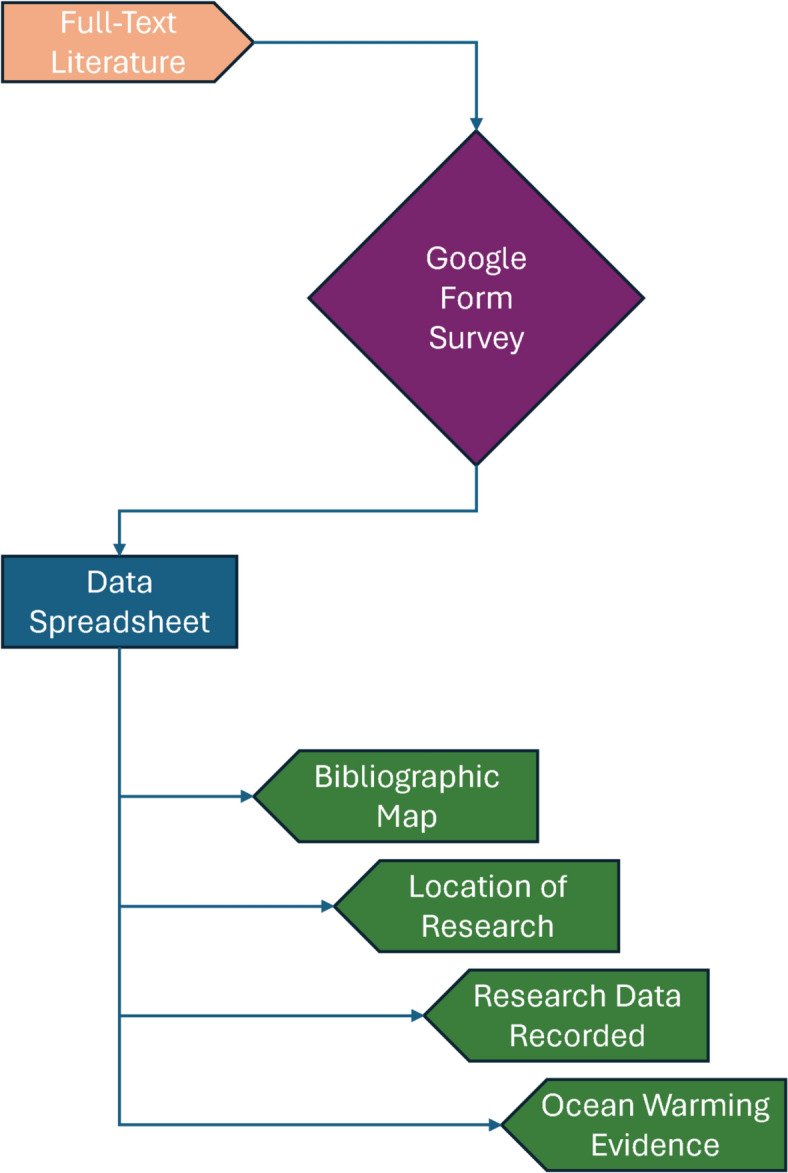
The data mapping methods flow chart for this systematic map

Data extraction was conducted collaboratively by six reviewers after being individually screened by all six reviewers as outlined in “Screening Strategy” section. Included full texts were allocated to the six reviewers for data extraction using a Google Form questionnaire, and the data were exported to a spreadsheet (Fig. 2). For reporting of the systematic map, we have placed different data types under the four categories described below:

- Bibliographic Map
  o Title, DOI, number of citations, authors’ information, year of publication, journal, keywords
- Location of research
  o Research ecoregions, type of location, development status, species
- Research data recorded
  o Temperature data recording methods, study type, study timeline, environmental parameters, availability of temperature data
- Ocean warming evidence
  o Extreme weather events recorded, stressors, severity, extreme weather events timeline, study outcome or prediction

Questions asked on the Google Form can be found in Additional file 5. This data extraction step also served as a full-text screening where final checks for inclusion eligibility were conducted. Additionally, we recorded the exclusion reason of the excluded full-text articles (Additional file 2).

Extracted data is provided in Additional file 6, which also serves as the master data file for this systematic map. Additional file 7 includes the metadata (i.e., descriptions of all extracted variables).

### Data mapping method

The primary product of this systematic map is a searchable database provided as a Microsoft Excel file (Additional file 6). The data are divided into four sheets by categories previously described in “Data Coding Strategy”. All columns within the database can be filtered to return data that meet the user’s demands. Multiple filters can be applied at a time to narrow the search results. Meta data for the columns are provided in Additional file 7.

Results are presented in this report as a series of statistics, bar charts, donut plots, heat map, and map visualisation to describe the statistics and spatial extent of evidence collated. We describe the quantity of evidence available for bibliography, location, research data, outcomes and ocean warming evidence in this report, and highlighting knowledge clusters and gaps related to our primary research question.

### Demonstrating procedural independence

As some of the authors of this review may have been authors of some of the studies to be reviewed in this work, their studies were independently assessed by other reviewers without discussion with the study authors to ensure the independence of the review.

**Reporting**. Methods outlined in previously published protocol were followed.

Results in this systematic map are presented often in “occurrences” and “studies” or “articles”. “Occurrences” refers to the occurrence of a subject mentioned in a study. For example, for the ecoregions in a single study, if the study contains three ecoregions under the same province, it would be reported as three occurrences in one study. “Studies” and “articles” are used interchangeably in this systematic map. Sum of “occurrences” could be greater than the number of included studies, while sum of “studies” and “articles” is always equal to the number of included studies.

In Additional file 6, “N/A” is used in cells where no applicable data were extracted.

All figures on this systematic map were created using R version 4.2.2 [43] on R Studio version 2023.06.0 [44], as well as RAWGraphs [45]. Several packages in R were used in the creation of figures, including ggplot2 (3.4.4) [46], wordcloud (2.6) [47], tidyverse (2.0.0) [48], dplyr (1.1.4) [49], reshape2 (1.4.4) [50], viridis (0.6.5) [51], RColorBrewer (1.1.3) [52], leaflet (2.2.1) [53], htmlwidgets (1.6.4) [54], webshot (0.5.5) [55], and tm (0.7.11) [56]. Organisation of plots into multi-panel figures were done with the use of Adobe Illustrator 2024 (Version 28.5, Adobe). Word cloud of keywords was created by homogenising keywords from included literature in a CSV file before being visualised using RStudio [44, 47].

### Review findings

**Fig. 3:**
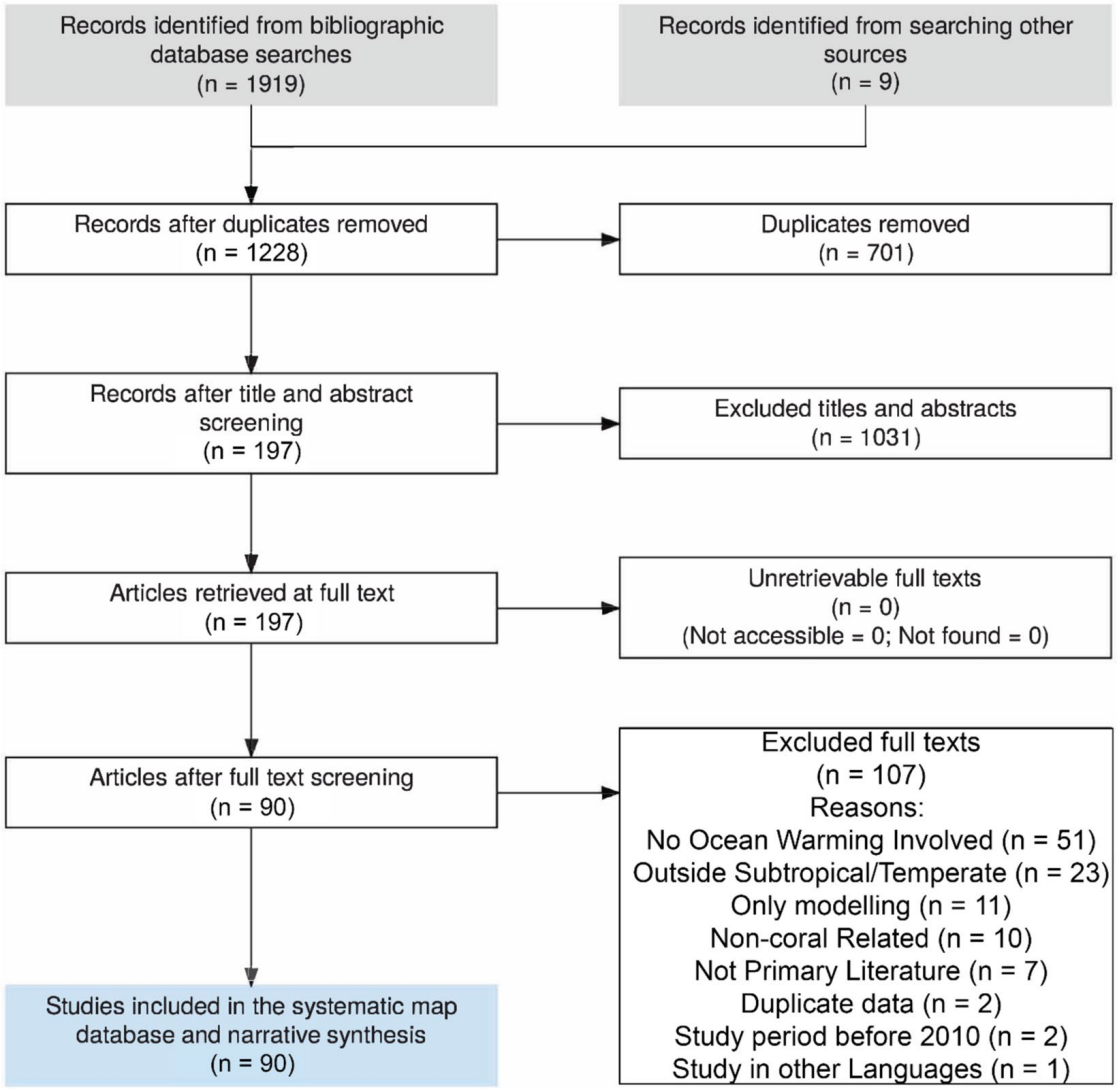
Flow diagram of this systematic map, detailing the process of removal literature per stage. Created with ROSES reporting flow diagram tool

**Data characteristics**. After database searches on Scopus and Web of Science, the search string had captured 877 and 1,042 results from the two databases respectively. After removal of duplicates, 1,228 unique studies remained. 197 studies were included after first stage of screening by title and abstract, and were screened at full-text level. After the full-text screening, 90 studies met the inclusion criteria for the purpose of this systematic map (Fig. 3). All 90 studies were extracted for the systematic map. Search on Open Access Theses and Dissertations database had captured nine studies. Originally seven of the grey literature were excluded as these only focus on tropical coral reef. The remaining two were eventually excluded as duplicated data, since the theses were published, and the published versions were already included in this systematic map (Additional file 2).

**Fig. 4:**
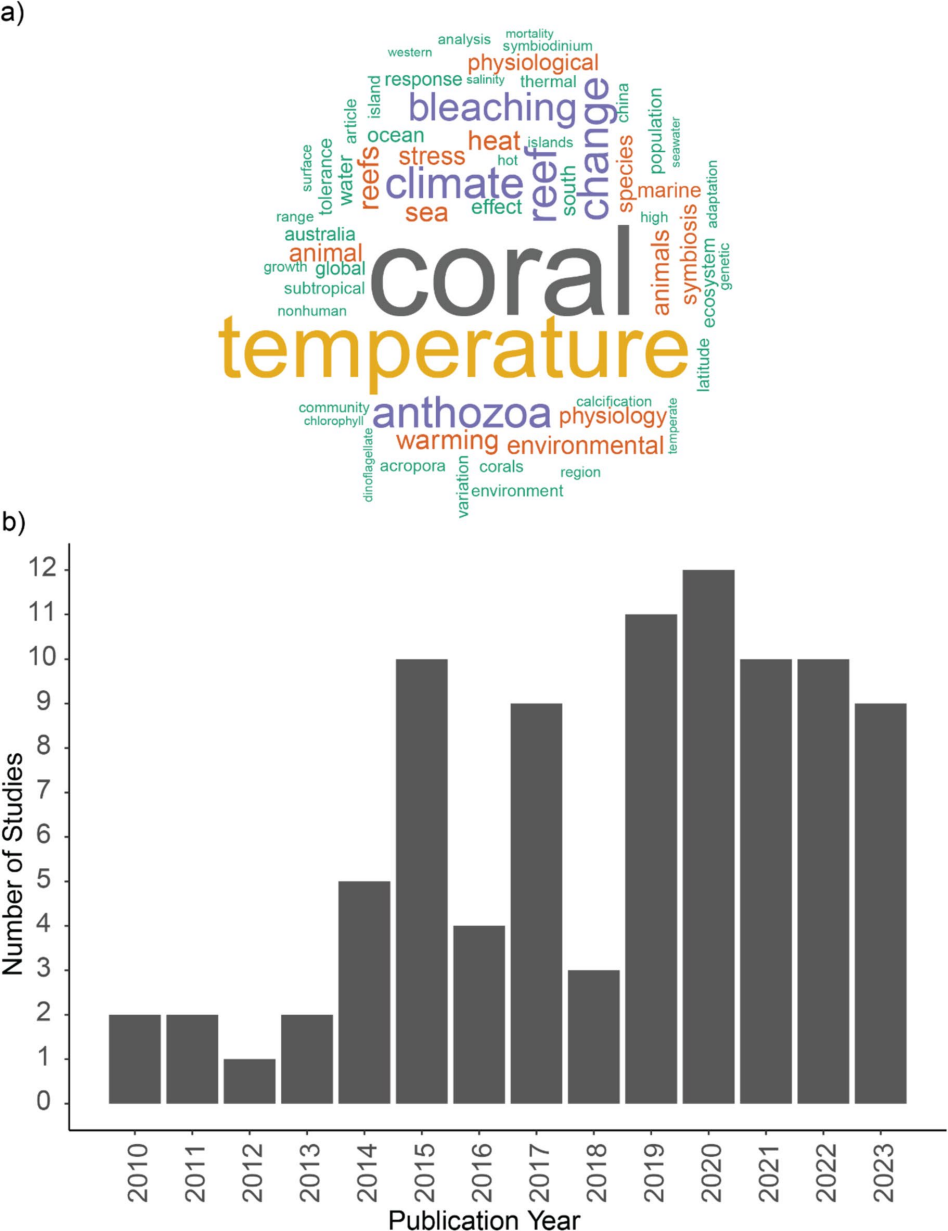
**a** Word cloud featuring keywords appearing at least 10 times from the literature included in this systematic map. **b** The number of publications by year. Note that this data is up to June 2023, and publication number for 2023 may be higher at time of publication

**Study types.** 44 studies were identified (48.9%) that were observation-based, three studies (3.3%) included both observational and modelling evidence. 35 studies (38.9%) were identified as experimental, with 7 studies (7.7%) including both experimental and observational evidence and one study (1.1%) including both experimental and modelling evidence. The most common article keywords used in included studies were “coral”, “temperature”, “bleaching”, “change”, “reef”, “climate”, and “anthozoa” (see a word cloud of keywords that appeared at least 10 times from literature included in this systematic map in Fig. 4a).

**Fig. 5:**
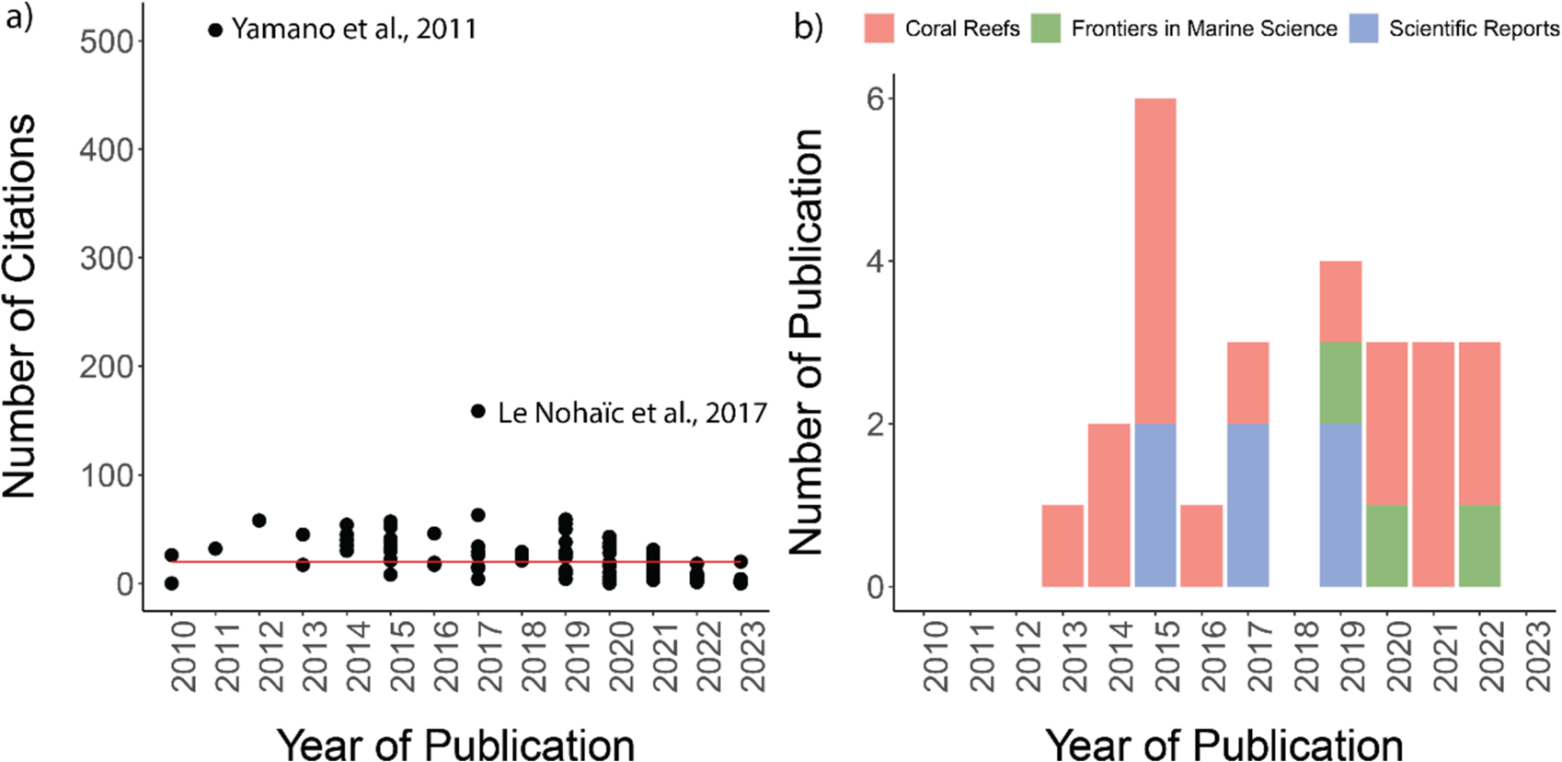
**a** Number of citations of literature by years. Red line is the median of number of citations across the literature included in this systematic map. **b** The top 3 journals by number of publications by year

**Fig. 6:**
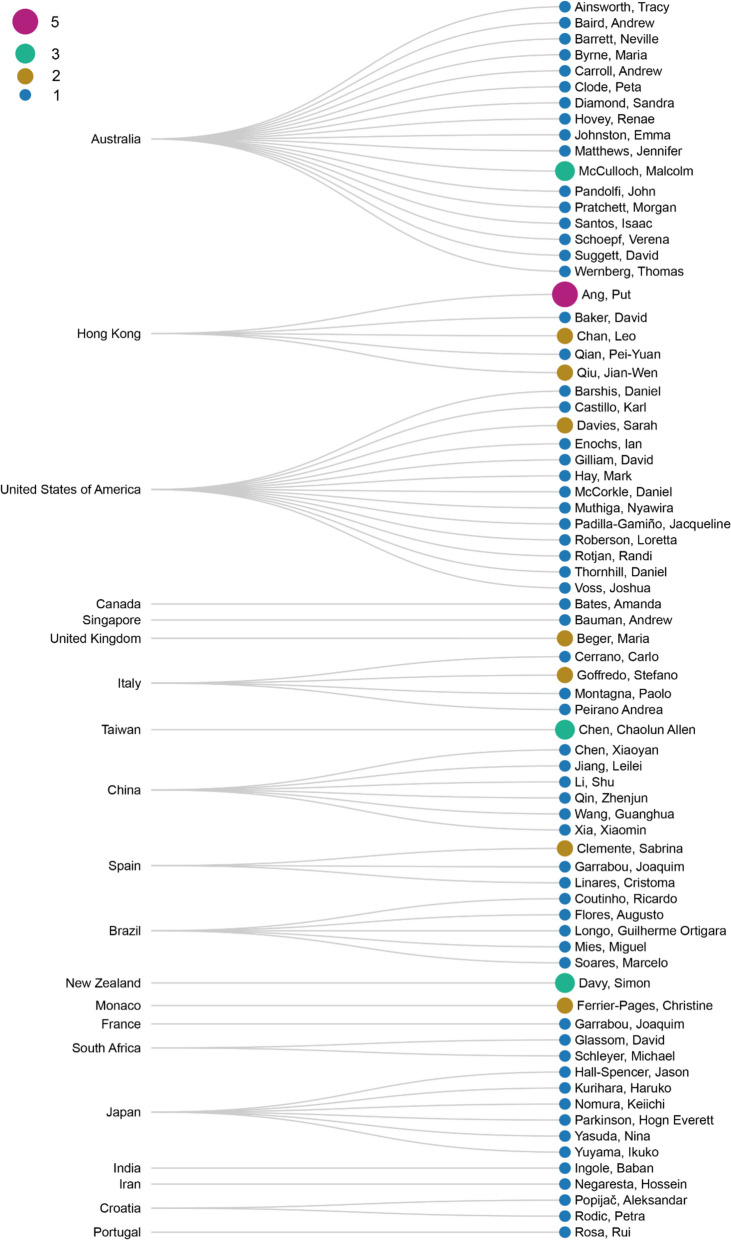
Linear dendrogram showing relations of last authors to countries their institutions belong to

**Fig. 7:**
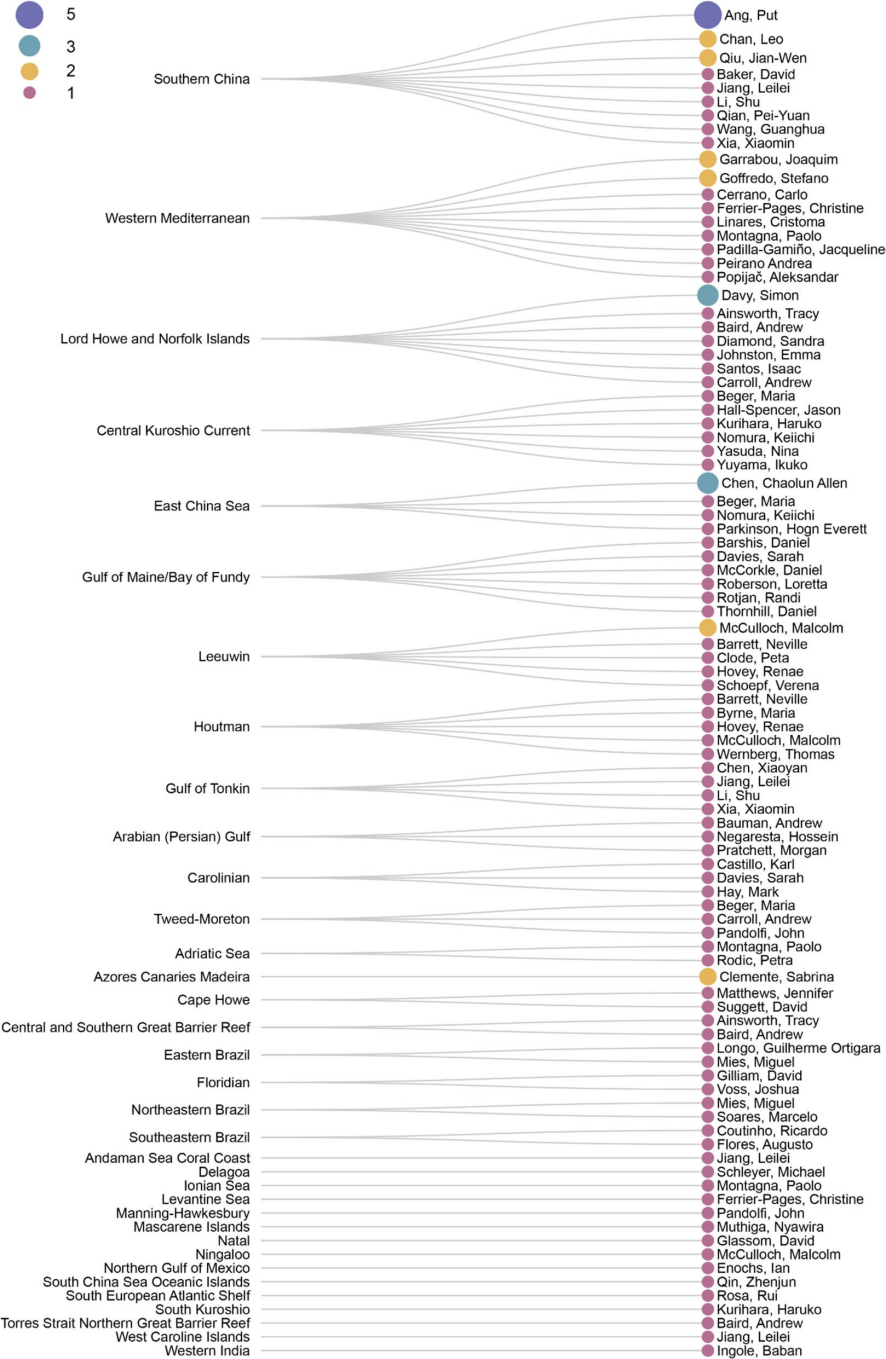
Linear dendrogram showing relations of last authors to ecoregions their literature focused on

**Bibliographic information**. Increasing research effort from 2013 to 2015 is reported (a total of 19 publications during 2013—2015) following by a drop to four publications in 2016 and increase to nine publications in 2017 (Fig. 4b) and has reached a peak of 10 and nine publications in 2022 and Mid-2023 respectively. In terms of number of citations, two studies stood out, having 510 citations (study by Yamano et al., 2011 [25]) and 159 citations (study by Le Nohaïc et al., 2017 [57]), respectively. Other publications included in this systematic map are less cited (Median = 20) (Fig. 5a). Last authors with more than one publication are associated with institutions in Hong Kong (three authors), Australia (one author), United States of America (one author), United Kingdom (one author), Italy (one author), Taiwan (one author), Spain (one author), New Zealand (one author), and Monaco (one author). In this systematic map, we took the assumption that the last author is a senior author, however, we acknowledge that it may not be the case for all fields for literature included in this map. Most last authors are publishing only intermittently, with only one publication where the authors’ names appear as last author (Fig. 6). Figure 7 shows the respective last authors and the ecoregions of where the included publications focused on. The leading locations are in Southern China, with P. Ang having the highest number of publications focusing on the area. This is followed by numerous research in Western Mediterranean, with authors J. Garrabou and S. Goffredo, both sharing the same number of publications on the area. Lord Howe and Norfolk Islands came third in terms of number of last authors and their focus on the area, with S. Davy having the highest number of publications in the area.

In terms of all the authors regardless of position of first, last, or other co-authors, P. Ang has the highest number of publications (seven publications) in the literature included for this systematic map, followed by C. Ross with five publications. A list of authors, including A. Chui, X. Huang, C. Linares, M. McCulloch, J.W. Qiu, R. Tsang, and K. Yu, have four publications each (all extracted author names with the counts of their included works are shown in Additional file 8).

In terms of publication journals, the largest number of studies included come from Coral Reefs (17 studies), followed by Frontiers in Marine Science (seven studies), Scientific Reports (six studies) (Fig. 5b). Other journals include Science of the Total Environment (four studies), Diversity (three studies), Global Change Biology (three studies), Marine Ecology Progress Series (three studies), Marine Pollution Bulletin (three studies), PloS One (three studies), Proceedings of the Royal Society B: Biological Sciences (three studies), Bulletin of Marine Science (two studies), Ecology and Evolution (two studies), Journal of Experimental Marine Biology and Ecology (two studies), Limnology and Oceanography (two studies), Molecular Ecology (two studies) (Table 1 with all included articles).

**Table 1:** Number of publications by journals across literature included for this systematic map

Journal name	Number of publications
Coral Reefs	17
Frontiers in Marine Science	7
Scientific Reports	6
Science of The Total Environment	4
Diversity	3
Global Change Biology	3
Marine Biology	3
Marine Ecology Progress Series	3
Marine Pollution Bulletin	3
PloS One	3
Proceedings of the Royal Society B	3
Bulletin of Marine Science	2
Ecology and Evolution	2
Journal of Experimental Marine Biology and Ecology	2
Limnology and oceanography	2
Molecular Ecology	2
Applied microbiology and biotechnology	1
Aquatic Conservation: Marine and Freshwater Ecosystems	1
Aquatic Ecosystem Health & Management	1
Biodiversity and Conservation	1
Biogeosciences	1
Cell Stress and Chaperones	1
Ecography	1
Environmental Science and Pollution Research	1
Estuarine, Coastal and Shelf Science	1
Frontiers in Physiology	1
Geophysical Research Letters	1
Helgoland Marine Research	1
Integrative Organismal Biology	1
Journal of Experimental Biology	1
Journal of Heredity	1
Journal of Marine Science and Engineering	1
Journal of phycology	1
Marine Chemistry	1
Marine Environmental Research	1
Microorganisms	1
Nature	1
Pakistan Journal of Biological Sciences	1
PeerJ	1
Regional Studies in Marine Science	1
The Biological Bulletin	1

### Location of research

**Fig. 8:**
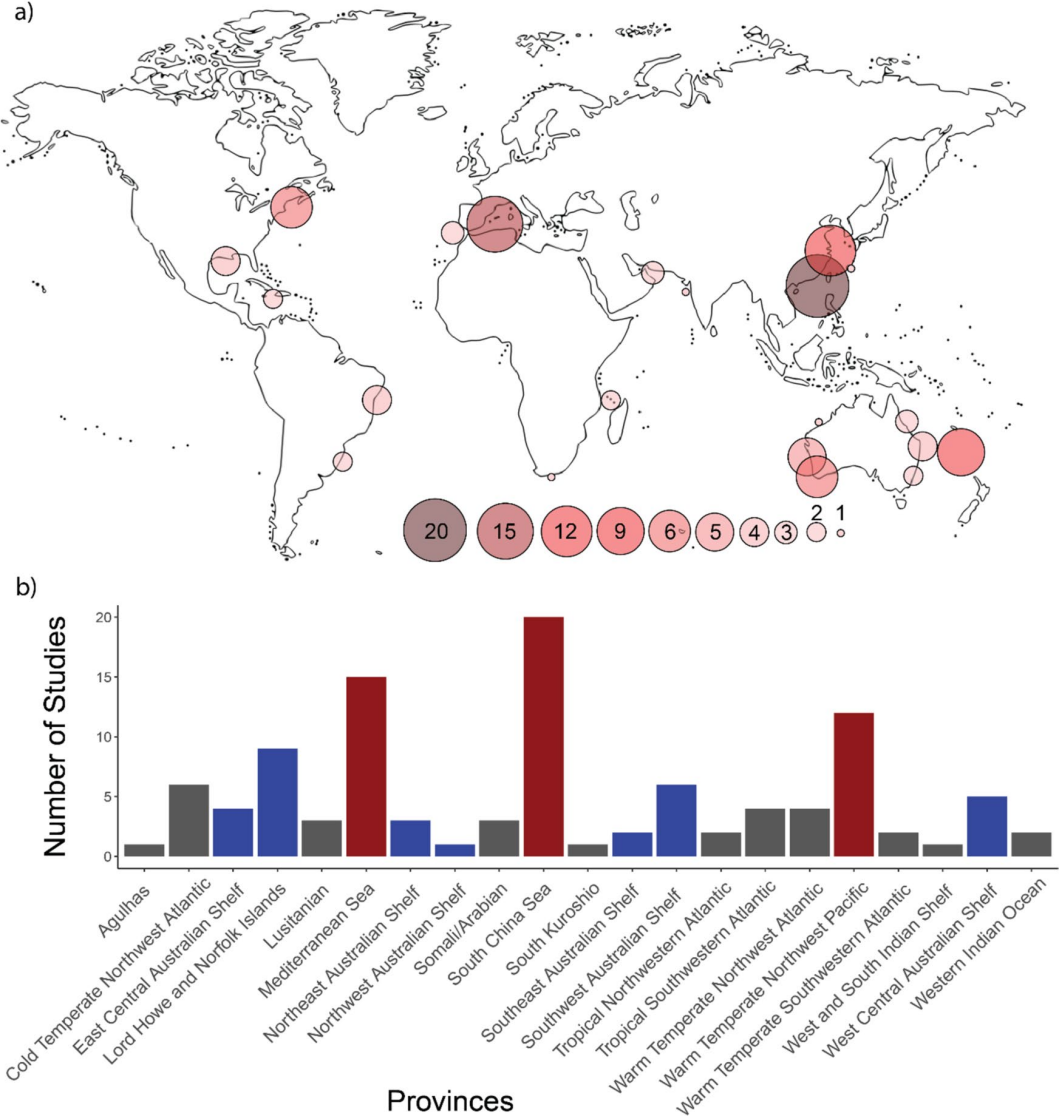
**a** World map showing the knowledge cluster by ecoregion. **b** Number of studies by provinces, showing top 3 provinces (Red) and provinces in Australia (Blue)

The provinces were identified, and subsequently the ecoregion where the included studies were conducted (Fig. 8). There were 6 studies that involve both tropical and subtropical ecoregions in Australia. Majority of studies were conducted in the province of South China Sea (20 occurrences in 17 studies, 18.9%), this includes the ecoregions of Southern China (15 studies, 16.7%), South China Sea Oceanic Islands (one study, 1.1%), and Gulf of Tonkin (four studies, 4.4%). The second highest province is Mediterranean Sea, consisting of 15 occurrences (within 13 studies, 14.4%), including the ecoregions of Western Mediterranean (11 studies, 12.2%), Adriatic Sea (two studies, 2.2%), Levantine Sea (one study, 1.1%), and Ionian Sea (one study, 1.1%). The province of Warm Temperate Northwest Pacific was the third highest occurring province, with 12 occurrences in 10 studies (11.1%) identified in the ecoregions of East China Sea (six studies, 6.7%), Central Kuroshio Current (six studies, 6.7%) (Fig. 8b).

When examining the provinces recorded from the included studies, seven provinces that are on the Australian continent were identified, including Lord Howe and Norfolk Islands (nine occurrences in nine studies, 10%), Southwest Australian Shelf (six occurrences in six studies, 6.7%), West Central Australian Shelf (five occurrences in five studies, 5.6%), East Central Australian Shelf (four occurrences in three studies, 3.3%), Northeast Australian Shelf (three occurrences in two studies, 2.2%), Southeast Australian Shelf (two occurrences in two studies, 2.2%) and Northwest Australian Shelf (one occurrence in one study, 1.1%). These provinces include the ecoregions of Lord Howe and Norfolk Island (nine studies, 10%), Leeuwin (six studies, 6.7%), Houtman (five studies, 5.6%), Tweed-Moreton (three studies, 3.3%), Manning-Hawkesbury (one study, 1.1%), Central and Southern Great Barrier Reef (two studies, 2.2%), Torres Strait Northern Great Barrier Reef (one study, 1.1%), Cape Howe (two studies, 2.2%), and Ningaloo (one study, 1.1%) (Fig. 8b and Table 2).

**Table 2:** Number of publications by ecoregions across this systematic map

Ecoregions	Belongs to province	Number of publications concerning the ecoregion
Southern China	South China Sea	15
Western Mediterranean	Mediterranean Sea	11
Lord Howe and Norfolk Islands	Lord Howe and Norfolk Islands	9
Leeuwin	Southwest Australian Shelf	6
Gulf of Maine/Bay of Fundy	Cold Temperate Northwest Atlantic	6
Central Kuroshio Current	Warm Temperate Northwest Pacific	6
East China Sea	Warm Temperate Northwest Pacific	6
Houtman	West Central Australian Shelf	5
Gulf of Tonkin	South China Sea	4
Arabian (Persian) Gulf	Somali/Arabian	3
Tweed-Moreton	East Central Australian Shelf	3
Carolinian	Warm Temperate Northwest Atlantic	3
Central and Southern Great Barrier Reef	Northeast Australian Shelf	2
Azores Canaries Madeira	Lusitanian	2
Southeastern Brazil	Warm Temperate Southwestern Atlantic	2
Floridian	Tropical Northwestern Atlantic	2
Eastern Brazil	Tropical Southwestern Atlantic	2
Cape Howe	Southeast Australian Shelf	2
Northeastern Brazil	Tropical Southwestern Atlantic	2
Adriatic Sea	Mediterranean Sea	2
Torres Strait Northern Great Barrier Reef	Northeast Australian Shelf	1
Northern Gulf of Mexico	Warm Temperate Northwest Atlantic	1
Levantine Sea	Mediterranean Sea	1
Natal	Agulhas	1
Western India	West and South Indian Shelf	1
West Caroline Islands	Tropical Northwestern Pacific	1
Andaman Sea Coral Coast	Andaman	1
South Kuroshio	South Kuroshio	1
Ningaloo	Northwest Australian Shelf	1
Ionian Sea	Mediterranean Sea	1
Mascarene Islands	Western Indian Ocean	1
Manning-Hawkesbury	East Central Australian Shelf	1
South China Sea Oceanic Islands	South China Sea	1
South European Atlantic Shelf	Lusitanian	1
Delagoa	Western Indian Ocean	1

Six studies (6.7%) were identified for the province of Cold Temperate Northwest Atlantic, where all ecoregions included are Gulf of Maine/Bay of Fundy. Tropical Southwestern Atlantic and Warm Temperate Northwest Atlantic recorded four occurrences each in 3 studies (3.3%) and 4 studies (4.4%) respectively, within the ecoregions of Eastern Brazil (two studies, 2.2%), North-eastern Brazil (two studies, 2.2%), Carolinian (three studies, 3.3%), and Northern Gulf of Mexico (one study, 1.1%). Tropical Northwestern Atlantic and Warm Temperate Southwestern Atlantic each has two occurrences, and two studies for each province (2.2%). The ecoregions under these two provinces, Floridian and South-eastern Brazil, respectively, have two studies each (2.2%) recorded.

The province of Somali/Arabian has three occurrences in three studies (3.3%), the included ecoregion is Arabian (Persian) Gulf (three studies, 3.3%). Similarly, the province of Lusitanian also recorded three occurrences in three studies (3.3%), in the ecoregion of Azores Canaries Madeira (two studies, 2.2%) and South European Atlantic Shelf (one study, 1.1%). Remaining recorded provinces are Western Indian Ocean (two occurrences in two studies, 2.2%) with the ecoregions of Mascarene Islands (one study, 1.1%) and Delagoa (one study, 1.1%); Agulhas (one occurrence in one study, 1.1%) with the ecoregion Natal (one study, 1.1%), and South Kuroshio (one occurrence in study, 1.1%), with one study (1.1%) in the ecoregion South Kuroshio.

Within the included studies, the majority (58 studies, 64.4%) of the studies were conducted in regions of developed economies (United Nations Country Classification, accessed 2024), with Australia (23 studies, 25.6%) having the highest number of studies published within the category. This is followed by the United States of America (12 studies, 13.3%) and Japan (seven studies, 7.8%). Within the category of developed economies, one study (1.1%) has also included an economy in transition (defined as industrialised countries that are undergoing process of transition to a market economy; these include some former Soviet Union republics [58]). Another study also encompasses a developed economy and developing economy (defined as countries with less developed industrial base and lower human development index relative to other countries [59]). For developing economies (31 studies, 34.4%), Hong Kong Special Administrative Region has the highest number of published studies (11 studies, 12.2%), followed by China (five studies, 5.6%) and Brazil (five studies, 5.6%).

**Fig. 9:**
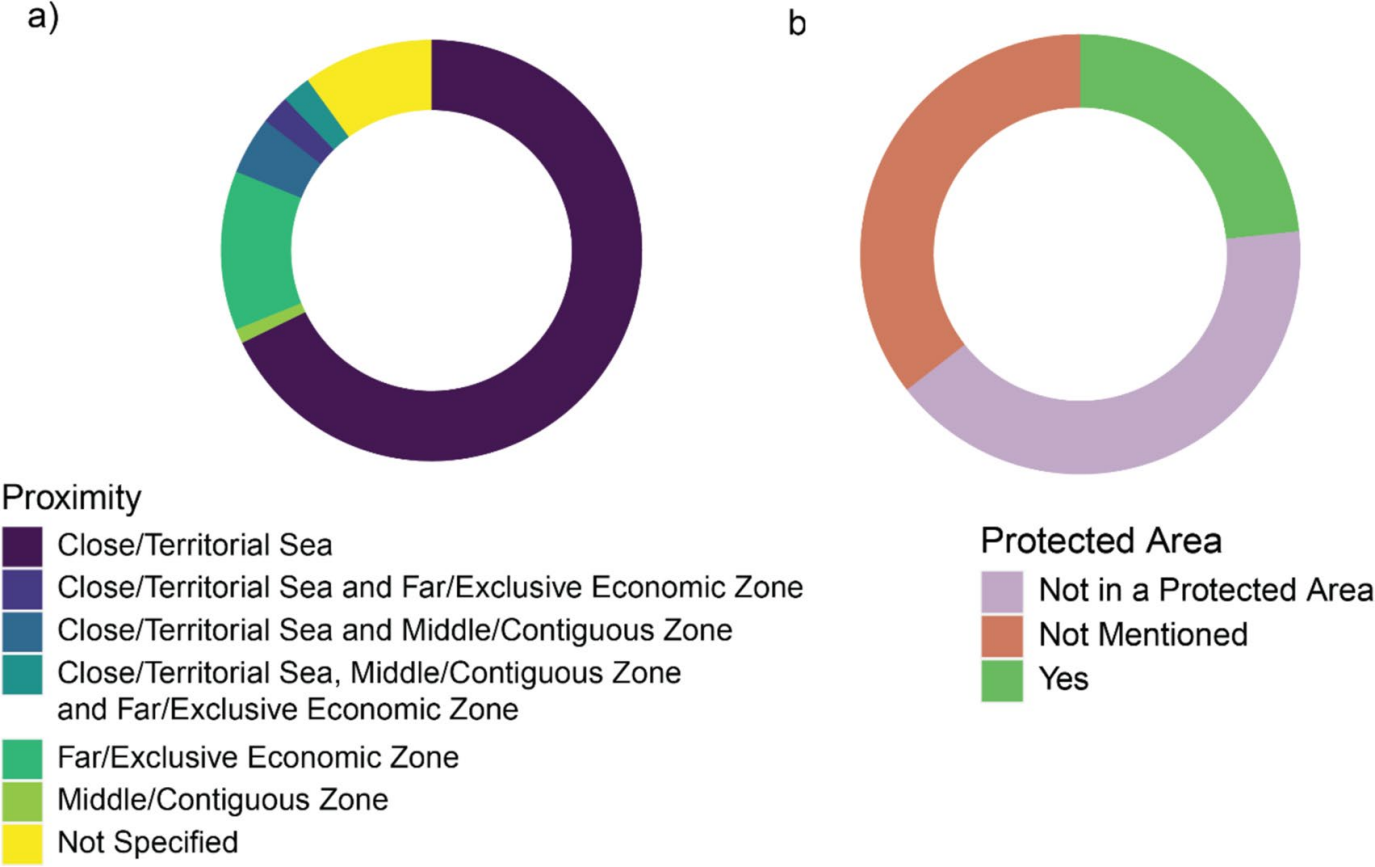
**a** Donut plot showing the proximity of the locations of studies to the closest EEZ across all literature in this systematic map. **b** Donut plot showing the proportions of studies that took place in a protected area

For proximity of the research site to the mainland (Fig. 9a), most studies involve close/territorial sea (69 studies, 76.7%). Middle/contiguous zone is represented in seven studies (7.8%) and far/exclusive economic zone in 15 studies (16.7%). Of all the studies involving close/territorial sea, four studies (4.4%) also involve middle/contiguous zone, two studies (2.2%) involve far/exclusive economic zone, and two studies (2.2%) involve both middle/contiguous and far/exclusive economic zone. Nine studies (10%) did not specify their proximity to the mainland (Fig. 9a) and 21 of the studies (23.3%) took place in protected areas (Fig. 9b). Protected areas are defined as areas with a clearly defined geographical space, recognised, dedicated, and managed through legal or other means to maintain the long-term conservation of ecosystems and cultural values associated with the location [60]. A list of the protected areas can be found in Table 3.

**Table 3:** List of identified marine reserve, protected area, or marine park

Name of Protected Area	Country	References
Abrolhos Marine National Park	Brazil	Ferreira et al., 2021
Blue Bay Marine Park	Mauritius	McClanahan et al., 2021
Columbretes Islands Marine Protected Area	Spain	Kersting et al., 2015
Medes Island Marine Protected Area	Spain	Kersting et al., 2015; Gómez-Gras et al., 2019; Crisci et al., 2017
Green Island Marine Research Station	Taiwan	Schutter et al., 2015
Jurien Bay Marine Park	Australia	Ross et al., 2021
Marmion Marine Park	Australia	Ross et al., 2021
Rottnest Island Marine Reserve	Australia	Ross et al., 2021
Shoalwater Islands Marine Park	Australia	Ross et al., 2021
Ngari Capes Marine Park	Australia	Ross et al., 2021
Lord Howe Island Marine Park	Australia	Steinberg et al., 2022; Davis et al., 2020; Oakley et al., 2022; Dalton et al., 2011
Malvan Marine Santuary	India	De et al., 2023
Scandola Nature Reserve	France	Crisci et al., 2017
Mljet National Park	Croatia	Kružić et al., 2015
Pedra da Risca do Meio	Brazil	Lucas et al., 2023
Pedra Vermelha Restricted-Access Site	Brazil	Lima et al., 2016
Solitary Islands Marine Park	Australia	Lachs et al., 2021; Dalton et al., 2011
Tung Ping Chau Marine Park	Hong Kong	Chui et al., 2015; Chui et al., 2016; Chui et al., 2017; Chui et al., 2023
Yan Chau Tong Marine Park	Hong Kong	Cai et al., 2018

### Methodological approaches

**Fig. 10:**
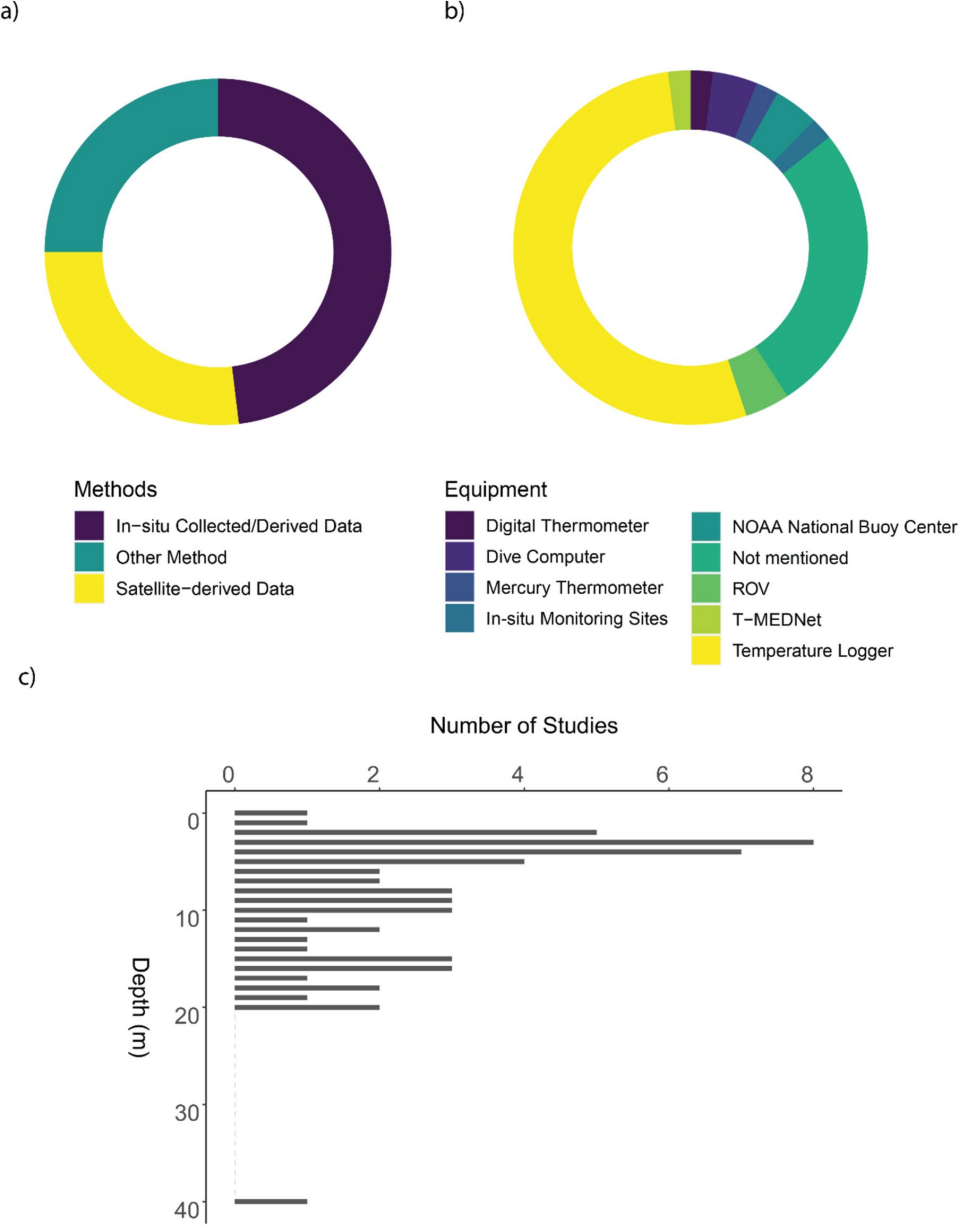
**a** Proportion of methods of temperature recording used across the literature included in this study. **b** Donut chart showing the proportion of equipment used for studies that recorded temperature in-situ. **c** The depth of temperature data collection

*Temperature Data Record*. When examining the methods reported by the study authors for obtaining ocean temperature records, 50 publications were found to have obtained their temperature data *in-situ* (deployment of loggers), 27 studies used remote sensing data (herein referred to satellite derived and SST, sea surface temperature) for temperature data. 26 studies used other methods of obtaining temperature data, these are *ex-situ* conservation studies (where temperature was set to match the experimental criteria) (24 studies) or with study design not clearly identified (two studies) in the literature (Fig. 10a). For *in-situ* collected data, data were collected using temperature loggers (26 studies), dive computers (two studies), NOAA National Buoy Center (two studies), remotely operated vehicles (ROV) (two studies), digital thermometers (one study), mercury thermometers (one study), local monitoring sites (one study), and using T-MEDNet as a temperature data sharing platform (one study) (Fig. 10b). 13 studies did not specify the method of recording temperature data. For temperature data availability, 51 of the 90 studies did not provide raw temperature data for access, with the remaining 40 studies providing full access to raw temperature data (see Additional file 9).

*Study depth*. For study depth and/or depth of data collection, majority of temperature data were obtained at depth of 0 to 20 m below sea surface (Fig. 10c), with one temperature data recording at a depth of 40 m (T-MEDNet). The most common depth for temperature collection is 3 m (eight occurrences), followed by 4 m (seven occurrences) and 2 m (five occurrences).

**Fig. 11:**
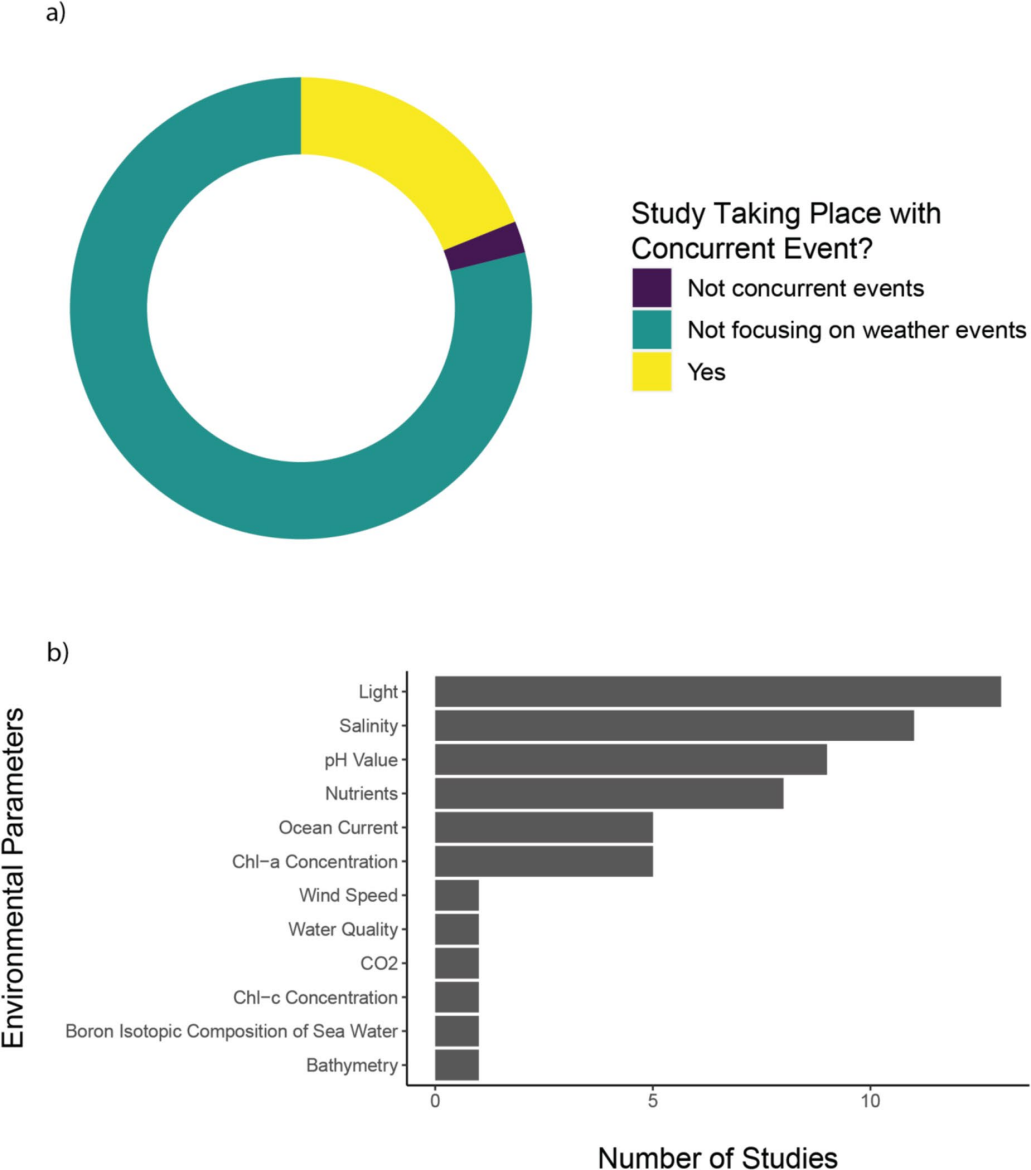
**a** Portion of publication focusing on weather event. **b** Other parameters recorded in the literature covered in this systematic map

**Fig. 12:**
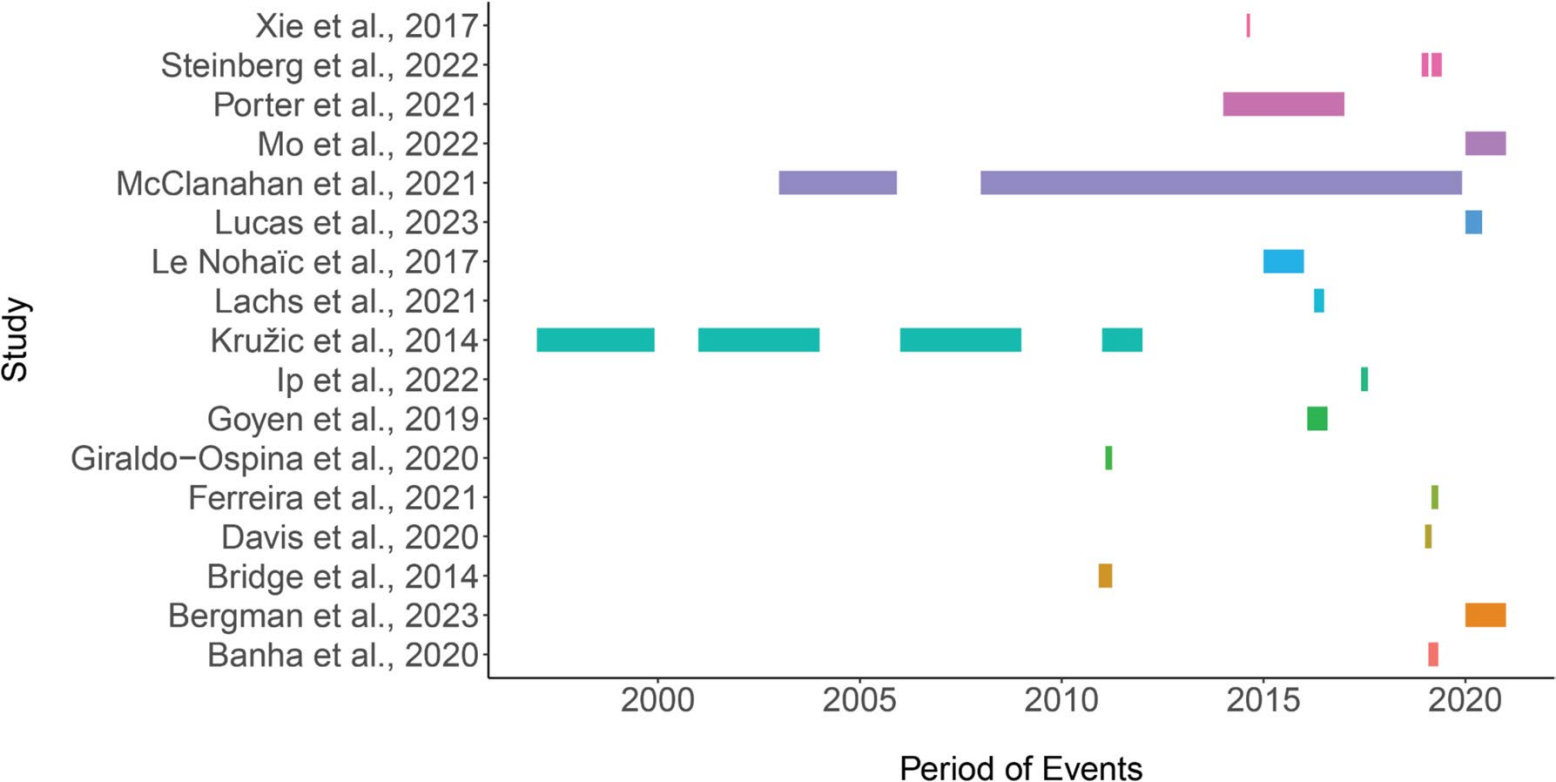
Timeline of recorded extreme weather events from studies that took place concurrently with the event

*Type and timing of study*. 17 studies of the 90 studies included are reported to have taken place concurrently with extreme weather or climate events (Figs. 11a and 12) and focused on reporting evidence associated with the specific event occurring, while two further studies report extreme weather events but were not conducted concurrently with the events. The majority of studies (71 studies) were not describing any specific extreme weather events.

*Other environmental data*. From the included studies, most observed environmental parameters (Fig. 11b) are light (13 studies), salinity (11 studies) and pH values (nine studies), while other parameters include nutrients (eight studies), ocean current (five studies), chlorophyll a concentration (five studies), wind speed (one study), water quality (one study), CO_2_ concentration (one study), chlorophyll c concentration (one study), boron isotopic composition of sea water (one study), and bathymetry (one study).

**Fig. 13:**
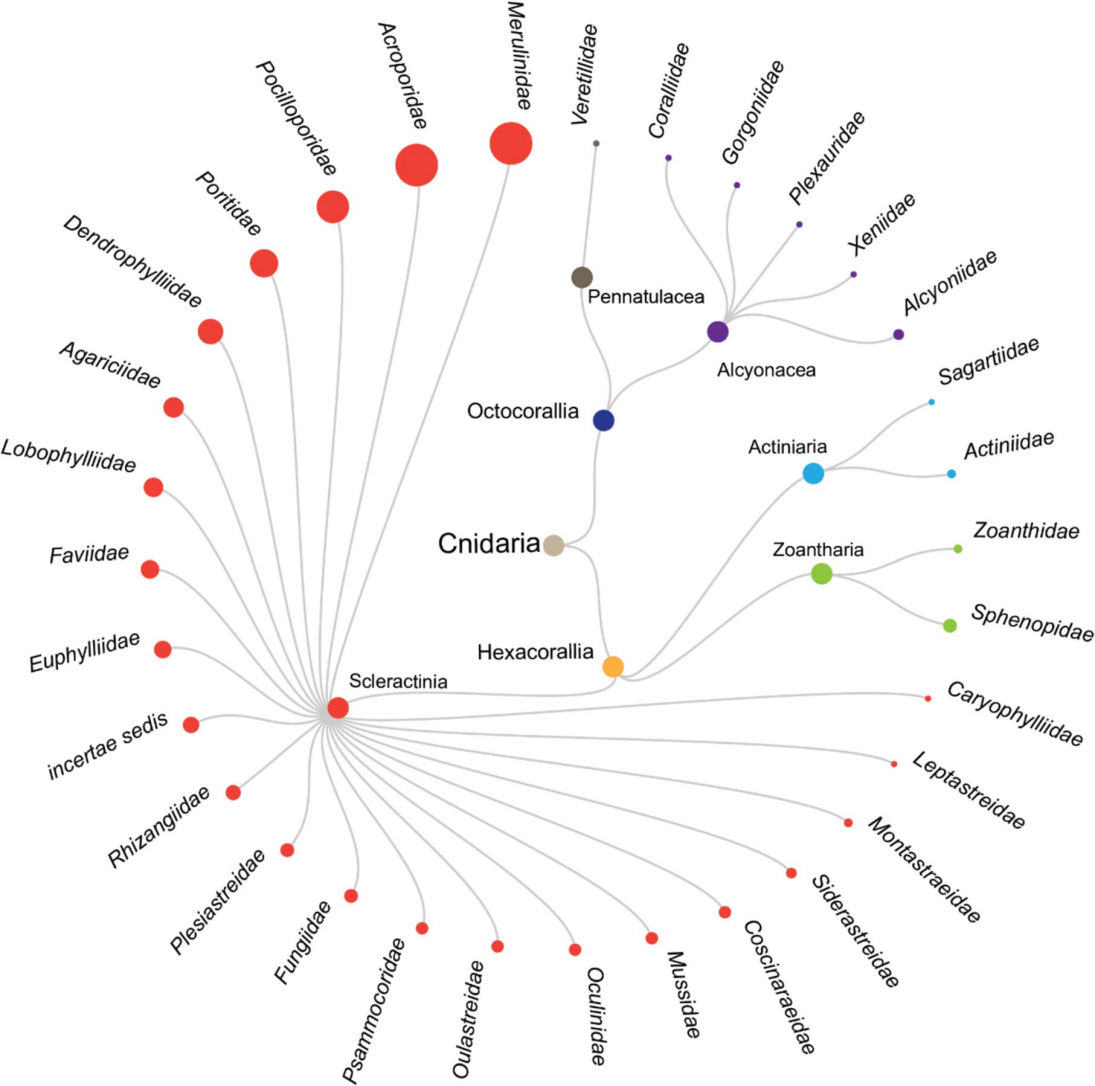
Dendrogram showing the classes and families studied by the literature recorded in this systematic map

*Study species.* For coral taxa studied, Hexacorallia was the most dominant studied Cnidarian class, with a total of 132 species studied. Under the class Hexacorallia, the stony coral order Scleractinia was dominant within the class, where 124 species studied were under order Scleractinia. Zoanthoaria was the next most studied order, with five species in total and the order Actiniaria had three species studied. Octocorallia was the second dominant studied Cnidarian class, with a total of eight species within the phylum. Alcyonacea was the most studied order within the Octocorallia class, where seven species were studied across the literature mapped in this systematic map. The last order to be included in this map is Pennatulacea, with just one species being studied across the literature (Fig. 13).

Within the stony coral order Scleractinia, Merulinidae and Acroporidae were the families studied most frequently in the literature collected, each with 48 occurrences. This is followed by Pocilloporidae (28 occurrences) and Poritidae (21 occurrences). Other families with at least 10 occurrences are Dendrophylliidae (17 occurrences), Agariciidae (11 occurrences) and Lobophylliidae (10 occurrences). Species level information can be found in Additional file 10.

### Ocean warming evidence

**Fig. 14:**
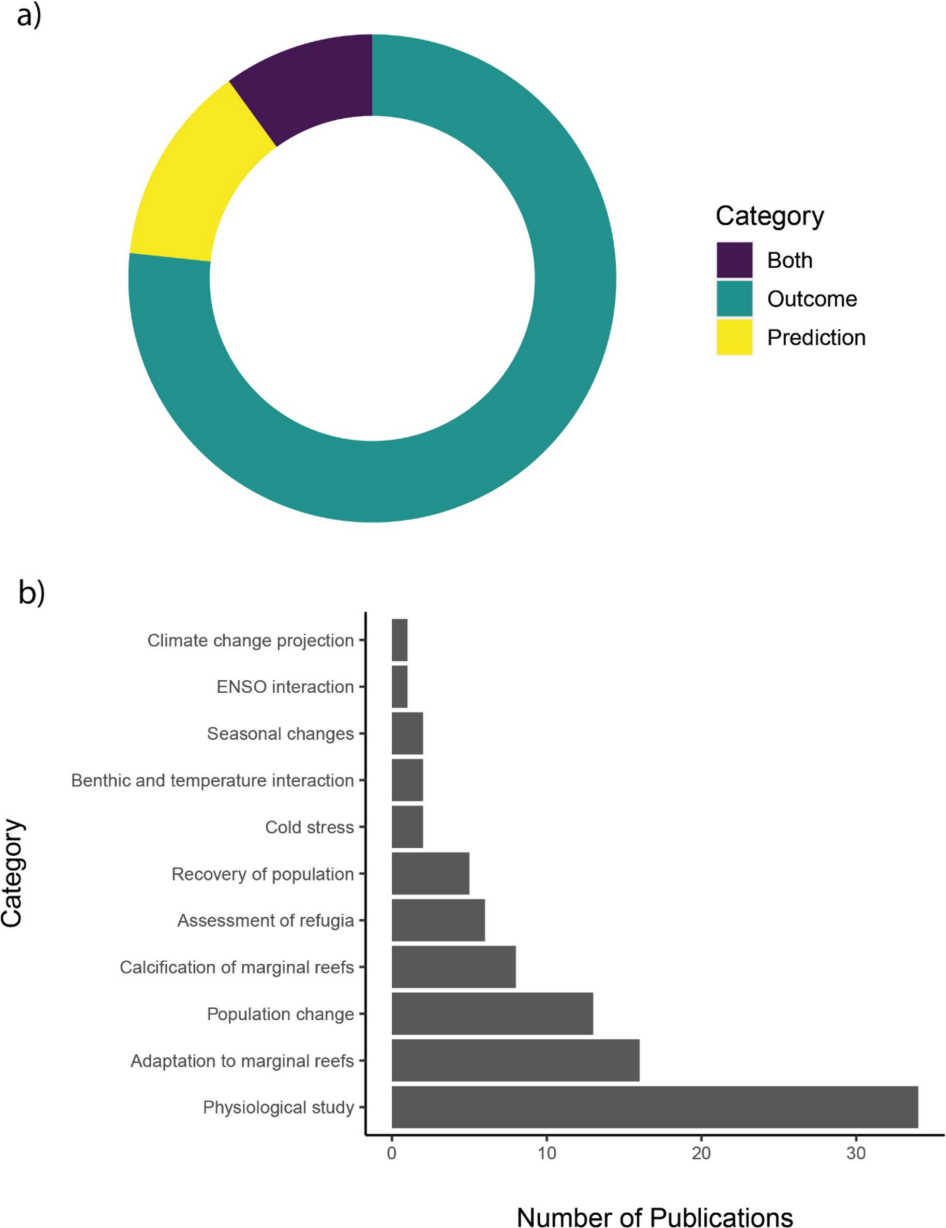
**a** Proportion of literature included in this systematic map that include outcome and/or prediction. **b** Categories of outcome/prediction and their number of publications respectively

**Fig. 15:**
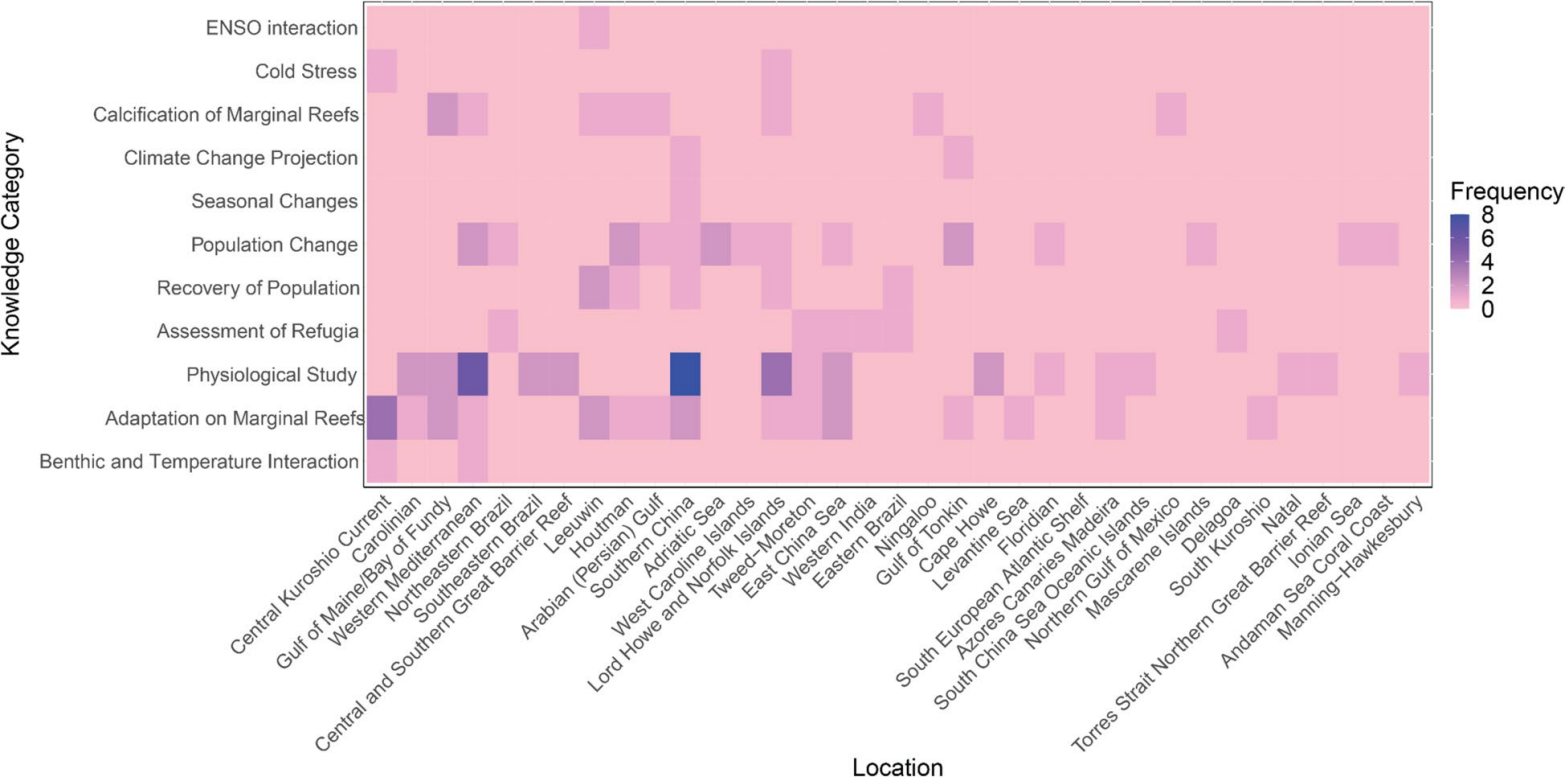
Heatmap of the knowledge category with respect to ecoregions from studies included in this systematic map

*Outcomes.* 69 studies (76.7%) provided an outcome associated with the study conclusion, while 12 studies (13.3%) provided prediction in their conclusion, and the remaining nine studies (10%) provided both outcome and prediction (Fig. 14a). From the outcomes and predictions, most studies focus on reporting on physiology of coral systems under thermal stress (34 studies, 37.8%), followed by investigations into adaptation of coral species into marginal reefs (16 studies, 17.8%), changes in coral populations (12 studies, 13.3%), calcification of reefs (eight studies, 8.9%), assessment of potential refugia (six studies, 6.7%), recovery of population (five studies, 5.6%), climate change projections on threats to marginal reefs (one study, 1.1%), impact of cold stress on coral (two studies, 2.2%), seasonal changes with resilience of corals (two studies, 2.2%), and ENSO interactions on regional scale mass bleaching (one study, 1.1%) (Fig. 14b). A heatmap (Fig. 15) shows the main outcomes studied and the respective ecoregion where these studies were conducted. Specifically, physiological studies carried out in Southern China are the most frequent (eight occurrences in eight studies, 8.9%), forming the main knowledge cluster. Another major cluster observed is made of physiological studies in Western Mediterranean (six occurrences in six studies, 6.7%). This is followed by a cluster of physiological studies in Lord Howe and Norfolk Islands (four occurrences in four studies, 4.4%) and studies on adaptation to marginal reefs in Central Kuroshio Current (six occurrences in four studies, 4.4%).

**Fig. 16:**
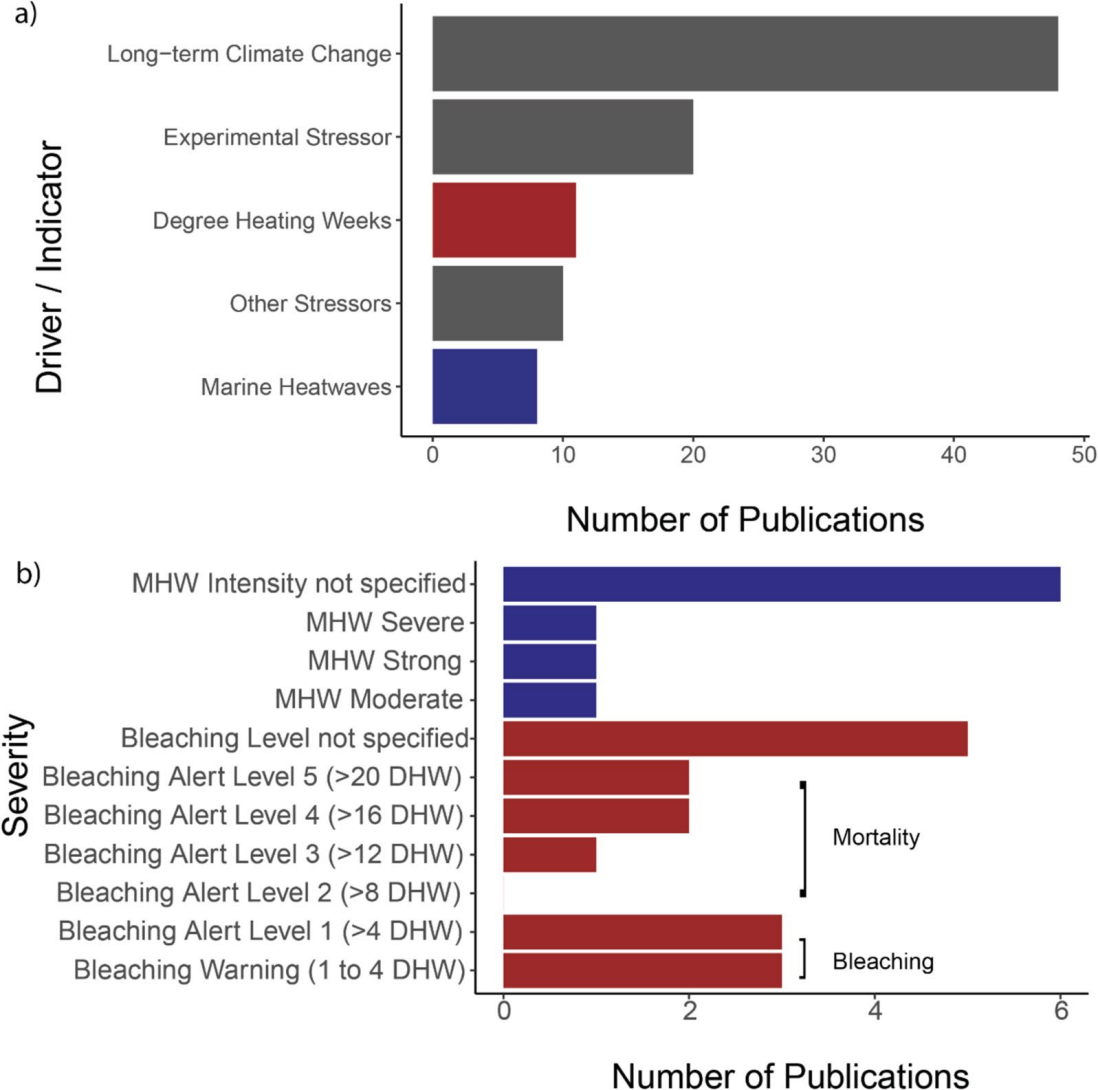
**a** Drivers of extreme weather events recorded, including degree heating weeks (DHW) (Red) and marine heatwaves (MHW) (Blue). **b** Number of publications reporting the severity of DHW bleaching alerts and MHW severity [36]

*Drivers*. Most studies considered long-term climate change as a driver of ocean warming (48 papers, 53.3%). This is followed by use of experimental stressors such as artificially raising temperature and acute thermal stress treatments (20 papers, 22.2%). The other stressors include seasonal variation (four papers, 4.4%), localised hotspots (two papers, 2.2%), cold stress (two papers, 2.2%), El Nino Southern Oscillation (one paper, 1.1%), tidal temperature change (one paper, 1.1%), and temperature difference along latitudes (one paper, 1.1%) (Fig. 16a).

*Indicators.* 17 papers (18.9%) reported degree heating weeks, marine heatwaves, or both as identifiers of the severity of ocean warming (Fig. 16b). Degree heating weeks were the most recorded stressor indicator (11 papers, 12.2%) of ocean warming and marine heatwaves being the second (eight papers, 8.9%).

*Severity*. Six papers did not specify the intensity of marine heatwaves, one paper recorded a moderate and a strong marine heatwave event, and one paper recorded a severe marine heatwave event. For studies reporting degree heating weeks as recording matrix, five papers did not specify the level of bleaching alert. Two separate papers recorded bleaching events exceeding bleaching alert level 5 (> 20 DHW), another two papers recorded bleaching events exceeding bleaching alert level 4 (> 16 DHW). Bleaching alert level 3 (> 12 DHW) was reported in one paper, while bleaching alert level 2 (> 8 DHW) was not reported in the papers. Three bleaching alert level 1 (> 4 DHW) were reported in two separate papers, and three bleaching warnings (1 to 4 DHW) were reported in 1 paper (Fig. 16b).

### Limitation of the map

**Limitations of the mapping process.** While a systematic search strategy was used for this systematic map, it is essential to acknowledge the omission of relevant studies may have occurred. Specifically, we have limited the language of our search results to be in English only. There is literature available in other languages that could not be included in this systematic map due to the limited language backgrounds of our authors. Authors with different linguistic background should be engaged in future mapping efforts to overcome this language barrier [61]. Further, including more specific terms in search strings used for future database searches in English and other languages may assist in capturing a broader spectrum of relevant articles. Additionally, for the purpose of this systematic map, we have limited the literature to publications since 2010 and before June 2023. We acknowledge that literature regarding impact of ocean warming on subtropical and temperate coral reefs and coral systems exist prior to 2010, however, in this systematic map, the focus is the literature produced in the last decade, which is most relevant to the introduction and utilisation of ocean warming indicators on coral reefs, such as degree heating weeks and marine heatwaves.

Grey literature search was limited to Open Access Theses and Dissertation database. Reports from government database or specialist website such as National Oceanic and Atmospheric Administration were not included systematic map. Governmental databases exhibit inconsistencies between regions in terms of content. Additionally, reports on National Oceanic and Atmospheric Administration are technical reports based on data collection technology, while research publications were available in Scopus or Web of Science search. Despite this, we acknowledge that a more extensive grey literature search could benefit this systematic map.

**Limitations of the evidence base.** In this systematic map, the limitations of the evidence base may have affected the results and conclusion of our map. We identified the types of study for this systematic map limited to observational, experimental, and a combination of observational or experimental with modelling. However, in the excluded articles we observed a high number (n = 11) that are solely modelling based. These studies while not included in this systematic map, may contain data that other researchers and management parties that may consider useful to research or decision making. Additionally, the decision to exclude literature with solely interactions between ocean acidification and ocean warming may limit the understanding of variability of ocean warming based on different drivers.

## Conclusions

*Ecoregions, provinces and sources of ocean warming impact evidence.* Interestingly, research efforts for subtropical coral research and subsequently evidence for the impact of ocean warming in these ecosystems has predominantly focused on ecoregions in the Asian Pacific region. Specifically, the provinces of South China Sea, Andaman, Warm Temperate Northwest Pacific, and South Kuroshio have been focal points for research effort (a total of 27 publications). Research in these sites [15, 16] was primarily published on and after 2015, associated with coral bleaching events reported in 2014 in Hong Kong [15, 16], 2016 in the Andaman Sea [62] and 2016 in Japan [17]. Therefore, ecoregions are also identified where there are research gaps and evidence for the impact of ocean warming has remained low, such as Delagoa, Mascarene Islands, Arabian Gulf, Natal, etc. While these ecoregions are directly exposed to the Western Indian Ocean, there is evidence of bleaching events in the area [63]. As such, further research providing evidence for the response of corals and coral reef ecosystems in understudied regions is needed alongside continuing evidence from regions where baseline information is currently available.

Studies where research effort has been associated with recorded marine protected areas were identified in this systematic map, and we find that most literature linked specifically to marine parks and/or marine reserve has been reported from Australia. Only 23.3% (21 out of 90) of the studies identified here are reported as having conducted research in protected or associated with marine protected areas, while 35.6% of studies (32 out of 90) did not clearly indicate if their published studies took place in or within the region of a marine protected area. Given the recent increases in areas designated MPAs or marine parks internationally [60], future research identifying patterns of research effort, knowledge gaps and evidence synthesis for coral reef ecosystems should consider further investigating the sites within marine protected areas to build evidence-base for park management.

*Drivers and Severity of Ocean Warming.* Prior to 2016, most studies considered long-term climate change as an environmental driver for ocean warming, and the research using this as an approach looked to study drivers of ocean warming including coral calcification and adaptation on marginal reefs as a measure of the effect of ocean warming on coral reefs. The introduction of a hierarchical approach developed to defining marine heatwaves through 30 years of temperature record of the regions [36] has now led to the increase in use of marine heatwaves as a tool for identifying potential bleaching events [64, 65]. Use of degree heating weeks for subtropical corals was uncommon until 2017, when an increased number of publications used degree heating week to quantify thermal stress of subtropical coral [57, 66, 67] was observed. The use of marine heatwave and degree heating weeks metrics provide researchers with quantitative metrics for evaluating severity of an ocean warming event and/or bleaching event. A combined use of both marine heatwave and degree heating week as reporting mechanisms may benefit future studies in quantitatively comparing data between sites and events.

*Implications for policy/management.* The collection of evidence regarding impact of ocean warming on subtropical and temperate coral reefs provided in this report is accessible to policymakers and practitioners. The empirical data extracted from this systematic map can serve as a foundation for policymakers, practitioners, institutions, and organisations for locating most relevant evidence and up-to-date evidence for their work. Assessment of potential sites for marine protected areas, effectiveness of marine protected areas, and resource allocation for future conservation in areas beyond close and territorial sea locations can be conducted based on this systematic map.

*Implications for research*. Research globally has primarily focused on reporting ecological responses to climate change associated events; however, substantial effort has also been directed to experimental studies. Physiological data is one of the most common forms of impact measures, and data was reported in the studies included in our map, with 30% of the identified literature reporting on the physiological state of corals associated with climate impacts in subtropical regions. Future research in this area should consider a meta-analysis of the evidence identified in the current study, these include but are not limited to the outcomes or predictions, severity reported, locations of research, drivers of ocean warming, and recorded ocean warming events. Additionally, questions on topics such as effectiveness of marine protected area on conservation, accuracy of using degree heating weeks and/or marine heatwaves in identifying bleaching events, coral reefs studies in understudied areas can be addressed in future research. There was severe bleaching events recorded from 2010 to2023 occurring within the mortality scale of impact (degree heating week of 8 or above). Collating the thermal stress reports across subtropical regions will aid managers in predicting and responding to the impact of bleaching on these reefs. Ongoing systematic assessment of research efforts and evidence will also allow for evidence-based management practices specific to these unique and valuable ecosystems.

### Knowledge cluster

Our map identifies clusters of knowledge that demand further examination by systematic reviews. Responses of coral reefs vary by latitude, ecoregions and by oceanographic properties, therefore, syntheses that is location-specific and oceanography-focused would likely be of highest importance to managers. In this systematic map we highlight the high number of publications that are observational based (n = 44) and experimental based (n = 35). These publications have sufficient evidence for synthesising a review. Researchers could examine the impacts of ocean warming on subtropical coral reefs in ecoregions with sufficient evidence, such as Southern China, Western Mediterranean, East China Sea, and Lord Howe and Norfolk Islands. Efforts to congregate and collate studies in these ecoregions in a systematic review would be appropriate for future research and providing in-depth synthesis of available evidence.

Majority of published research available in this systematic map took place in developed countries (n = 58), followed by developing economies (n = 31). Research could investigate the how research resources are allocated in these territories, and how it affects the research effort into understanding impacts of ocean warming on subtropical coral reefs. Proximity to coast was also recorded in this systematic map, and it provides an opportunity to synthesise reviews based on the proximity of research sites to coastline, given the sufficient evidence available for close/territorial sea (n = 69). Effectiveness of marine protected areas on preservation of subtropical coral reefs could be assessed based on the number of publications available (n = 21).

Drivers of ocean warming are presented in this systematic map, and long-term climate change was identified in a majority of studies as the driver of ocean warming (n = 48), followed by experimental stressors (n = 20). These provide sufficient basis for systematic reviews on the drivers presented in these publications. Additionally, some studies have used degree heating weeks and marine heatwaves as indicators of ocean warming (n = 17), these publications provide basis to synthesis reviews based on the severity of ocean warming events identified.

### Knowledge gaps

This map also identifies several understudied topics that could benefit from additional primary research efforts. Studies that are observational and experimental based (n = 7) and experimental and modelling based (n = 1) constituted a small number of literature presented in this systematic map, which is less than expected. Considering the number of available studies that are solely observational or experimental, the lack of studies in these two minor categories is likely due to a combination of factors. Firstly, from this systematic map we showed that majority of the studies included are physiological studies of corals, making simulations or modelling of future scenarios less applicable to the study theme. Secondly, it is likely that time constraints have an influence on the types of study published. As data collection for coral studies, especially where long-term climate change is identified as the major driver of ocean warming in this map, it strongly implies that these types of studies would take a considerable amount of time to compile, making modelling or simulations on the same study less than probable. Understanding how existing data could predict future scenarios using simulations or modelling will be important for managers as ocean warming continues to increase in frequency and intensity.

We also identified the lack of numbers of studies in various locations, these include but not limited to the province of Western Indian Ocean, additionally, while not identified in this systematic map, the lack of studies on the topic within West Coast of North America revealed a large knowledge gap. The absence of studies in these areas reflect the overall lack of available scientific information on spatial distribution of subtropical coral reefs. It could be contributed to the lack of resources to support holistic research in these regions. Managers of these regions could encourage citizen science as a mean to establish basis of identifying potential subtropical coral reefs in these regions. Subsequent primary research could benefit potential preservation of these regions based on empirical evidence provided by citizen science reports.

Lastly, ocean warming drivers such as seasonal variation, El Nino Southern Oscillations interactions, localised hot spots and cold stress are understudied, but these topics could provide a better understanding of how ocean warming is influenced by these mechanisms. Future studies could focus on these finer spatial and temporal drivers to understand how subtropical coral reefs are affected by ocean warming on a localised scale. Managers from local agencies would benefit from research that reports localised effects of ocean warming by implementing measures that are specific to the regions described in these studies.

Although gaps in the evidence remain, our systematic map demonstrates an increasing and strong evidence base in the research investigating impacts of ocean warming on subtropical reefs around the world. Future research that focuses on knowledge gaps and systematic reviews that focus on identified knowledge cluster will provide an even higher level of understanding to advocate for proactive management strategies for subtropical coral reefs.

## Supplementary Information

Additional file 1.

Additional file 2.

Additional file 3.

Additional file 4.

Additional file 5.

Additional file 6.

Additional file 7.

Additional file 8.

Additional file 9.

Additional file 10.

## Data Availability

The datasets generated and/or analysed during the current study are available in the GitHub repository, https://github.com/MLH95/Ho-et-al-2024_Systematic-Map

## References

[1] Moberg F, Folke C. Ecological goods and services of coral reef ecosystems. Ecol Econ. 1999;29(2):215–33.

[2] Hughes TP, Baird AH, Bellwood DR, Card M, Connolly SR, Folke C, et al. Climate change, human impacts, and the resilience of coral reefs. Science (1979). 2003;301(5635):929–33. 10.1126/science.1085046 12920289

[3] Hoegh-Guldberg O, Mumby PJ, Hooten AJ, Steneck RS, Greenfield P, Gomez E, et al. Coral reefs under rapid climate change and ocean acidification. Science (1979). 2007;318(5857):1737–42. 10.1126/science.1152509 18079392

[4] Jones GP, McCormick MI, Srinivasan M, Eagle JV. Coral decline threatens fish biodiversity in marine reserves. Proc Natl Acad Sci. 2004;101(21):8251–3. 15150414 10.1073/pnas.0401277101 PMC419589

[5] Pandolfi JM, Bradbury RH, Sala E, Hughes TP, Bjorndal KA, Cooke RG, et al. Global trajectories of the long-term decline of coral reef ecosystems. Science (1979). 2003;301(5635):955–8. 10.1126/science.1085706 12920296

[6] Bellwood DR, Hughes TP, Folke C, Nyström M. Confronting the coral reef crisis. Nature. 2004;429(6994):827–33. 15215854 10.1038/nature02691

[7] Burt JA, Al-Khalifa K, Khalaf E, AlShuwaikh B, Abdulwahab A. The continuing decline of coral reefs in Bahrain. Mar Pollut Bull. 2013;72(2):357–63. 22980773 10.1016/j.marpolbul.2012.08.022

[8] Eddy TD, Lam VWY, Reygondeau G, Cisneros-Montemayor AM, Greer K, Palomares MLD, et al. Global decline in capacity of coral reefs to provide ecosystem services. One Earth. 2021;4(9):1278–85.

[9] Hughes TP, Kerry JT, Álvarez-Noriega M, Álvarez-Romero JG, Anderson KD, Baird AH, et al. Global warming and recurrent mass bleaching of corals. Nature. 2017;543(7645):373–7. 28300113 10.1038/nature21707

[10] Bleuel J, Pennino MG, Longo GO. Coral distribution and bleaching vulnerability areas in Southwestern Atlantic under ocean warming. Sci Rep. 2021;11(1):12833. 34172760 10.1038/s41598-021-92202-2 PMC8233347

[11] Brown BE. Coral bleaching: causes and consequences. Coral Reefs. 1997;16:S129–38.

[12] Pandolfi JM, Connolly SR, Marshall DJ, Cohen AL. Projecting coral reef futures under global warming and ocean acidification. Science (1979). 2011;333(6041):418–22. 10.1126/science.1204794 21778392

[13] Hoegh-Guldberg O, Poloczanska ES, Skirving W, Dove S. Coral reef ecosystems under climate change and ocean acidification. Front Mar Sci. 2017;4(MAY). Available from: https://www.scopus.com/inward/record.uri?eid=2-s2.0-85020174680&doi=10.3389%2ffmars.2017.00158&partnerID=40&md5=5493d87c3491b8f0a7f7e74d2425c1e3.

[14] Anthony KRN, Kline DI, Diaz-Pulido G, Dove S, Hoegh-Guldberg O. Ocean acidification causes bleaching and productivity loss in coral reef builders. Proc Natl Acad Sci. 2008;105(45):17442–6. 18988740 10.1073/pnas.0804478105 PMC2580748

[15] Xie JY, Lau DCC, Kei K, Yu VPF, Chow WK, Qiu JW. The 2014 summer coral bleaching event in subtropical Hong Kong. Mar Pollut Bull. 2017;124(2):653–9. 28392092 10.1016/j.marpolbul.2017.03.061

[16] Ip JCH, Zhang Y, Xie JY, Yeung YH, Qiu JW. Stable Symbiodiniaceae composition in three coral species during the 2017 natural bleaching event in subtropical Hong Kong. Mar Pollut Bull. 2022;184: 114224. 36240631 10.1016/j.marpolbul.2022.114224

[17] Nakamura M, Murakami T, Kohno H, Mizutani A, Shimokawa S. Rapid recovery of coral communities from a mass bleaching event in the summer of 2016, observed in Amitori Bay, Iriomote Island, Japan. Mar Biol. 2022;169(8):104. 35915766 10.1007/s00227-022-04091-2 PMC9331011

[18] Moriarty T, Leggat W, Heron SF, Steinberg R, Ainsworth TD. Bleaching, mortality and lengthy recovery on the coral reefs of Lord Howe Island. The 2019 marine heatwave suggests an uncertain future for high-latitude ecosystems. PLOS Clim. 2023;2(4):e0000080.

[19] Samayoa AP, Aguirre JD, Delrieu-Trottin E, Liggins L. The origins of marine fishes endemic to subtropical islands of the Southwest Pacific. J Biogeogr. 2023;50(8):1388–401.

[20] Chong F, Sommer B, Stant G, Verano N, Cant J, Lachs L, et al. High‐latitude marginal reefs support fewer but bigger corals than their tropical counterparts. Ecography. 2023;2023(12).

[21] Hughes TP, Bellwood DR, Connolly SR. Biodiversity hotspots, centres of endemicity, and the conservation of coral reefs. Ecol Lett. 2002;5(6):775–84.

[22] Tkachenko KS, Soong K. Dongsha Atoll: a potential thermal refuge for reef-building corals in the South China Sea. Mar Environ Res. 2017;127:112–25. 28395870 10.1016/j.marenvres.2017.04.003

[23] Beger M, Sommer B, Harrison PL, Smith SDA, Pandolfi JM. Conserving potential coral reef refuges at high latitudes. Divers Distrib. 2014;20(3):245–57.

[24] Chollett I, Mumby P, Cortés J. Upwelling areas do not guarantee refuge for coral reefs in a warming ocean. Mar Ecol Prog Ser. 2010;14(416):47–56.

[25] Yamano H, Sugihara K, Nomura K. Rapid poleward range expansion of tropical reef corals in response to rising sea surface temperatures. Geophys Res Lett. 2011;38(4):n/a-n/a.

[26] Jones LA, Mannion PD, Farnsworth A, Bragg F, Lunt DJ. Climatic and tectonic drivers shaped the tropical distribution of coral reefs. Nat Commun. 2022;13(1):3120. 35701413 10.1038/s41467-022-30793-8 PMC9198051

[27] Lachs L, Sommer B, Cant J, Hodge JM, Malcolm HA, Pandolfi JM, et al. Linking population size structure, heat stress and bleaching responses in a subtropical endemic coral. Coral Reefs. 2021;40(3):777–90.

[28] Purcell SW, Clarke KR, Rushworth K, Dalton SJ. Defining critical habitats of threatened and endemic reef fishes with a multivariate approach. Conserv Biol. 2014;28(6):1688–98. 25302855 10.1111/cobi.12343

[29] Vergés A, Steinberg PD, Hay ME, Poore AGB, Campbell AH, Ballesteros E, et al. The tropicalization of temperate marine ecosystems: climate-mediated changes in herbivory and community phase shifts. Proc Royal Soc B Biol Sci. 2014;281(1789):20140846. 10.1098/rspb.2014.0846 PMC4100510 25009065

[30] Veron JEN. Conservation of biodiversity: a critical time for the hermatypic corals of Japan. Coral Reefs. 1992;11(1):13–21.

[31] Haddaway NR, Bernes C, Jonsson BG, Hedlund K. The benefits of systematic mapping to evidence-based environmental management. Ambio. 2016;45(5):613–20. 26984257 10.1007/s13280-016-0773-x PMC4980318

[32] Spalding MD, Fox HE, Allen GR, Davidson N, Ferdaña ZA, Finlayson M, et al. Marine ecoregions of the world: a bioregionalization of coastal and shelf areas. Bioscience. 2007;57(7):573–83.

[33] Liu G, Strong AE, Skirving W. Remote sensing of sea surface temperatures during 2002 Barrier Reef coral bleaching. EOS Trans Am Geophys Union. 2003;84(15):137–41.

[34] Liu G, Strong A, Skirving W, Arzayus L. Overview of NOAA coral reef watch program’s near-real-time satellite global coral bleaching monitoring activities. In: Proceedings of 10th International Coral Reef Symposium. Okinawa; 2006. p. 1783–93.

[35] Strong AE, Liub G, Skirving W, Eakin CM. NOAA’s coral reef watch program from satellite observations. Ann GIS. 2011;17(2):83–92. Available from: https://www.scopus.com/inward/record.uri?eid=2-s2.0-79960198554&doi=10.1080%2f19475683.2011.576266&partnerID=40&md5=d23ea25e40a9990df7387e8dba721195.

[36] Hobday AJ, Alexander LV, Perkins SE, Smale DA, Straub SC, Oliver ECJ, et al. A hierarchical approach to defining marine heatwaves. Prog Oceanogr. 2016;141:227–38.

[37] Ho M, Lagisz M, Nakagawa S, Perkins-Kirkpatrick S, Sawyers P, Leggat B, et al. What is the evidence for the impact of ocean warming on subtropical and temperate corals and coral reefs? A Systematic Map Protocol. PROCEED Protocol. 2023; 10.1186/s13750-024-00349-y PMC11580339 39568058

[38] Pullin A, Frampton G, Livoreil B, Petrokofsky G. Collaboration for Environmental Evidence. Guidelines and Standards for Evidence synthesis in Environmental Management. 2022.

[39] Haddaway NR, Macura B, Whaley P, Pullin AS. ROSES RepOrting standards for Systematic Evidence Syntheses: pro forma, flow-diagram and descriptive summary of the plan and conduct of environmental systematic reviews and systematic maps. Environ Evid. 2018;7(1):7.

[40] Ouzzani M, Hammady H, Fedorowicz Z, Elmagarmid A. Rayyan—a web and mobile app for systematic reviews. Syst Rev. 2016;5(1):210. 27919275 10.1186/s13643-016-0384-4 PMC5139140

[41] Ross CL, Schoepf V, DeCarlo TM, McCulloch MT. Mechanisms and seasonal drivers of calcification in the temperate coral *Turbinaria reniformis* at its latitudinal limits. Proc Royal Soc B Biol Sci. 1879;2018(285):20180215. 10.1098/rspb.2018.0215 PMC5998090 29794042

[42] Hoegh-Guldberg O, Fine M, Skirving W, Johnstone R, Dove S, Strong A. Coral bleaching following wintry weather. Limnol Oceanogr. 2005;50(1):265–71.

[43] R Core Team. R: A language and environment for statistical computing [Internet]. 2022 [cited 2024 Mar 8]. Available from: https://www.r-project.org/about.html.

[44] RStudio Team. RStudio: Integrated development environment for R [Internet]. 2023 [cited 2024 Mar 8]. Available from: http://www.rstudio.com/.

[45] Mauri M, Elli T, Caviglia G, Uboldi G, Azzi M. RAWGraphs. In: Proceedings of the 12th Biannual Conference on Italian SIGCHI Chapter. New York, NY, USA: ACM; 2017. p. 1–5.

[46] Wickham H, Chang W, Henry L, Pedersen T, Takahashi K, Wilke C, et al. ggplot2: Create Elegant Data Visualisations Using the Grammar of Graphics. [Internet]. 2024 [cited 2024 Mar 8]. Available from: https://cran.r-project.org/web/packages/ggplot2/index.html. Accessed on 08 Mar 2024.

[47] Fellows I. Wordcloud: Word Clouds. 2018.

[48] Wickham H, RStudio. tidyverse: Easily Install and Load the “Tidyverse” [Internet]. 2023 [cited 2024 Mar 8]. Available from: https://cran.r-project.org/web/packages/tidyverse/index.html.

[49] Wickham H, François R, Henry L, Müller K, Vaughan D, Posit Software, et al. dplyr: A Grammar of Data Manipulation [Internet]. 2023 [cited 2024 Mar 8]. Available from: https://cran.r-project.org/web/packages/dplyr/index.html.

[50] Wickham H. reshape2: Flexibly Reshape Data: A Reboot of the Reshape Package [Internet]. 2020 [cited 2024 Mar 8]. Available from: https://cran.r-project.org/web/packages/reshape2/index.html.

[51] Garnier S, Ross N, Rudis B, Sciaini N, Camargo A, Scherer C. viridis: Colorblind-Friendly Color Maps for R [Internet]. 2024 [cited 2024 Mar 8]. Available from: https://cran.r-project.org/web/packages/viridis/index.html.

[52] Neuwrith E. RColorBrewer: ColorBrewer Palettes [Internet]. 2022 [cited 2024 Mar 8]. Available from: https://cran.r-project.org/web/packages/RColorBrewer/index.html.

[53] Cheng J, Schloerke B, Karambelkar B, Xie Y, Wickham H, Russel K, et al. leaflet: Create Interactive Web Maps with the JavaScript “Leaflet” Library. 2024 [cited 2024 Mar 8]; Available from: https://cran.r-project.org/web/packages/leaflet/index.html.

[54] Vaidyanathan R, Xie Y, Allaire J, Cheng J, Sievert C, Ressell K, et al. htmlwidgets: HTML Widgets for R [Internet]. 2023 [cited 2024 Mar 8]. Available from: https://cran.r-project.org/web/packages/htmlwidgets/index.html.

[55] Chang W, Xie Y, Guillem F, Schloerke B, Perriault N. webshot: Take Screenshots of Web Pages [Internet]. 2023 [cited 2024 Mar 8]. Available from: https://cran.r-project.org/web/packages/webshot/index.html.

[56] Feinerer I, Homik K, Artifex Software Inc. tm: Text Mining Package [Internet]. 2024 [cited 2024 Apr 9]. Available from: https://cran.r-project.org/web/packages/tm/index.html.

[57] Le Nohaïc M, Ross CL, Cornwall CE, Comeau S, Lowe R, McCulloch MT, et al. Marine heatwave causes unprecedented regional mass bleaching of thermally resistant corals in northwestern Australia. Sci Rep. 2017;7(1):14999. 29101362 10.1038/s41598-017-14794-y PMC5670227

[58] United Nations. World Economic Situation and Prospects 2024 [Internet]. 2024 [cited 2024 Mar 8]. Available from: https://www.un.org/development/desa/dpad/publication/world-economic-situation-and-prospects-2024/.

[59] Kammoun S, Ben Romdhane Y, Fakhfakh MBS. Effects of economic and political risks on foreign direct investment. In 2020. p. 226–40.

[60] Maestro M, Pérez-Cayeiro ML, Chica-Ruiz JA, Reyes H. Marine protected areas in the 21st century: current situation and trends. Ocean Coast Manag. 2019;171:28–36.

[61] Amano T, Ramírez-Castañeda V, Berdejo-Espinola V, Borokini I, Chowdhury S, Golivets M, et al. The manifold costs of being a non-native English speaker in science. PLoS Biol. 2023;21(7): e3002184. 37463136 10.1371/journal.pbio.3002184 PMC10353817

[62] Chavanich S, Kusdianto H, Kullapanich C, Jandang S, Wongsawaeng D, Ouazzani J, et al. Microbiomes of healthy and bleached corals during a 2016 thermal bleaching event in the Andaman sea of Thailand. Front Mar Sci. 2022;21:9.

[63] Gudka M, Obura D, Mbugua J, Ahamada S, Kloiber U, Holter T. Participatory reporting of the 2016 bleaching event in the Western Indian Ocean. Coral Reefs. 2020;39(1):1–11.

[64] Tuckett CA, de Bettignies T, Fromont J, Wernberg T. Expansion of corals on temperate reefs: direct and indirect effects of marine heatwaves. Coral Reefs. 2017;36(3):947–56.

[65] Giraldo-Ospina A, Kendrick GA, Hovey RK. Depth moderates loss of marine foundation species after an extreme marine heatwave: could deep temperate reefs act as a refuge? Proc Royal Soc B Biol Sci. 1928;2020(287):20200709. 10.1098/rspb.2020.0709 PMC7341917 32517616

[66] Goyen S, Camp EF, Fujise L, Lloyd A, Nitschke MR, LaJeunesse TC, et al. Correction to: mass coral bleaching of *P. versipora* in Sydney Harbour driven by the 2015–2016 heatwave. Coral Reefs. 2019;38(4):877–877.

[67] Kim SW, Sampayo EM, Sommer B, Sims CA, Gómez-Cabrera MC, Dalton SJ, et al. Refugia under threat: mass bleaching of coral assemblages in high-latitude eastern Australia. Glob Chang Biol. 2019;25(11):3918–31. 31472029 10.1111/gcb.14772

