# Supplementary material for: What is the evidence for the impact of ocean warming on subtropical and temperate corals and coral reefs? A systematic map
Source: Environ Evid. 2024 Nov 21;13:25. doi: 10.1186/s13750-024-00349-y (PMC11580339; doi:10.1186/s13750-024-00349-y)
Supplement: Supplementary file 1 — Additional file 1. [file 13750_2024_349_MOESM1_ESM.docx]

**Additional File 10 Coral Species**

**ReadMe**

This file describes the species of Cnidarian included in the studies included in the systematic map by Ho et al. (2024): **What is the evidence for the impact of ocean warming on subtropical and temperate corals and coral reefs? A systematic map.** These species are presented as a table in the next 4 pages.

The first column describes the species taxonomic binomial name.

The second column describes the families of the species.

The third column describes the orders of the species.

The fourth column describes the classes of the species.

The fifth and final column describes the number of times the species appeared in the included research for this systematic map.

Additional File 10 Species of Cnidarian studied in the literature included in this Systematic Map.

| Species | Family | Order | Class | Count |
| --- | --- | --- | --- | --- |
| Siderastrea stellata | Siderastreidae | Scleractinia | Hexacorallia | 2 |
| Siderastrea sp. | Siderastreidae | Scleractinia | Hexacorallia | 1 |
| Acropora sp. | Acroporidae | Scleractinia | Hexacorallia | 9 |
| Montipora sp. | Acroporidae | Scleractinia | Hexacorallia | 6 |
| Acropora hyacinthus | Acroporidae | Scleractinia | Hexacorallia | 4 |
| Acropora solitaryensis | Acroporidae | Scleractinia | Hexacorallia | 3 |
| Alveopora japonica | Acroporidae | Scleractinia | Hexacorallia | 3 |
| Acropora millepora | Acroporidae | Scleractinia | Hexacorallia | 2 |
| Acropora pruinisa | Acroporidae | Scleractinia | Hexacorallia | 2 |
| Alveopora sp. | Acroporidae | Scleractinia | Hexacorallia | 2 |
| Astreopora sp. | Acroporidae | Scleractinia | Hexacorallia | 2 |
| Montipora digitata | Acroporidae | Scleractinia | Hexacorallia | 2 |
| Acropora downingi | Acroporidae | Scleractinia | Hexacorallia | 1 |
| Acropora muricata | Acroporidae | Scleractinia | Hexacorallia | 1 |
| Acropora pulchra | Acroporidae | Scleractinia | Hexacorallia | 1 |
| Acropora samoensis | Acroporidae | Scleractinia | Hexacorallia | 1 |
| Acropora spathulata | Acroporidae | Scleractinia | Hexacorallia | 1 |
| Acropora tumida | Acroporidae | Scleractinia | Hexacorallia | 1 |
| Acropora valida | Acroporidae | Scleractinia | Hexacorallia | 1 |
| Acropora yongei | Acroporidae | Scleractinia | Hexacorallia | 1 |
| Alveopora fenestrata | Acroporidae | Scleractinia | Hexacorallia | 1 |
| Montipora caliculata | Acroporidae | Scleractinia | Hexacorallia | 1 |
| Montipora millepora | Acroporidae | Scleractinia | Hexacorallia | 1 |
| Montipora monasteriata | Acroporidae | Scleractinia | Hexacorallia | 1 |
| Montipora peltiformis | Acroporidae | Scleractinia | Hexacorallia | 1 |
| Anemonia viridis | Actiniidae | Actiniaria | Hexacorallia | 1 |
| Anthopleura ballii | Actiniidae | Actiniaria | Hexacorallia | 1 |
| Pavona sp. | Agariciidae | Scleractinia | Hexacorallia | 3 |
| Pavona decussata | Agariciidae | Scleractinia | Hexacorallia | 2 |
| Agaricia sp. | Agariciidae | Scleractinia | Hexacorallia | 1 |
| Gardineroseris sp. | Agariciidae | Scleractinia | Hexacorallia | 1 |
| Pachyseris rugosa | Agariciidae | Scleractinia | Hexacorallia | 1 |
| Pachyseris sp. | Agariciidae | Scleractinia | Hexacorallia | 1 |
| Pavona divaricata | Agariciidae | Scleractinia | Hexacorallia | 1 |
| Pavona venosa | Agariciidae | Scleractinia | Hexacorallia | 1 |
| Alcyonium acaule | Alcyoniidae | Alcyonacea | Octocorallia | 1 |
| Cladiella sp. | Alcyoniidae | Alcyonacea | Octocorallia | 1 |
| Sarcophyton glaucum | Alcyoniidae | Alcyonacea | Octocorallia | 1 |
| Caryophyllia inornata | Caryophylliidae | Scleractinia | Hexacorallia | 1 |
| Corallium rubrum | Coralliidae | Alcyonacea | Octocorallia | 1 |
| Anomastrea irregularis | Coscinaraeidae | Scleractinia | Hexacorallia | 1 |
| Coscinaraea mcneill | Coscinaraeidae | Scleractinia | Hexacorallia | 1 |
| Coscinaraea marshae | Coscinaraeidae | Scleractinia | Hexacorallia | 1 |
| Coscinaraea sp. | Coscinaraeidae | Scleractinia | Hexacorallia | 1 |
| Turbinaria sp. | Dendrophylliidae | Scleractinia | Hexacorallia | 4 |
| Turbinaria reniformis | Dendrophylliidae | Scleractinia | Hexacorallia | 3 |
| Dendrophyllia cornigera | Dendrophylliidae | Scleractinia | Hexacorallia | 2 |
| Dendrophyllia sp. | Dendrophylliidae | Scleractinia | Hexacorallia | 2 |
| Leptopsammia pruvoti | Dendrophylliidae | Scleractinia | Hexacorallia | 2 |
| Turbinaria mesenterina | Dendrophylliidae | Scleractinia | Hexacorallia | 2 |
| Balanophyllia europaea | Dendrophylliidae | Scleractinia | Hexacorallia | 1 |
| Tubastraea tagusensis | Dendrophylliidae | Scleractinia | Hexacorallia | 1 |
| Galaxea fascicularis | Euphylliidae | Scleractinia | Hexacorallia | 5 |
| Galaxea sp. | Euphylliidae | Scleractinia | Hexacorallia | 2 |
| Gyrosmilia sp. | Euphylliidae | Scleractinia | Hexacorallia | 1 |
| Favia speciosa | Faviidae | Scleractinia | Hexacorallia | 3 |
| Favia sp. | Faviidae | Scleractinia | Hexacorallia | 3 |
| Favia malthaii | Faviidae | Scleractinia | Hexacorallia | 1 |
| Favia pallida | Faviidae | Scleractinia | Hexacorallia | 1 |
| Mussa sp. | Faviidae | Scleractinia | Hexacorallia | 1 |
| Fungia sp. | Fungiidae | Scleractinia | Hexacorallia | 3 |
| Lithophyllon sp. | Fungiidae | Scleractinia | Hexacorallia | 1 |
| Lithophyllon undulatum | Fungiidae | Scleractinia | Hexacorallia | 1 |
| Eunicella singularis | Gorgoniidae | Alcyonacea | Octocorallia | 1 |
| Cladocora caespitosa | incertae sedis | Scleractinia | Hexacorallia | 5 |
| Blastomussa merleti | incertae sedis | Scleractinia | Hexacorallia | 1 |
| Blastomussa sp. | incertae sedis | Scleractinia | Hexacorallia | 1 |
| Leptastrea sp. | Leptastreidae | Scleractinia | Hexacorallia | 1 |
| Acanthastrea sp. | Lobophylliidae | Scleractinia | Hexacorallia | 2 |
| Echinophyllia sp. | Lobophylliidae | Scleractinia | Hexacorallia | 2 |
| Lobophyllia sp. | Lobophylliidae | Scleractinia | Hexacorallia | 2 |
| Acanthastrea echinata | Lobophylliidae | Scleractinia | Hexacorallia | 1 |
| Australophyllia wilsoni | Lobophylliidae | Scleractinia | Hexacorallia | 1 |
| Echinopora sp. | Lobophylliidae | Scleractinia | Hexacorallia | 1 |
| Oxypora sp. | Lobophylliidae | Scleractinia | Hexacorallia | 1 |
| Platygyra carnosa | Merulinidae | Scleractinia | Hexacorallia | 5 |
| Favites sp. | Merulinidae | Scleractinia | Hexacorallia | 4 |
| Cyphastrea sp. | Merulinidae | Scleractinia | Hexacorallia | 3 |
| Goniastrae sp. | Merulinidae | Scleractinia | Hexacorallia | 3 |
| Platygyra acuta | Merulinidae | Scleractinia | Hexacorallia | 3 |
| Platygyra sp. | Merulinidae | Scleractinia | Hexacorallia | 3 |
| Coelastrea aspera | Merulinidae | Scleractinia | Hexacorallia | 2 |
| Favites abdita | Merulinidae | Scleractinia | Hexacorallia | 2 |
| Goniastrea sp. | Merulinidae | Scleractinia | Hexacorallia | 2 |
| Hydnophora exesa | Merulinidae | Scleractinia | Hexacorallia | 2 |
| Hydnophora sp. | Merulinidae | Scleractinia | Hexacorallia | 2 |
| Caulastrea tumida | Merulinidae | Scleractinia | Hexacorallia | 1 |
| Cyphastrea microphthalma | Merulinidae | Scleractinia | Hexacorallia | 1 |
| Dipsastraea | Merulinidae | Scleractinia | Hexacorallia | 1 |
| Dipsastraea sp. | Merulinidae | Scleractinia | Hexacorallia | 1 |
| Favites pentagona | Merulinidae | Scleractinia | Hexacorallia | 1 |
| Goniastrea aspera | Merulinidae | Scleractinia | Hexacorallia | 1 |
| Goniastrea australensis | Merulinidae | Scleractinia | Hexacorallia | 1 |
| Goniastrea favulus | Merulinidae | Scleractinia | Hexacorallia | 1 |
| Leptoria sp. | Merulinidae | Scleractinia | Hexacorallia | 1 |
| Orbicella faveolata | Merulinidae | Scleractinia | Hexacorallia | 1 |
| Oulophyllia sp. | Merulinidae | Scleractinia | Hexacorallia | 1 |
| Paragoniastrea sp. | Merulinidae | Scleractinia | Hexacorallia | 1 |
| Paragoniastrea australensis | Merulinidae | Scleractinia | Hexacorallia | 1 |
| Paramontastraea sp. | Merulinidae | Scleractinia | Hexacorallia | 1 |
| Pectinia sp. | Merulinidae | Scleractinia | Hexacorallia | 1 |
| Platygyra contorta | Merulinidae | Scleractinia | Hexacorallia | 1 |
| Platygyra lamellina | Merulinidae | Scleractinia | Hexacorallia | 1 |
| Montastrea sp. | Montastraeidae | Scleractinia | Hexacorallia | 1 |
| Montastrea cavernosa | Montastraeidae | Scleractinia | Hexacorallia | 1 |
| Mussismilia braziliensis | Mussidae | Scleractinia | Hexacorallia | 1 |
| Mussismilia hispida | Mussidae | Scleractinia | Hexacorallia | 1 |
| Pseudodiploria clivosa | Mussidae | Scleractinia | Hexacorallia | 1 |
| Pseudodiploria strigosa | Mussidae | Scleractinia | Hexacorallia | 1 |
| Oculina arbuscula | Oculinidae | Scleractinia | Hexacorallia | 3 |
| Oculina sp. | Oculinidae | Scleractinia | Hexacorallia | 1 |
| Oulastrea crispata | Oulastreidae | Scleractinia | Hexacorallia | 3 |
| Oulastrea sp. | Oulastreidae | Scleractinia | Hexacorallia | 1 |
| Plesiatrea versipora | Plesiastreidae | Scleractinia | Hexacorallia | 3 |
| Plesiastrea sp. | Plesiastreidae | Scleractinia | Hexacorallia | 2 |
| Paramuricea clavata | Plexauridae | Alcyonacea | Octocorallia | 1 |
| Pocillopora damicornis | Pocilloporidae | Scleractinia | Hexacorallia | 10 |
| Pocillopora sp. | Pocilloporidae | Scleractinia | Hexacorallia | 6 |
| Stylophora pistillata | Pocilloporidae | Scleractinia | Hexacorallia | 3 |
| Stylophora sp. | Pocilloporidae | Scleractinia | Hexacorallia | 3 |
| Pocillopora aliciae | Pocilloporidae | Scleractinia | Hexacorallia | 2 |
| Pocillopora verrucosa | Pocilloporidae | Scleractinia | Hexacorallia | 2 |
| Seriatopora sp. | Pocilloporidae | Scleractinia | Hexacorallia | 2 |
| Porites sp. | Poritidae | Scleractinia | Hexacorallia | 10 |
| Goniopora sp. | Poritidae | Scleractinia | Hexacorallia | 4 |
| Porites heronensis | Poritidae | Scleractinia | Hexacorallia | 3 |
| Porites lutea | Poritidae | Scleractinia | Hexacorallia | 3 |
| Porites lobata | Poritidae | Scleractinia | Hexacorallia | 1 |
| Psammocora sp. | Psammocoridae | Scleractinia | Hexacorallia | 2 |
| Psammocora albopicta | Psammocoridae | Scleractinia | Hexacorallia | 1 |
| Psammocora profundacella | Psammocoridae | Scleractinia | Hexacorallia | 1 |
| Astrangia poculata | Rhizangiidae | Scleractinia | Hexacorallia | 6 |
| Cereus pedonculatus | Sagartiidae | Actiniaria | Hexacorallia | 1 |
| Palythoa clavata | Sphenopidae | Zoantharia | Hexacorallia | 2 |
| Palythoa caribaeorum | Sphenopidae | Zoantharia | Hexacorallia | 2 |
| Palythoa grandiflora | Sphenopidae | Zoantharia | Hexacorallia | 1 |
| Veretillum cynomorium | Veretillidae | Pennatulacea | Octocorallia | 1 |
| Xenia crassa | Xeniidae | Alcyonacea | Octocorallia | 1 |
| Isaurus tuberculatus | Zoanthidae | Zoantharia | Hexacorallia | 1 |
| Zoanthus pulchellus | Zoanthidae | Zoantharia | Hexacorallia | 1 |
