# Supplementary material for: What is the evidence for the impact of ocean warming on subtropical and temperate corals and coral reefs? A systematic map
Source: Environ Evid. 2024 Nov 21;13:25. doi: 10.1186/s13750-024-00349-y (PMC11580339; doi:10.1186/s13750-024-00349-y)
Supplement: Supplementary file 4 — Additional file 4. [file 13750_2024_349_MOESM4_ESM.docx]

**Additional File 3 Development Process**

**ReadMe**

This file describes the process of development of search string used in the work by Ho et al. (2024), **What is the evidence for the impact of ocean warming on subtropical and temperate corals and coral reefs? A systematic map.**

This document includes the following section:

- Articles used for comprehensiveness assessment – This includes the list of articles used as benchmarks.
  - First column describes the title of articles.
  - Second column describes the name of the first author.
  - Third column is the year of publication.
  - Fourth and final column is the Digital Object Identifier of the articles
- Search String Development – This describes the development process of the search string used on Scopus and Web of Science Core Collection.
- Figure A1 shows the keywords described in the ten benchmark articles.

**Articles used for comprehensiveness assessment:**

| Title | First Author | Year | DOI |
| --- | --- | --- | --- |
| Climate and the latitudinal limits of subtropical reef development | Toth, L.T. | 2021 | 10.1038/s41598-021-87883-8 |
| No evidence for tropicalization of coral assemblages in a subtropical climate change hot spot | Mizerek, T.L. | 2021 | 10.1007/s00338-021-02167-x |
| Coral distribution and bleaching vulnerability areas in Southwestern Atlantic under ocean warming | Bleuel, J. | 2021 | 10.1038/s41598-021-92202-2 |
| Low coral mortality during the most intense bleaching event ever recorded in subtropical Southwestern Atlantic reefs | Banha T.N.S. | 2020 | 10.1007/s00338-019-01856-y |
| Successive marine heatwaves cause disproportionate coral bleaching during a fast phase transition from El Nino to La Nina | Dalton, S.J. | 2020 | 10.1016/j.scitotenv.2020.136951 |
| Subtropical thermal variation supports persistence of corals but limits productivity of coral reefs | McIlory, S.E. | 2019 | 10.1098/rspb.2019.0882 |
| What can South African reefs tell us about the future of high-latitude coral systems? | Schleyer, M.H. | 2018 | 10.1016/j.marpolbul.2018.09.014 |
| The 2014 summer coral bleaching event in subtropical Hong Kong | Xie, J.Y. | 2017 | 10.1016/j.marpolbul.2017.03.061 |
| Climate-driven regime shift of a temperate marine ecosystem | Wernberg, T. | 2016 | 10.1126/science.aad8745 |
| Conserving potential coral reef refuges at high latitudes | Beger, M. | 2014 | 10.1111/ddi.12140 |

**Search String Development**

Keywords from the benchmark literature were captured on Scopus, either as author provided keywords or, in the case where authors did not provide keywords, Scopus indexing keywords were used. These were compiled into a word cloud to show the highest occurrence of keywords within these articles in Figure A1. We observed keywords such as “climate”, “change”, “coral”, “reef”, etc. to have a high occurrence. In our systematic map, we decided to replace “climate change” with more specific terms, such as “ocean warming” and “marine heat wave” in our search string, which are also available in the list of keywords identified from the benchmark literature.

We began the search string development using words we defined PECO with and keywords obtained from benchmark literature. These include words such as “marine heatwaves”, “ocean warming”, “coral”, “subtropical”, “temperate”, etc. We aim to find the literature through screening through titles, abstracts and keywords on Scopus. For testing the comprehensiveness of search string, the DOI from the benchmark literature were compiled into a separate search string:

( DOI ( 10.1126/science.aad8745 ) OR DOI ( 10.1016/j.cub.2019.06.077 ) OR DOI ( 10.1111/ddi.12140 ) OR DOI ( 10.1016/j.marpolbul.2018.09.014 ) OR DOI ( 10.1016/j.scitotenv.2020.136951 ) OR DOI ( 10.1038/s41558-019-0412-1 ) OR DOI ( 10.1038/s43017-020-0068-4 ) OR DOI ( 10.1098/rspb.2019.0882 ) OR DOI ( 10.1007/s00338-019-01856-y ) OR DOI ( 10.1038/s41598-021-92202-2 ) OR DOI ( 10.1016/j.marpolbul.2017.03.061 ) OR DOI ( 10.1007/s00338-021-02167-x ) OR DOI ( 10.1038/s41598-021-87883-8 ) )

This separate search string can be used to create a combined query on Scopus search engine to test the comprehensiveness of the database search string to check if it contains all benchmark literature. We began with an initial search string that contains words we used when defining the PECO criteria:

TITLE-ABS-KEY ( ( marine AND heatwave* ) OR mhw* OR ( ocean AND warming ) OR ( ( degree AND heating AND week* ) OR dhw* ) OR ( heat AND stress ) OR stress* )

AND

TITLE-ABS-KEY ( coral* )

AND

TITLE-ABS-KEY ( ( mortality OR mortal* ) OR ( tropicalisation OR tropicalization ) OR shift OR habitat OR increase OR decline OR decrease OR impact OR threat* OR bleach* OR ( acclimatisation OR acclimatization ) )

AND

TITLE-ABS-KEY ( subtropical OR temperate OR ( high AND latitude ) OR marginal OR ( marine AND cold AND ( spell* OR spot ) ) )

This yielded 475 results on Scopus on 14^th^ November, 2022. However, it only captured 5 of the 10 benchmark literature. Next, we attempted to create a search string that emphasised on capturing literature that contain the word “coral” and “subtropical” or “temperate” using the Boolean operator “PRE/n”. Starting with n = 3, 6, 12, we experimented with the search string as follows, where n can be replaced with 3, 6, or 12.

TITLE-ABS-KEY ( ( coral* OR "coral reef*" ) PRE/n ( subtropical OR temperate ) )

AND

TITLE-ABS-KEY ( ( "ocean warming" OR "marine heatwave*" OR "marine heat wave*" OR "mhw" OR "degree heating week*" OR "dhw" OR "heat stress*" OR stress OR tropicali?ation ) )

AND

TITLE-ABS-KEY ( ( mortal* OR tropicali?ation OR shift OR habitat OR increase OR decline OR decrease OR impact OR threat* OR bleach* OR acclimati?ation ) )

For this search string, we have captured 22, 45, and 65 results for n = 3, 6, and 12 respectively. These were accessed on 14^th^ November, 2022. In terms of capturing benchmark literature, these search combinations captured 1, 3, and 3 benchmark literature respectively.

We removed “PRE/n” from the search string and replaced it with a “AND” Boolean operator, as the words subtropical and temperate could occur in front of the terms coral and coral reef. After modifying the search string, we have the following:

TITLE-ABS-KEY ( ( coral* OR "coral reef*" ) AND ( subtropical OR temperate ) )

AND

TITLE-ABS-KEY ( ( "ocean warming" OR "marine heatwave*" OR "marine heat wave*" OR mhw* OR "degree heating week*" OR dhw* OR "heat stress*" OR stress OR tropicali?ation ) )

AND

TITLE-ABS-KEY ( ( mortal* OR tropicali?ation OR shift OR habitat OR increase OR decline OR decrease OR impact OR threat* OR bleach* OR acclimati?ation ) )

This search string yielded 235 results on 15^th^ November, 2022. This search string contained 4 of the benchmark literature. We proceeded to add more terms to the search string as below:

TITLE-ABS-KEY ( ( coral* OR reef* ) AND ( subtropical OR temperate ) )

AND

TITLE-ABS-KEY ( ( "ocean warming" OR "marine heatwave*" OR "marine heat wave*" OR mhw* OR "degree heating week*" OR dhw* OR "heat stress*" OR stress OR tropicali?ation OR temperature* OR "climate change" ) )

AND

TITLE-ABS-KEY ( ( mortal* OR surviv* OR health* OR diseas* OR grow* OR reprodu* OR cover* OR tropicali?at* OR shift* OR habitat* OR increas* OR declin* OR decreas* OR impact* OR threat* OR bleach* OR acclimati?at* ) )

This updated search string yielded 1,067 articles by 10^th^ December, 2022. This search string captured 8 of the benchmark literature.

We separate the “AND (subtropical OR temperate)” from the first “TITLE-ABS-KEY” and created a new “AND” condition, where we can generalise terms and add relevant terms to the string, such as marginal, high latitudes, etc. We developed the final search string below:

TITLE-ABS-KEY ( ( coral* OR “coral reef*” ) )

AND

TITLE-ABS-KEY ( ( “ocean warming” OR “marine heatwave*” OR “marine heat wave*” OR mhw* OR “degree heating week*” OR dhw* OR “heat stress*” OR stress OR tropicali?ation OR temperature* OR “climate change” ) )

AND

TITLEABS-

KEY ( ( mortal* OR surviv* OR health* OR diseas* OR grow* OR reprodu* OR cover* OR tropicali?* OR shift* OR habitat* OR increase* OR declin* OR decreas* OR impact* OR threat* OR bleach* OR acclimati?* OR respons* ) )

AND

TITLE-ABS-KEY ( ( marginal OR “high latitude” OR temperate OR subtropic* OR extratropic* ) )

This search string returned 1,129 results on 10^th^ January, 2023.This search string successfully captured all benchmark articles. This search string was again, accessed on 15^th^ June, 2023, where results are limited to publication since 2010. The search string finally yielded 877 results on Scopus. A search string was translated to Web of Science Core Collection format to search on the database as below:

(All Fields) ( ( coral* OR “coral reef*” ) )

AND

(All Fields) ( ( “ocean warming” OR “marine heatwave*” OR “marine heat wave*” OR mhw* OR “degree heating week*” OR dhw* OR “heat stress*” OR stress OR tropicali?ation OR temperature* OR “climate change” ) )

AND

(All Fields) ( ( mortal* OR surviv* OR health* OR diseas* OR grow* OR reprodu* OR cover* OR tropicali?* OR shift* OR habitat* OR increase* OR declin* OR decreas* OR impact* OR threat* OR bleach* OR acclimati?* OR respons* ) )

AND

(All Fields) ( ( marginal OR “high latitude” OR temperate OR subtropic* OR extratropic* ) )

Which yielded 1,042 articles after filtering out publication prior to 2010.


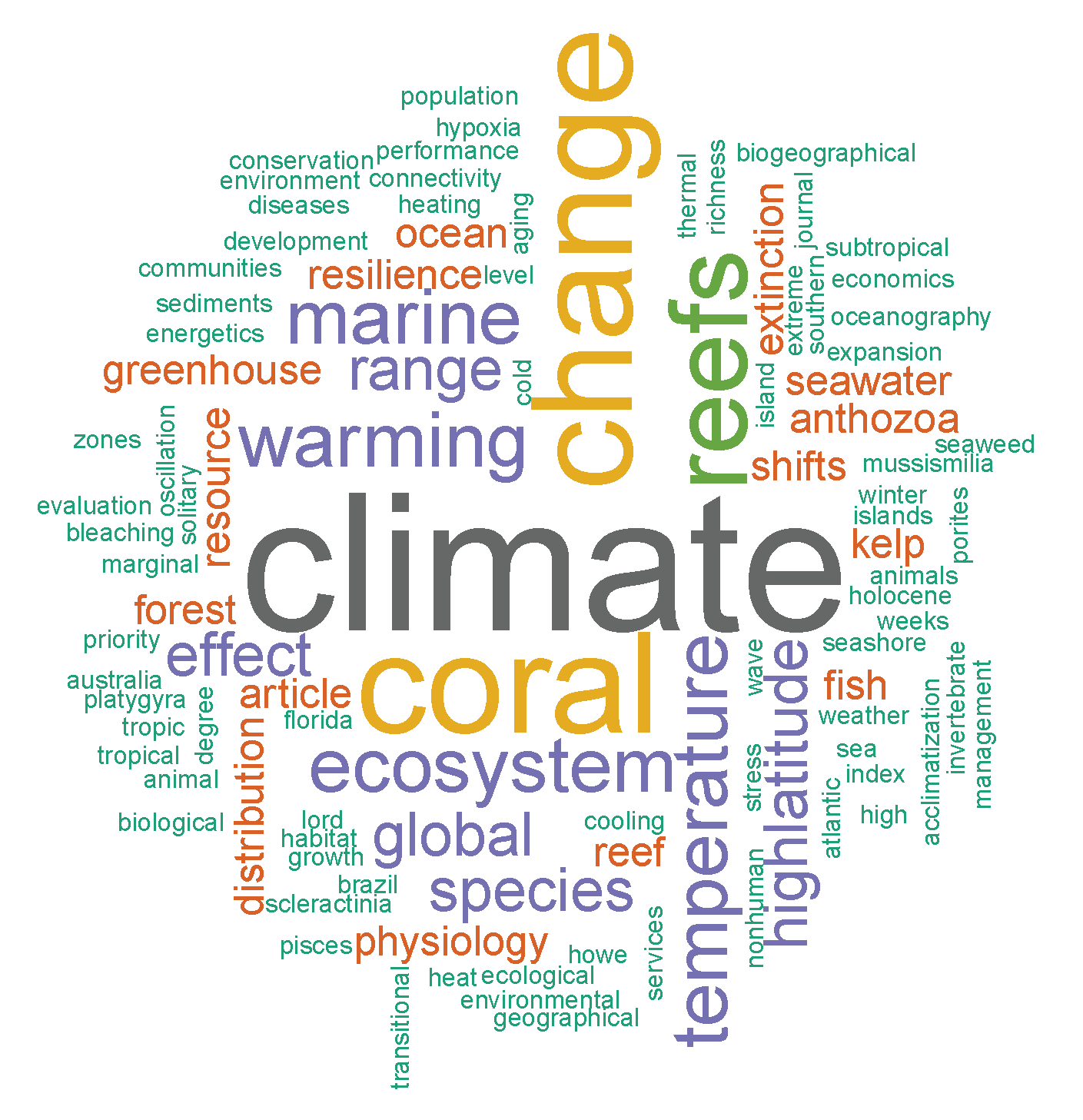

Figure A1. A word cloud showing the highest occurrence of keywords within the benchmark literature.
