# Supplementary material for: What is the evidence for the impact of ocean warming on subtropical and temperate corals and coral reefs? A systematic map
Source: Environ Evid. 2024 Nov 21;13:25. doi: 10.1186/s13750-024-00349-y (PMC11580339; doi:10.1186/s13750-024-00349-y)
Supplement: Supplementary file 5 — Additional file 5. [file 13750_2024_349_MOESM5_ESM.docx]

**Additional File 4 List of Marine Ecoregions of the World**

**ReadMe**

This file is a part of the additional files in the systematic map by Ho et al. (2024): **What is the evidence for the impact of ocean warming on subtropical and temperate corals and coral reefs? A systematic map.**

This file lists the Marine Ecoregions of the World as described by Spalding et al. in their publication in 2007.

The Ecoregions are divided by the type of **climate (tropical, subtropical, temperate and polar)** defined by Beger et al. (2014).

Under the climate heading, Ecoregions are further divided by **Provinces (Bolded texts)**.

Additional File 4 List of Marine Ecoregions of the World (Spalding et al., 2007).

| **Tropical:** |
| --- |
| **Central Polynesia:**  -Line Islands  -Phoneix/Tokelau/Northern Cook Islands  -Samoa Islands  **Southeast Polynesia:**  -Tuamotus  -Rapa-Pitcairn  -Southern Cook/Austral Islands  -Society Islands  **Marquesas:**  Marquesas  **Easter Island:**  -Northern Easter Island  **Galapagos Islands:**  -Northern Galapagos Islands  **Tropical East Pacific:**  -Clipperton  -Chiapas-Nicaragua  -Nicoya  -Cocos Islands  -Panama Bight  **Warm Temperate Northwest Atlantic:**  -Northern Gulf of Mexico  **Tropical Northwestern Atlantic:**  -Bahamian  -Eastern Caribbean  -Greater Antilles  -Southern Caribbean  -Southwestern Caribbean  -Western Caribbean  -Southern Gulf of Mexico  **North Brazil Shelf:**  -Guianan  -Amazonia  **Tropical Southwestern Atlantic:**  -Sao Pedro and Sao Paulo Islands  -Fernando de Naronha and Atoll das Rocas  -Northeastern Brazil  -Eastern Brazil  -Trindade and Martin Vaz Islands  **St. Helena and Ascension Islands:**  -St. Helena and Ascension Islands  **Gulf of Guinea:**  -Gulf of Guinea West  -Gulf of Guinea Upwelling  -Gulf of Guinea Central  -Gulf of Guinea Islands  -Gulf of Guinea South  **Western Indian Ocean:**  -Northern Monsoon Current Coast  -East African Coral Coast  -Seychelles  -Cargados Carajos/Tromelin Island  -Mascarene Islands  -Southeast Madagascar  -Western and Northern Madagascar  -Bight of Sofala/Swamp Coast  **Red Sea and Gulf of Aden:**  -Northern and Central Red Sea  -Southern Red Sea  -Gulf of Aden  **Somali/Arabian:**  -Gulf of Oman  -Western Arabian Sea  -Central Somali Coast  **West and South Indian Shelf:**  -Western India  -South India and Sri Lanka  **Central Indian Ocean Islands:**  -Maldives  -Chagos  **Bay of Bengal:**  -Eastern India  -Northern Bay of Bengal  **Andaman:**  -Andaman and Nicobar Islands  -Andaman Sea Coral Coast  -Western Sumatra  **South China Sea:**  -Gulf of Tonkin  -Southern China  -South China Sea Oceanic Islands  **Sundra Shelf:**  -Gulf of Thailand  -Southern Vietnam  -Sunda Shelf/Java Sea  -Malacca Strait  **Java Transitional:**  -Southern Java  -Cocos-Keeling/Christmas Island  **South Kuroshio:**  -South Kuroshio  **Tropical Northwestern Pacific:**  -Ogasawara Islands  -Mariana Islands  -East Caroline Islands  -West Caroline Islands  **Western Coral Triangle:**  -Palawan/North Borneo  -Eastern Philippines  -Sulawesi Sea/Makassar Strait  -Halmahera  -Papua  -Banda Sea  -Lesser Sunda  -Northeast Sulawesi  **Eastern Coral Triangle:**  -Bismarck Sea  -Solomon Archipelago  -Solomon Sea  -Southeast Papua New Guinea  **Sahul Shelf:**  -Gulf of Papua  -Arafura Sea  -Arnhem Coast to Gulf of Carpenteria  -Bonaparte Coast  **Northeast Australian Shelf:**  -Torres Strait Northern Great Barrier Reef  -Central and Southern Great Barrier Reef  **Northwest Australian Shelf:**  -Exmouth to Broome  -Ningaloo  **Tropical Southwestern Pacific:**  -Tonga Islands  -Fiji Islands  -Vanuatu  -New Caledonia  -Coral Sea  **Hawaii:**  -Hawaii  **Marshall, Gilbert, and Ellis Islands:**  -Marshall Islands  -Gilbert/Ellis Island |
| **Subtropical:** |
| **Northern New Zealand:**  -Kermadec Island  -Three Kings-North Cape  **Easter Island:**  -Southern Easter Island  **Galapagos Islands:**  -Eastern Galapagos Islands  -Western Galapagos Islands  **Tropical East Pacific:**  -Revillagigedos  -Guayaquil  **Tropical Northwestern Atlantic:**  -Gulf of California  -Burmuda  -Floridian  **West African Transition:**  -Cape Verde  -Sahelian Upwelling  **Angulhas:**  -Natal  **Western Indian Ocean:**  -Delagoa  **Somali/Arabian:**  -Arabian (Persian) Gulf  **Lord Howe and Norfolk Islands:**  -Lord Howe and Norfolk Islands  **East Central Australian Shelf:**  -Tweed-Moreton  -Manning-Hawkesbury  **West Central Australian Shelf:**  -Shark Bay  -Houtman  **Warm Temperate Northwest Pacific:**  -Central Kuroshio Current  -East China Sea |
| **Temperate:** |
| **Cold Temperate Northeast Pacific:**  -Aleutian Islands  -Gulf of Alaska  -North American Pacific Fijordland  -Puget Trough/Georgia Basin  -Oregon, Washington, Vancouver Coast and Shelf  -Northern California  **Warm Temperate Northeast Pacific:**  -Baja California  -Baja California Sur  **Warm Temperate Southeastern Pacific:**  -Central Peru  -Humboldtian  -Central Chile  -Araucanian  **Juan Fernandez and Desventuradas:**  -Juan Fernandez and Desventuradas  **Warm Temperate Southwestern Atlantic:**  -Rio Grande do Sul  -Rio de la Plata  -Uruguay-Buenos Aires Shelf  **Magellanic:**  -North Patagonian Gulfs  -Paragonian Shelf  -Malvinas/Falklands  -Channels and Fjords of Southern Chile  -Chiloense  **Warm Temperate Northwest Atlantic:**  -Carolinian  **Tristan Gough:**  -Tristan Gough  **Northern European Seas:**  -South and West Iceland  -Faroe Plateau  -Southern Norway  -Northern Norway and Finnmark  -Baltic Sea  -North Sea  -Celtic Seas  **Lusitanian:**  -South European Atlantic Shelf  -Saharan Upwelling  -Azores Canaries Madeira  **Mediterranean Sea:**  -Adriatic Sea  -Aegean Sea  -Levantine Sea  -Tunisian Plateau/Gulf of Sidra  -Ionian Sea  -Western Mediterranean  -Alboran Sea  **Black Sea:**  -Black Sea  **Gulf of Guinea:**  -Angolan  **Benguela:**  -Namib  -Namaqua  **Agulhas:**  -Aguihas Bank  **Northern New Zealand:**  -Northeastern New Zealand  **Southern New Zealand:**  -Chatham Island  -Central New Zealand  -South New Zealand  -Snares Island  **Southeast Australian Shelf:**  -Cape Howe  -Bassian  -Western Bassian  **Southwest Australian Shelf:**  -South Australian Gulfs  -Great Australian Bight  -Leeuwin  **Cold Temperate Northwest Pacific:**  -Sea of Okhotsk  -Kamchatka Shelf and Coast  -Oyashio Current  -Northeastern Honshu  -Sea of Japan  -Yellow Sea  **Amsterdam-St Paul:**  -Amsterdam-St Paul |
| **Polar:** |
| **Arctic:**  -North Greenland  -North and East Iceland  -East Greenland Shelf  -West Greenland Shelf  -Northern Grand Banks-Southern Labrador  -Northern Labrador  -Baffin Bay-Davis Strait  -Hudson Complex  -Lancaster Sound  -High Arctic Archipelago  -Beaufort-Amundsen-viscount Melville-Queen Maud  -Beaufort Sea-continental coast and shelf  -Chukchi Sea  -Eastern Bering Sea  -East Siberian Sea  -Laptev Sea  -Kara Sea  -North and East Barents Sea  -White Sea  **Subantarctic Islands:**  -Macquarie Island  -Heard and Macdonald Islands  -Kerguelen Islands  -Crozet Islands  -Prince Edward Islands  -Bouvet Island  -Peter the First Island  **Scotia Sea:**  -South Sandwich Islands  -South Georgia  -South Orkney Islands  -South Shetland Islands  -Antarctic Peninsula  **Continental High Antarctic:**  -East Antarctic Wilkes Land  -East Antarctic Enderby Land  -East Antarctic Dronning Maud Land  -Weddell Sea  -Amundsen/Bellingshausen Sea  -Ross Sea  **Subantarctic New Zealand:**  -Bounty and Antipodes Islands  -Campbell Island  -Auckland Island |

Reference:

Spalding MD, Fox HE, Allen GR, Davidson N, Ferdaña ZA, Finlayson M, et al. Marine Ecoregions of the World: A Bioregionalization of Coastal and Shelf Areas. Bioscience. 2007 Jul 1;57(7):573–83.

Beger M, Sommer B, Harrison PL, Smith SDA, Pandolfi JM. Conserving potential coral reef refuges at high latitudes. Divers Distrib. 2014 Mar 24;20(3):245–57.
