# Supplementary material for: What is the evidence for the impact of ocean warming on subtropical and temperate corals and coral reefs? A systematic map
Source: Environ Evid. 2024 Nov 21;13:25. doi: 10.1186/s13750-024-00349-y (PMC11580339; doi:10.1186/s13750-024-00349-y)
Supplement: Supplementary file 6 — Additional file 6. [file 13750_2024_349_MOESM6_ESM.docx]

**Additional File 5 Google Form Questionnaire**

**ReadMe**

This file is part of the additional files for the systematic map by Ho et al. (2024): **What is the evidence for the impact of ocean warming on subtropical and temperate corals and coral reefs? A systematic map.**

This additional file describes all the questions included in the Google form questionnaire used for data extraction.

**Bold texts** indicate the questions.

Texts following the question behind a hyphen (“-“) indicate the type of answer to be answered in the field. In the case of checkboxes and multiple choices survey questions, lists of options are shown following the sentence.

***Bold, underlined and italicised text*** are section dividers used in the questionnaire to break down the questionnaire into logical sections.

The Google form can be accessed using the following URL:

<https://docs.google.com/forms/d/1nv_TxvgPnTJ3_IivqHkL_LOrWmO1V1DcuW3rBlXesIg/>

The following is the questions from the Google form.

Data Extraction Form Version 3 (Oct 2023)

Please use comma (,) as a separator so data collection on excel will be tidier

**Title of Article** – Short answer text

**First Author, Last Author (Last name only, e.g. Doe, Doe)** – Short answer text

**Corresponding or First Author Location (Institution and country, e.g. University of New South Wales, Australia)** – Long answer text

**Year of Publication (YYYY)** – Short answer text

**DOI (10.XXXX/xxxxxx)** – Short answer text

**Reason for exclusion** – Checkboxes

- N/A - Study included for data extraction
- Full text unavailable
- Full text is in a foreign language beyond the language scope of reviewers
- Research location falls outside of subtropical/temperate regions
- Does not involve a warming event
- Not primary research
- Only modelling, no observation/experiment in study
- Article published before 2010
- Other

**Comments on Exclusion (If article is excluded, no further questions are needed to be answered.)** – Short answer text

**Is the article included for screening?** – Yes/No

***Study Classification***

**Study Type** – Checkboxes

- Experimental
- Observational
- Modelling/Simulation

**Comment on Study Type** – Short answer text

**Keywords used in this literature (Please use comma to separate keywords)** – Short answer text

**Location of Study (Location Name only)** – Short answer text

**Coastal/Offshore/Island?** – Checkboxes

- Coastal
- Offshore (Shelf Water)
- Island

**Is it in proximity to a exclusive economic zone that is a tourist attraction or urbanisation region?** – Yes/No

**If yes, please specify the area:** - Short answer text

**What is the proximity of the site in relation to the mentioned area? (United Nations Conference on the Law of the Sea, 1982). Select multiple if applicable.** – Checkboxes

- Close/Territorial Sea (0-12 nautical miles/22.22km/13.81mil)
- Middle/Contiguous Zone (12-24 nautical miles/22.22-44.45km/13.81-230.16mil)
- Far/Exclusive Economic Zone (24-200 nautical miles/44.45-370.4km/27.62-230.16mil)
- Not specified

**Approximate Latitude, Longitude, minimum 2 decimal places (e.g. 22.3193, 114.1694. For Southern hemisphere, use -ve sign to indicate, N/A if not applicable)** – Short answer text

Development Status of Country (Use UN definition: https://www.un.org/en/development/desa/policy/wesp/wesp_current/2014wesp_country_classification.pdf ) - Checkboxes

- Developed Economies
- Economies in Transition
- Developing Economies
- Fuel-exporting Countries
- Not applicable

**Did the study take place at a protected area (E.g. marine park, etc.)?** – Yes/No

**If the study took place at a protected area, please specify:** - Short answer text

**Did the author refer to the event taking place in an ocean current or other water bodies?** – Checkboxes

- Ocean Currents
- Open water
- Lagoon
- Estuary
- Other

***Study Timeframe***

**When did the event start? (e.g. Sep 2022)** – Short answer text

**When did the event end? (e.g. Feb 2023)** – Short answer text

**When did the study start? (e.g. Sep 2022)** – Short answer text

**When did the study end? (e.g. Feb 2023)** – Short answer text

**Did the study take place at the same time as the event?** – Yes/No/NA

***Data Collection in Study***

**Temperature data categorisation** – Checkboxes

- Satellite-derived Data
- In-situ Collected/In-situ Derived Data
- Other

**If in-situ, from what depth was the data taken? N/A if satellite-derived or did not specify in paper. (Unit in metres, e.g. 10)** – Short answer text

**If in-situ, which data source is it from? (E.g. IMOS, etc.)? N/A if not specified in paper.** – Short answer text

**Are the temperature raw data available?** – Yes/No

**What organisms are studied?** – Checkboxes

- Coral
- Kelp
- Plankton
- Benthic Species
- Macroalgae
- Fish
- Turf
- Other

**What species are studied? If more than one species, please separate each specie name by coma (,)** – Short answer text

**What are other environmental variables recorded? (e.g. Chlorophyll a concentration, etc.)** – Long answer text

***Summary of Study***

**Is there an outcome/prediction provided in the study?** – Checkboxes

- Outcome
- Prediction
- Other

**If an outcome is provided, please provide a short description of the outcome** – Long answer text

**If a prediction is provided, please provide a short description of the prediction** – Long answer text

**Is the studied site classified as phase shifted/tropicalised/refugia/degraded, etc.?** – Checkboxes

- Phase shift
- Tropicalisation
- Refugia
- Degraded
- NA
- Other

**Is there a causality in the study?** – Yes/No

**Stressor/Indicator used** – Checkboxes

- Long-term climate change
- Marine heat waves
- Degree heating weeks
- Localised hotspot
- Other

**Severity of event based on DHWs/MHWs definition** – Short answer text

**Main Finding of this publication** – Long answer text

**Remarks** – Long answer text
