# Supplementary material for: What is the evidence for the impact of ocean warming on subtropical and temperate corals and coral reefs? A systematic map
Source: Environ Evid. 2024 Nov 21;13:25. doi: 10.1186/s13750-024-00349-y (PMC11580339; doi:10.1186/s13750-024-00349-y)
Supplement: Supplementary file 8 — Additional file 8. [file 13750_2024_349_MOESM8_ESM.docx]

**Additional File 7 Meta Data**

**Read Me**

This document contains the meta data for the extracted data described in Additional File 6 Main Database for the systematic map: **What is the evidence for the impact of ocean warming on subtropical and temperate corals and coral reefs? A systematic map.**

The database can be found as “Additional File 6 Main Database”.

In the table below, the first column describes the headers of the data columns in the database, while the second column is the metadata for the data collected for the header.

**Bolded texts** in the first column describe the name of Worksheet.

*Underlined and italicised texts* are data columns that are frozen on all sheets for easy cross referencing between sheets.

Additional File 7 Meta data for Additional File 6 Main Database.

|  | **Data Description** |
| --- | --- |
| **Bibliographic Data** |  |
| *Title of Article* | The title of the literature collected. |
| *First Author* | The first author's name. |
| *DOI (10.XXXX/xxxxxx)* | The Digital Object Identifier of the literature. |
| Number of Citations | Number of citations as of February 2024. |
| Last Author | The last author's name. |
| Number of Collaborators | Number of authors collaborating on the literature. |
| Corresponding or First Author Location (Institution and country, e.g. University of New South Wales, Australia) | First author's location (Institute location). |
| Faculty Location | Country of first author's location. |
| Year of Publication (YYYY) | The year of publication. |
| Journal | Journal which the literature was published in. |
| Study Type | Study types generalised into observational, experimental and modelling/simulation based on the nature of the literature. |
| Comment on Study Type | Additional comments about the study type. |
| Keywords used in this literature (Please use comma to separate keywords) | Keywords extracted from the literature, based on Scopus or Web of Science Core Collection database. |
| **Location of Research Data** |  |
| EcoRegion | Actual research location, this include field study, lab study, and computational study. |
| Province | Province is where Ecoregion nests within, see Spalding et al. (2007) definition of marine ecoregions of the world for specific classification of province and ecoregion. |
| Coastal/Offshore/Island? | This field is based on the nature of where the research location is, and is limited to field study, and if collection of samples is appropriate. |
| Is it in proximity to a exclusive economic zone that is a tourist attraction or urbanisation region? | This is determined if the research location is within a exclusive economic zone. |
| If yes, please specify the area: | This is the name of which country/territory (countries/territories) are involved in the previous question. |
| What is the proximity of the site in relation to the mentioned area? (United Nations Conference on the Law of the Sea, 1982). Select multiple if applicable. | This is where the research location is within the previously described EEZ. |
| Approximate Latitude, Longitude, minimum 2 decimal places (e.g. 22.3193, 114.1694. For Southern hemisphere, use -ve sign to indicate, N/A if not applicable) | The latitude and longtitude of study sites. |
| Development Status of Country (Use UN definition: https://www.un.org/en/development/desa/policy/wesp/wesp_current/2014wesp_country_classification.pdf ) | This is determined by where the research country/territory is located. United Nations provided a definition for all countries and territories in the world as to which category they fall into. |
| Did the study take place at a protected area (E.g. marine park, etc.)? | If the study area is within a protected location, such as marine park, santuary or equivalent. |
| If the study took place at a protected area, please specify: | Name of previously mentioned location. |
| Did the author refer to the event taking place in an ocean current or other water bodies? | The research location can take place in ocean currents, open water, lagoon, or other water bodies. |
| What organisms are studied? | Species studied, in a common name level (e.g. coral, anemone, macroalgae, etc.) |
| What species are studied? If more than one species, please separate each specie name by coma (,) | Species studied, in a taxonomical name level (e.g. Pocillopora damicornis, Porites lutea, etc.) |
| **Research Data** |  |
| Temperature data categorisation | This explains how the temperature data was collected, for example satellite derived, in-situ measured, etc. |
| If in-situ, from what depth was the data taken? N/A if satellite-derived or did not specify in paper. (Unit in metres, e.g. 10) | If the study provides a depth where oceanographic data (temperature) was collected. |
| If in-situ, which data source is it from? (E.g. IMOS, etc.)? N/A if not specified in paper. | This includes the equipment used, or source used, such as integrated marine observing system. |
| Logging Equipment | This is the classification of data source previously answered. |
| Is there an outcome/prediction provided in the study? | This determines if the literature provided a outcome, prediction, or both as a conclusion. |
| If an outcome is provided, please provide a short description of the outcome | Self-explanatary |
| If a prediction is provided, please provide a short description of the prediction | Self-explanatary |
| Category of results | This is a checkbox question where categories of studies are classified, including benthic interaction, adaptation to marginal reefs, physiological study, etc. |
| Is the studied site classified as phase shifted/tropicalised/refugia/degraded, etc.? | If the literature mentioned the sites' condition that fell into the categories mentioned in the question. |
| Is there a causality in the study? | Self-explanatary |
| What are other environmental variables recorded? (e.g. Chlorophyll a concentration, etc.) | This is where other environmental variables studied in the literature can be inputed. |
| Main Finding of this publication | Self-explanatary |
| Remarks | Self-explanatary |
| Are the temperature raw data available? | Self-explanatary |
| **Ocean Warming Evidence Data** |  |
| When did the event start? (e.g. Sep 2022) | If the study includes an ocean warming event, when did the event start based on their definition of the event? |
| When did the event end? (e.g. Feb 2023) | If the study includes an ocean warming event, when did the event end based on their definition of the event? |
| When did the study start? (e.g. Sep 2022) | Self-explanatary |
| When did the study end? (e.g. Feb 2023) | Self-explanatary |
| Did the study take place at the same time as the event? | Is the study taking place during, overlap or after the previously described event? If the study did not describe an ocean warming event, the answer to this question would be "no". |
| Stressor used | What stressors were used when describing the ocean warming event/study? |
| Severity of event based on DHWs/MHWs definition | Self-explanatary |
