# Supplementary material for: What is the evidence for the impact of ocean warming on subtropical and temperate corals and coral reefs? A systematic map
Source: Environ Evid. 2024 Nov 21;13:25. doi: 10.1186/s13750-024-00349-y (PMC11580339; doi:10.1186/s13750-024-00349-y)
Supplement: Supplementary file 9 — Additional file 9. [file 13750_2024_349_MOESM9_ESM.docx]

**Additional File 8 Author and Number of Publications**

**ReadMe**

This file contains the list of authors and number of publications they are involved in as described in the systematic map by Ho et al. (2024): **What is the evidence for the impact of ocean warming on subtropical and temperate corals and coral reefs? A systematic map.**

The first column describes the name of the last author.

The second column describes the number of publications they are involved in as last author.

Additional File 8 List of Authors and the number of publications they are involved in.

| Author Name | Number of Publication Involved |
| --- | --- |
| Ang, Put | 7 |
| Ross, Claire | 5 |
| Chui, Apple Pui Yi | 4 |
| Huang, Xueyong | 4 |
| Linares, Cristina | 4 |
| McCulloch, Malcolm | 4 |
| Qiu, Jian-Wen | 4 |
| Tsang, Ryan Ho Leung | 4 |
| Yu, Kefu | 4 |
| Agostini, Sylvain | 3 |
| Beger, Maria | 3 |
| Byrne, Maria | 3 |
| Cebrian, Emma | 3 |
| Chen, Chaolun Allen | 3 |
| Dalton, Steven | 3 |
| Davies, Sarah | 3 |
| Davy, Simon | 3 |
| Denis, Vianney | 3 |
| Ferrier-Pages, Christine | 3 |
| Figueira, Will | 3 |
| Garrabou, Joaquim | 3 |
| Goffredo, Stefano | 3 |
| Leggat, William | 3 |
| Qin, Zhenjun | 3 |
| Schoepf, Verena | 3 |
| Sommer, Brigette | 3 |
| Aichelman, Hannah | 2 |
| Ainsworth, Tracy | 2 |
| Airi, Valentina | 2 |
| Barrett, Neville | 2 |
| Baird, Andrew | 2 |
| Brito, Alberto | 2 |
| Cant, James | 2 |
| Carroll, Andrew | 2 |
| Cerrano, Carlo | 2 |
| Chan, Leo | 2 |
| Chen, Biao | 2 |
| Clemente, Sabrina | 2 |
| Day, Paul | 2 |
| De Palmas, Stephane | 2 |
| Dellisanti, Walter | 2 |
| Dove, Sophie | 2 |
| Dubinsky, Zvy | 2 |
| Falini, Giuseppe | 2 |
| Fisher, Paul | 2 |
| French, Ben | 2 |
| Hoegh-Guldberg, Ove | 2 |
| Hovey, Renae | 2 |
| Huang, Wen | 2 |
| Hwang, Sung-Jin Jin | 2 |
| Krueger, Thomas | 2 |
| Kruži?, Peter | 2 |
| Liang, Jiayuan | 2 |
| López, Cataixa | 2 |
| López-Sendino, Paula | 2 |
| Loubeyres, Mathilde | 2 |
| Malcolm, Hamish | 2 |
| Marchini, Chiara | 2 |
| McIlroy, Shelby | 2 |
| Nakamura, Takashi | 2 |
| Pandolfi, John | 2 |
| Pontasch, Stefanie | 2 |
| Qian, Pei-Yuan | 2 |
| Reimer, James | 2 |
| Ribas-Deulofeu, Lauriane | 2 |
| Rotjan, Randi | 2 |
| Sampayo, Eugenia | 2 |
| Song, Jun-Im | 2 |
| Suggett, David | 2 |
| Teixidó, Núria | 2 |
| Wang, Guanghua | 2 |
| Wells, Mark | 2 |
| Woo, Seonock | 2 |
| Wu, Jiajun | 2 |
| Xie, James | 2 |
| Yamano, Hiroya | 2 |
| Yeung, Yip Hung | 2 |
| Aizawa, Hiroaki | 1 |
| Alfaro-Lucas, Joan | 1 |
| Almanzar, Adeline | 1 |
| Angeletti, Lorenzo | 1 |
| Aurelle, Didier | 1 |
| Baker, David | 1 |
| Baker, Susan | 1 |
| Baldi, Veronica | 1 |
| Bally, Marc | 1 |
| Banha, Thomás | 1 |
| Barbosa, Andreia | 1 |
| Barshis, Daniel | 1 |
| Bas-Silvestre, Maria | 1 |
| Bates, Amanda | 1 |
| Bauman, Andrew | 1 |
| Baumann, Justin | 1 |
| Beal, Jeff | 1 |
| Beals, Morgan | 1 |
| Bednarek, Teresa | 1 |
| Bencons, Brooke | 1 |
| Bensoussan, Nathaniel | 1 |
| Béraud, Eric | 1 |
| Bergman, Jessica | 1 |
| Besemer, Nicole | 1 |
| Bessell-Browne, Pia | 1 |
| Bonebrake, Timothy | 1 |
| Booth, David | 1 |
| Braga, Marcus | 1 |
| Brandt, Angelika | 1 |
| Brennan, Sara | 1 |
| Bridge, Tom | 1 |
| Brock, Danny | 1 |
| Bryson, Mitch | 1 |
| Burmester, Elizabeth | 1 |
| Burns, Andrew | 1 |
| Burt, John | 1 |
| Cai, Lin | 1 |
| Calcinai, Barbara | 1 |
| Calegari, Marco | 1 |
| Camp, Emma | 1 |
| Capel, Kátia | 1 |
| Casado, Clara | 1 |
| Casareto, Beattriz | 1 |
| Castellan, Giorgio | 1 |
| Castillo, Karl | 1 |
| Chai, Ka Ho | 1 |
| Chang, Taison Ka Tai | 1 |
| Chaudhary, Chhaya | 1 |
| Chavez, Juan | 1 |
| Chen, Tianran | 1 |
| Chen, Xiaoyan | 1 |
| Chen, Zesheng | 1 |
| Choi, Kwang-Sik | 1 |
| Chow, Wing-Kuen | 1 |
| Chung, Jeffrey | 1 |
| Claisius, Ella | 1 |
| Clode, Peta | 1 |
| Cocito, Silvia | 1 |
| Cohen, Anne | 1 |
| Coma, Rafel | 1 |
| Comeau, Steeve | 1 |
| Cook, Katie | 1 |
| Cooper, Antonia | 1 |
| Coppari, Martina | 1 |
| Cornwall, Christopher | 1 |
| Correa, Rogger | 1 |
| Courtney, Travis | 1 |
| Coutinho, Ricardo | 1 |
| Crisci, Caroline | 1 |
| Dafforn, Katherine | 1 |
| Davis, Kay | 1 |
| Davis, Tom | 1 |
| de Bettignies, Thibaut | 1 |
| de Caralt, Sònia | 1 |
| De, Kalyan | 1 |
| DeCarlo, Thomas | 1 |
| Dellaert, Zoe | 1 |
| Denis-Roy, Lara | 1 |
| Di Camillo, Cristina | 1 |
| Diamond, Sandra | 1 |
| Dickerson, Hayden | 1 |
| Dimond, James | 1 |
| Ding, Wei | 1 |
| Diniz, Mário | 1 |
| Dunic, Jillian | 1 |
| Eakin, Mark | 1 |
| Edgar, Graham | 1 |
| Edgar, Robert | 1 |
| Emslie, Michael | 1 |
| Enochs, Ian | 1 |
| Entwistle, Kristina | 1 |
| Escolà, Marta | 1 |
| Faleiro, Filipa | 1 |
| Falter, James | 1 |
| Falter, Jim | 1 |
| Féral, Jean-Pierre | 1 |
| Ferrari, Renara | 1 |
| Ferreira, Lucas Cabral Lage | 1 |
| Figueiredo, Joana | 1 |
| Filho, Fernando | 1 |
| Finnerty, John | 1 |
| Floc'h, Nicolas | 1 |
| Flores, Augusto | 1 |
| Foster, Taryn | 1 |
| Francini, Carlo | 1 |
| Francini-Filho, Ronaldo | 1 |
| Fraser, Nicole | 1 |
| Frieta-Vali?, Maša | 1 |
| Fromont, Jane | 1 |
| Fuchs, Yann | 1 |
| Fujise, Lisa | 1 |
| Fumani, Neda | 1 |
| Gevaert, Francois | 1 |
| Gili, Josep-Maria | 1 |
| Gilliam, David | 1 |
| Giraldo-Ospina, Ana | 1 |
| Gizzi, Francesca | 1 |
| Glassom, David | 1 |
| Gomez-Cabrera, Maria | 1 |
| Gómez-Gras, Daniel | 1 |
| Gong, Sanqiang | 1 |
| González-Pech, Raúl A. | 1 |
| Gori, Andrea | 1 |
| Goyen, Samantha | 1 |
| Guidi, Elena | 1 |
| Gurgel, Anne | 1 |
| Hadjioannou, Louis | 1 |
| Hall-Spencer, Jason | 1 |
| Harborne, Alastair | 1 |
| Harrison, Peter | 1 |
| Harvey, Ben | 1 |
| Hashtroudi, Mehri | 1 |
| Hay, Mark | 1 |
| Heather, Freddie | 1 |
| Heron, Scott | 1 |
| Hicks, Jamie | 1 |
| Higuchi, Tomihiko | 1 |
| Hill, Ross | 1 |
| Hodge, Jessica | 1 |
| Holcomb, Michael | 1 |
| Howe, Steffan | 1 |
| Hsieh, Hernyi | 1 |
| Huang, Hui | 1 |
| Huang, Rongyong | 1 |
| Huang, Zhi | 1 |
| Huges, Terence | 1 |
| Hughes, David | 1 |
| Iguchi, Akira | 1 |
| Ingole, Baban | 1 |
| Ip, Jack Chi-Ho | 1 |
| Jami, Mohammad Javad | 1 |
| Janquin, M-A | 1 |
| Jiang, Leilei | 1 |
| Jimenez, Carlos | 1 |
| Jin, Xuejie | 1 |
| Johnson, Olivia | 1 |
| Johnston, Emma | 1 |
| Johnston, Nicole | 1 |
| Jones, Nicolas | 1 |
| Jones, Tyson | 1 |
| Jordan, Alan | 1 |
| Júnior, Francisco | 1 |
| Kahike, Tim | 1 |
| Kaufman, Les | 1 |
| Kavousi, Javid | 1 |
| Kei, Keith | 1 |
| Keith, Sally | 1 |
| Kendrick, Gary | 1 |
| Kenkel, Carly | 1 |
| Kersting, Diego | 1 |
| Kerwin, Allison | 1 |
| Keshavmurthy, Shashank | 1 |
| Kim, Sun | 1 |
| Kirkland, Amanda | 1 |
| Kitahara, Marcelo | 1 |
| Kitano, Yuko | 1 |
| Klepac, Courtney | 1 |
| Knott, Nathan | 1 |
| Kolodziej, Graham | 1 |
| Komatsu, Kosei | 1 |
| Kon, Koetsu | 1 |
| Kurihara, Haruko | 1 |
| Kuroyama, Mayumi | 1 |
| Kyriacou, Nicole | 1 |
| La Rivière, Marie | 1 |
| La Riviere, Patrick | 1 |
| Lachs, Liam | 1 |
| LaJeunensse, Todd | 1 |
| Larkin, Meryl | 1 |
| Lau, Dickey | 1 |
| Le Nohaïc, Morane | 1 |
| Ledoux, Jean-Baptiste | 1 |
| Legorreta, Renata | 1 |
| Lester, Emily | 1 |
| Leung, Yu Hin | 1 |
| Levy, Oren | 1 |
| Lewis, Brett | 1 |
| Li, Ming | 1 |
| Li, Shu | 1 |
| Liao, Zhuiheng | 1 |
| Liesegang, Mary | 1 |
| Lima, Laís | 1 |
| Ling, Scott | 1 |
| Lipej, Lovrenc | 1 |
| Liu, Gang | 1 |
| Liu, Sheng | 1 |
| Lloyd, Alicia | 1 |
| Longo, Guilherme Ortigara | 1 |
| Lopes, Ana | 1 |
| Lowe, Ryan | 1 |
| Lucas, Caroline | 1 |
| Luo, Yanqiu | 1 |
| Mantas, Torcuato | 1 |
| Manzello, Derek | 1 |
| Marrocco, Teo | 1 |
| Marschal, Christian | 1 |
| Matis, Paloma | 1 |
| Matthews, Jennifer | 1 |
| Matz, Mikhail | 1 |
| Mavri?, Borut | 1 |
| McClanahan, Tim | 1 |
| McCorkle, Daniel | 1 |
| McMahon, Ashly | 1 |
| Mellin, Camille | 1 |
| Meng, Linqing | 1 |
| Menu, Dominique | 1 |
| Mezaki, Takuma | 1 |
| Mies, Miguel | 1 |
| Milazzo, Marco | 1 |
| Mizrahi, Damián | 1 |
| Mo, Shaohua | 1 |
| Mokhtar-Jamaï, Kenza | 1 |
| Montagna, Paolo | 1 |
| Monteiro, Ana | 1 |
| Mooney, Peter | 1 |
| Moreno, Sergio | 1 |
| Moriarty, Tess | 1 |
| Mote, Sambhaji | 1 |
| Muthiga, Nyawira | 1 |
| Nagai, Satoshi | 1 |
| Nakabayashi, Aki | 1 |
| Nakamura, Masako | 1 |
| Nanajkar, Mandar | 1 |
| Negaresta, Hossein | 1 |
| Ng, Ysz Yan | 1 |
| Nitschke, Matthew | 1 |
| Nomura, Keiichi | 1 |
| Nozawa, Yoko | 1 |
| Oakley, Clinton | 1 |
| O'Flaherty, Cliodhna | 1 |
| Oh, Elizabeth | 1 |
| Orejas, Covadonga | 1 |
| Padilla-Gamiño, Jacqueline | 1 |
| Paiva, Sandra | 1 |
| Parkinson, Hogn Everett | 1 |
| Peirano, Andrea | 1 |
| Pimentel, Marta | 1 |
| Pizarro, Oscar | 1 |
| Polinski, Jennifer | 1 |
| Popija?, Aleksandar | 1 |
| Porter, Sean | 1 |
| Prantoni, Selena | 1 |
| Pratchett, Morgan | 1 |
| Puce, stefania | 1 |
| Ralph, Peter | 1 |
| Reavis, Jennifer | 1 |
| Ren, Lijuan | 1 |
| Repolho, Tiago | 1 |
| Reyes, Christopher | 1 |
| Reynaud, Stephanie | 1 |
| Reynaud, Stéphanie | 1 |
| Ribes, Marta | 1 |
| Ricci, Francesco | 1 |
| Rivera, Hanny | 1 |
| Roberson, Loretta | 1 |
| Robert, Keyzers | 1 |
| Rodic, Petra | 1 |
| Rodríguez, Adriana | 1 |
| Roff, George | 1 |
| Rosa, Inês | 1 |
| Rosa, Rui | 1 |
| Rottier, Cecile | 1 |
| Roveta, Camilla | 1 |
| Saeedi, Hanieh | 1 |
| Saleh, Abolfazl | 1 |
| Salguero-Gómez, Roberto | 1 |
| Salih, Anya | 1 |
| Samaroo, Jason | 1 |
| Samiei, Jahangir Vajed | 1 |
| Santos, Isaac | 1 |
| Schleyer, Michael | 1 |
| Schmidt-Roach, Sebastian | 1 |
| Schniedewind, Mikarla | 1 |
| Schutter, Miriam | 1 |
| Seroussi, Yanir | 1 |
| Serra, Ignasi | 1 |
| Sfenthourakis, Spyros | 1 |
| Sharp, Koty | 1 |
| Sharp, Victoria | 1 |
| Shields, Derek | 1 |
| Shields, Joe | 1 |
| Shirvani, Arash | 1 |
| Short, JA | 1 |
| Simões, Marianna | 1 |
| Sims, Carrie | 1 |
| Sink, Kerry | 1 |
| Skirving, William | 1 |
| Smit, Kaylee | 1 |
| Smith, Stephen | 1 |
| Soares, Marcelo | 1 |
| Soler, German | 1 |
| Souza, Fabio | 1 |
| Sproles, Ashley | 1 |
| Stat, Michael | 1 |
| Steinberg, Rosemary | 1 |
| Stewart, FJ | 1 |
| Strudwick, Paige | 1 |
| Stuart-Smith, Jemina | 1 |
| Stuart-Smith, Rick | 1 |
| Sugihara, Kaoru | 1 |
| Sumida, Paulo | 1 |
| Sun, Jin | 1 |
| Suzuki, Yoshimi | 1 |
| Sweatman, Hugh | 1 |
| Tan, Yehui | 1 |
| Taviani, Marco | 1 |
| Taylor, Michael | 1 |
| Teixeira, Carlos | 1 |
| Teshima, Kosuke | 1 |
| Thompson, Philip | 1 |
| Thomson, Damian | 1 |
| Thornhill, Daniel | 1 |
| Tian, Renmao | 1 |
| Tong, Haoya | 1 |
| Torsani, Fabrizio | 1 |
| Tortorelli, Giada | 1 |
| Townsend, Joseph | 1 |
| Tramonte, Carlos | 1 |
| Trubenbach, Katja | 1 |
| Trumble, Isabela | 1 |
| Tuckett, Chenae | 1 |
| Turak, Emre | 1 |
| Turnbull, John | 1 |
| Valisano, Laura | 1 |
| Vargas, Phillip | 1 |
| Viladrich, Núria | 1 |
| Vinagre, Catarina | 1 |
| Voss, Joshua | 1 |
| Wada, Shigeki | 1 |
| Wang, Wenhuan | 1 |
| Wang, Yinghui | 1 |
| Wang, Yonggang | 1 |
| Wernberg, Thomas | 1 |
| Wicks, Laura | 1 |
| Wilkinson, Shaun | 1 |
| Williams, Stefan | 1 |
| Willians, Maureen | 1 |
| Wilson, Shaun | 1 |
| Wong, Tim | 1 |
| Woolsey, Erika | 1 |
| Wu, Qian | 1 |
| Wuitchik, Daniel | 1 |
| Xia, Xiaomin | 1 |
| Xu, Shendong | 1 |
| Yamakita, Takehisa | 1 |
| Yang, Enguang | 1 |
| Yasuda, Nina | 1 |
| Yeung, Chung Wing | 1 |
| Yiu, Sam | 1 |
| Yu, Vriko | 1 |
| Yu, Wanjun | 1 |
| Yuan, Felix | 1 |
| Yuyama, Ikuko | 1 |
| Zhang, Weipang | 1 |
| Zhang, Wenjing | 1 |
| Zhang, Yanjie | 1 |
| Zhou, Guowei | 1 |
| Zimmerman, Richard | 1 |
| Zuberer, Frederic | 1 |
