# Supplementary material for: What is the evidence for the impact of ocean warming on subtropical and temperate corals and coral reefs? A systematic map
Source: Environ Evid. 2024 Nov 21;13:25. doi: 10.1186/s13750-024-00349-y (PMC11580339; doi:10.1186/s13750-024-00349-y)
Supplement: Supplementary file 10 — Additional file 10. [file 13750_2024_349_MOESM10_ESM.docx]

**Additional File 9 Articles with Temperature Raw Data**

**ReadMe**

This is the additional file for the systematic map by Ho et al. (2024): **What is the evidence for the impact of ocean warming on subtropical and temperate corals and coral reefs? A systematic map.** The file describes the list of articles that have open access to the raw temperature data used in their study.

The first column describes the titles of the articles.

The second column describes the last names of the first authors.

The third column describes the years of publications.

The fourth and final column describes the Digital Object Identifiers of the articles (if applicable).

Additional File 9 List of articles with raw temperature data available in their study.

| Title | First Author | Year of Publication | DOI |
| --- | --- | --- | --- |
| A simple temperature-based model predicts the upper latitudinal limit of the temperate coral Astrangia poculata | Dimond | 2013 | 10.1007/s00338-012-0983-z |
| A skeletal Sr/Ca record preserved in Dipsastraea (Favia) speciosa and implications for coral Sr/Ca thermometry in mid-latitude regions | Seo | 2013 | 10.1002/ggge.20195 |
| An approach for assessing ecosystem-based adaptation in coral reefs at relatively high latitudes to climate change and human pressure | Wang | 2020 | 10.1007/s10661-020-08534-5 |
| An urban intertidal reef is dominated by fleshy macroalgae, sediment, and bleaching of a resilient coral (Siderastrea stellata) | Barros | 2021 | 10.1016/j.marpolbul.2021.112967 |
| Application of remotely sensed sea surface temperature for assessment of recurrent coral bleaching (2014â€“2019) impact on a marginal coral ecosystem | De | 2022 | 10.1080/10106049.2021.1886345 |
| Are corals coming to a reef near you? Projected extension of suitable thermal conditions for hard coral communities along the east Australian coast | Davis | 2023 | 10.1111/aec.13327 |
| Branching coral as a macroalgal refuge in a marginal coral reef system | Bennett | 2010 | 10.1007/s00338-010-0594-5 |
| Calcification of the main reef-building coral species on the Pacific coast of southern Mexico | MedellÃ­n-Maldonado | 2016 | 10.7773/cm.v42i3.2650 |
| Characterization of a thermally tolerant Orbicella faveolata reef in Abaco, The Bahamas | Parker | 2020 | 10.1007/s00338-020-01948-0 |
| Climate-induced species range shift and local adaptation strategies in a temperate marine protected area, Ashizuri-Uwakai National Park, Shikoku Island, western Japan | Abe | 2021 | 10.1016/j.ocecoaman.2021.105744 |
| Conserving potential coral reef refuges at high latitudes | Beger | 2014 | 10.1111/ddi.12140 |
| Coral communities of Hong Kong: Long-lived corals in a marginal reef environment | Goodkin | 2011 | 10.3354/meps09019 |
| Coral community dynamics and shallow-water carbonate deposition of the reef-flat around Yongxing Island, the Xisha Islands | JianWei | 2013 | 10.1007/s11430-013-4677-3 |
| Coral cover and rubble cryptofauna abundance and diversity at outplanted reefs in Okinawa, Japan | Biondi | 2020 | 10.7717/peerj.9185 |
| Coral distribution and bleaching vulnerability areas in Southwestern Atlantic under ocean warming | Bleuel | 2021 | 10.1038/s41598-021-92202-2 |
| Coral reef ecosystems under climate change and ocean acidification | Hoegh-Guldberg | 2017 | 10.3389/fmars.2017.00158 |
| Coral reefs at 34 degrees N, Japan: Exploring the end of environmental gradients | Yamano | 2012 | 10.1130/G33293.1 |
| Coral reproduction in a high-latitude, marginal reef environment (Moreton Bay, south-east Queensland, Australia) | Fellegara | 2013 | 10.1080/07924259.2012.752766 |
| Coral responses to ocean warming and acidification: Implications for future distribution of coral reefs in the South China Sea | Yuan | 2019 | 10.1016/j.marpolbul.2018.11.053 |
| Corallivory plays a limited role in the mortality of new coral recruits in Hong Kong marginal coral communities | Tsang | 2018 | 10.1016/j.jembe.2018.03.003 |
| Corals at the edge of environmental limits: A new conceptual framework to re-define marginal and extreme coral communities | Schoepf | 2023 | 10.1016/j.scitotenv.2023.163688 |
| Coupling high-resolution coral bleaching modeling with management practices to identify areas for conservation in a warming climate: Keramashoto National Park (Okinawa Prefecture, Japan) | Abe | 2021 | 10.1016/j.scitotenv.2021.148094 |
| Coverage, Diversity, and Functionality of a High-Latitude Coral Community (Tatsukushi, Shikoku Island, Japan) | Denis | 2013 | 10.1371/journal.pone.0054330 |
| Diazotroph Diversity Associated With Scleractinian Corals and Its Relationships With Environmental Variables in the South China Sea | Liang | 2020 | 10.3389/fphys.2020.00615 |
| Differences in physiological response to increased seawater temperature in nearshore and offshore corals in northern Vietnam | Faxneld | 2011 | 10.1016/j.marenvres.2011.01.007 |
| Differential establishment potential of species predicts a shift in coral assemblage structure across a biogeographic barrier | Keith | 2015 | 10.1111/ecog.01437 |
| Differential Symbiodiniaceae Association With Coral and Coral-Eroding Sponge in a Bleaching Impacted Marginal Coral Reef Environment | Mote | 2021 | 10.3389/fmars.2021.666825 |
| Diminishing potential for tropical reefs to function as coral diversity strongholds under climate change conditions | Adam | 2021 | 10.1111/ddi.13400 |
| Dominance of the scleractinian coral Alveopora japonica in the barren subtidal hard bottom of high-latitude Jeju Island off the south coast of Korea assessed by highresolution underwater images | Lee | 2022 | 10.1371/journal.pone.0275244 |
| Environmental implications of skeletal micro-density and porosity variation in two scleractinian corals | Caroselli | 2011 | 10.1016/j.zool.2011.04.003 |
| Environmental variation and how its spatial structure influences the cross-shelf distribution of high-latitude coral communities in South Africa | Porter | 2019 | 10.3390/d11040057 |
| Fluctuations in coral health of four common inshore reef corals in response to seasonal and anthropogenic changes in water quality | Browne | 2015 | 10.1016/j.marenvres.2015.02.002 |
| Future habitat suitability for coral reef ecosystems under global warming and ocean acidification | Couce | 2013 | 10.1111/gcb.12335 |
| Genetic structure of Turbinaria peltata in the northern South China Sea suggest insufficient genetic adaptability of relatively high-latitude scleractinian corals to environment stress | Wu | 2021 | 10.1016/j.scitotenv.2021.145775 |
| Genomic models predict successful coral adaptation if future ocean warming rates are reduced | Bay | 2017 | 10.1126/sciadv.1701413 |
| Global biogeography of coral recruitment: tropical decline and subtropical increase | Price | 2019 | 10.3354/meps12980 |
| Growth form-dependent response to physical disturbance and thermal stress in Acropora corals | Muko | 2013 | 10.1007/s00338-012-0967-z |
| Holocene reef-growth dynamics on Kodakara Island (29Â°N, 129Â°E) in the Northwest Pacific | Hamanaka | 2015 | 10.1016/j.geomorph.2015.04.011 |
| How does a widespread reef coral maintain a population in an isolated environment? | Precoda | 2018 | 10.3354/meps12537 |
| Ideas and perspectives: Southwestern tropical Atlantic coral growth response to atmospheric circulation changes induced by ozone depletion in Antarctica | Evangelista | 2016 | 10.5194/bg-13-2379-2016 |
| In the right place at the right time: representativeness of corals within marine protected areas under warming scenarios in Brazil | Albuquerque | 2023 | 10.1016/j.ocecoaman.2022.106469 |
| Instability in a marginal coral reef: The shift from natural variability to a human-dominated seascape | Lybolt | 2011 | 10.1890/090176 |
| Invasive sun corals and warming pose independent threats to the brain coral Mussismilia hispida in the Southwestern Atlantic | Barbosa | 2019 | 10.3354/meps13110 |
| Large-amplitude internal waves benefit corals during thermal stress | Wall | 2015 | 10.1098/rspb.2014.0650 |
| Large-amplitude internal waves sustain coral health during thermal stress | Schmidt | 2016 | 10.1007/s00338-016-1450-z |
| Latitudinal difference in the species richness of photosymbiotic ascidians along the east coast of Taiwan | Hirose | 2020 | 10.6620/ZS.2020.59-19 |
| Limited scope for latitudinal extension of reef corals | Muir | 2015 | 10.1126/science.aab4122 |
| Living on the edge: environmental variability of a shallow late Holocene cold-water coral mound | Raddatz | 2022 | 10.1007/s00338-022-02249-4 |
| Locality Effect of Coral-Associated Bacterial Community in the Kuroshio Current From Taiwan to Japan | Yang | 2020 | 10.3389/fevo.2020.569107 |
| Long-term spatial variations in turbidity and temperature provide new insights into coral-algal states on extreme/marginal reefs | Cartwright | 2023 | 10.1007/s00338-023-02393-5 |
| Low Symbiodiniaceae diversity in a turbid marginal reef environment | Smith | 2020 | 10.1007/s00338-020-01956-0 |
| Low symbiont diversity as a potential adaptive strategy in a marginal non-reefal environment: a case study of corals in Hong Kong | Ng | 2016 | 10.1007/s00338-016-1458-4 |
| Macrobioerosion in Porites corals in subtropical northern South China Sea: a limiting factor for high-latitude reef framework development | Chen | 2013 | 10.1007/s00338-012-0946-4 |
| Management of scleractinian coral assemblages in temperate non-reefal areas: insights from a long-term monitoring study in Kushimoto, Japan (33Â°N) | Nakamura | 2021 | 10.1007/s00227-021-03948-2 |
| Marine heatwave events near Weizhou Island, Beibu Gulf in 2020 and their possible relations to coral bleaching | Feng | 2022 | 10.1016/j.scitotenv.2022.153414 |
| Mesophotic corals on the subtropical shelves of Lord Howe Island and Balls Pyramid, south-western Pacific Ocean | Linklater | 2019 | 10.1071/MF18151 |
| Metabolic and metatranscriptional characteristics of corals bleaching induced by the most severe marine heatwaves in the South China Sea | Sun | 2023 | 10.1016/j.scitotenv.2022.160019 |
| Mid-latitude coralgal bioconstruction and endolithic microbialites: environmental significance during Quaternary climate variations | Bernasconi | 2015 | 10.1007/s10347-015-0449-1 |
| Mortality, growth and regeneration following fragmentation of reef-forming corals under thermal stress | Dias | 2018 | 10.1016/j.seares.2018.08.008 |
| Natural heterotrophic feeding by a temperate octocoral with symbiotic zooxanthellae: a contribution to understanding the mechanisms of die-off events | Coma | 2015 | 10.1007/s00338-015-1281-3 |
| New insights from coral growth band studies in an era of rapid environmental change | Lough | 2011 | 10.1016/j.earscirev.2011.07.001 |
| No evidence for tropicalization of coral assemblages in a subtropical climate change hot spot | Mizerek | 2021 | 10.1007/s00338-021-02167-x |
| Occasional loss of fecundity in peripheral coral populations | Suzuki | 2021 | 10.1111/1440-1703.12177 |
| Opposite latitudinal gradients in projected ocean acidification and bleaching impacts on coral reefs | Van Hooidonk | 2014 | 10.1111/gcb.12394 |
| Opposite latitudinal gradients in projected ocean acidification and bleaching impacts on coral reefs | Van Hooidonk | 2014 | 10.1111/gcb.12394 |
| Patterns of scleractinian coral recruitment at Lord Howe Island, an isolated subtropical reef off eastern Australia | Cameron | 2016 | 10.1007/s00338-016-1414-3 |
| Physiological responses and adjustments of corals to strong seasonal temperature variations (20â€“28Â°C) | Sawall | 2022 | 10.1242/jeb.244196 |
| Poleward extension of reefs | Woodroffe | 2011 | 10.1007/978-90-481-2639-2_125 |
| Population genetic structure of a broadcast-spawning coral across a tropicalâ€“temperate transition zone reveals regional differentiation and high-latitude reef isolation | Evans | 2021 | 10.1111/jbi.14280 |
| Potential changes in the distribution of the genus Pocillopora (Anthozoa: Scleractinia) in the Eastern Tropical Pacific under climate change scenarios | Stranges | 2019 | 10.22201/ib.20078706e.2019.90.2696 |
| Ranking 67 Florida Reefs for Survival of Acropora cervicornis Outplants | Banister | 2021 | 10.3389/fmars.2021.672574 |
| Recruitment and mortality of the temperate coral Cladocora caespitosa: implications for the recovery of endangered populations | Kersting | 2014 | 10.1007/s00338-014-1144-3 |
| Reefs of last resort: Locating and assessing thermal refugia in the wider Caribbean | Chollett | 2013 | 10.1016/j.biocon.2013.08.010 |
| Regional coral growth responses to seawater warming in the South China Sea | Yan | 2019 | 10.1016/j.scitotenv.2019.03.135 |
| Regional genetic differentiation among northern high-latitude island populations of a broadcast-spawning coral | Nakajima | 2012 | 10.1007/s00338-012-0932-x |
| Restricted gene flow and local adaptation highlight the vulnerability of high-latitude reefs to rapid environmental change | Thomas | 2017 | 10.1111/gcb.13639 |
| Sensitivity of a cold-water coral reef to interannual variability in regional oceanography | Kazanidis | 2021 | 10.1111/ddi.13363 |
| Spatial and Intergeneric Variation in Physiological Indicators of Corals in the South China Sea: Insights Into Their Current State and Their Adaptability to Environmental Stress | Qin | 2019 | 10.1029/2018JC014648 |
| Spatial and Temporal Patterns of Eastern Australia Subtropical Coral Communities | Dalton | 2013 | 10.1371/journal.pone.0075873 |
| Species-specific declines in the linear extension of branching corals at a subtropical reef, Lord Howe Island | Anderson | 2015 | 10.1007/s00338-014-1251-1 |
| Status of Marine Biodiversity of the China Seas | Liu | 2013 | 10.1371/journal.pone.0050719 |
| Survival of high latitude fringing corals in extreme temperatures: Red sea meteorology | Moustafa | 2015 | 10.1016/j.seares.2014.01.012 |
| Sustained mass coral bleaching (2016-2017) in Brazilian turbid-zone reefs: taxonomic, cross-shelf and habitat-related trends | Teixeira | 2019 | 10.1007/s00338-019-01789-6 |
| Symbiodinium clade C generality among common scleractinian corals in subtropical Hong Kong | Wong | 2016 | 10.1016/j.rsma.2016.02.005 |
| Temperature control on high-resolution SIMS oxygen isotopic compositions in Porites coral skeletons | Zou | 2021 | 10.1016/j.sesci.2021.02.002 |
| The contribution of stress-tolerant endosymbiotic dinoflagellate Durusdinium to Pocillopora acuta survival in a highly urbanized reef system | Poquita-Du | 2020 | 10.1007/s00338-020-01902-0 |
| The impacts of flooding on the high-latitude, terrigenoclastic influenced coral reefs of Hervey Bay, Queensland, Australia | Butler | 2013 | 10.1007/s00338-013-1064-7 |
| The Long and Winding Road of Coral Reef Recovery in the Anthropocene: A Case Study from Puerto Rico | HernÃ¡ndez-Delgado | 2022 | 10.3390/d14100804 |
| The Origin of the Subtropical Coral Alveopora japonica (Scleractinia: Acroporidae) in High-Latitude Environments | Kang | 2020 | 10.3389/fevo.2020.00012 |
| The projected degradation of subtropical coral assemblages by recurrent thermal stress | Cant | 2021 | 10.1111/1365-2656.13340 |
| The Reef Coral Coscinaraea marshae Is Not a High-Latitude Endemic | Hoeksema | 2021 | 10.3390/d13120681 |
| Tracking widespread climate-driven change on temperate and tropical reefs | Stuart-Smith | 2022 | 10.1016/j.cub.2022.07.067 |
| Transient amplification enhances the persistence of tropicalising coral assemblages in marginal high-latitude environments | Cant | 2022 | 10.1111/ecog.06156 |
| Tropicalisation of temperate reefs: Implications for ecosystem functions and management actions | Verges | 2019 | 10.1111/1365-2435.13310 |
| Unraveling Moreton Bay reef history: An urban high-latitude setting for coral development | Hammerman | 2022 | 10.3389/fevo.2022.884850 |
| Variations in the coral community at the high-latitude Bailong Peninsula, northern South China Sea | Wang | 2023 | 10.1007/s11356-022-21881-9 |
| Warmer more acidic conditions cause decreased productivity and calcification in subtropical coral reef sediment-dwelling calcifiers | Sinutok | 2011 | 10.4319/lo.2011.56.4.1200 |
| Winter Quiescence, Growth Rate, and the Release from Competition in the Temperate Scleractinian Coral Astrangia poculata (Ellis | Grace | 2017 | 10.1656/045.024.s715 |
