# Supplementary material for: What is the evidence for the impact of ocean warming on subtropical and temperate corals and coral reefs? A systematic map
Source: Environ Evid. 2024 Nov 21;13:25. doi: 10.1186/s13750-024-00349-y (PMC11580339; doi:10.1186/s13750-024-00349-y)
Supplement: Supplementary file 2 — Additional file 2. [file 13750_2024_349_MOESM2_ESM.pdf]

ROSES for Systematic Map Reports. Version 1.0

November 2017
Neal R Haddaway, Biljana Macura, Paul Whaley, and Andrew Pullin

Column descriptions

Section/sub-section - The sections or sub-sections listed below correspond to sections in CEE systematic review and map protocols and final reports: please see the relevant text under these subheadings for information within a specific review.
Topic - the section or sub-section is further broken down for the purposes of clarity.
Description/Further explanation - Details of what is needed in each section or sub-section are provided, along with practical advice and links to relevant guidance.
Checklist/Meta-data - Checklist items MUST be completed. Meta-data items correspond to information that should be reported as data or short descriptions that will be used to populate the Summary Record and Flow Diagram. Any meta-data items where stages were not performed (e.g. grey literature searching), this should be stated (e.g. 'Not performed').
Author response - authors should select or write a response for meta-data, or select 'yes' or 'no' for checklist items. Further details may be provided in Comments (see below).
Comments - authors may supplement their response with a brief comment.

Please cite as: Haddaway NR, Macura B, Whaley P, and Pullin AS. 2017. ROSES for Systematic Map Reports. Version 1.0. DOI: 10.6084/m9.figshare.5897299.

Item	Section/sub-section	Topic	Description	Further explanation	Checklist/meta-data	Author response	Comments
1	Title	Title	The title must indicate that it is a systematic map, and should indicate if it is an update/amendment: e.g. "...A systematic map update."	The title should normally be the same or very similar to the review question.	Meta-data	What is the evidence for the impact of ocean warming on subtropical and temperate corals and coral reefs? A systematic map.	
2	Type of review	Type of review	Select one of the following types of review: systematic map, systematic map update, systematic map amendment	See CEE Guidance on systematic mapping [1], and on amendments and updates [2]	Meta-data	systematic map	
3	Authors' contacts	Authors' contacts	The full names, institutional addresses and email addresses for all authors must be provided.		Checklist	Yes	
4	Abstract	Structured summary	The abstract of the manuscript must not exceed 500 words and must be structured into separate sections: Background, the context and purpose of the review, including the review question; Methods, how the review was performed (specifically mention search strategy, inclusion criteria, critical appraisal (optional), meta-data extraction and coding, and narrative synthesis); Results, the main findings, including results of search and assessment of evidence base; Conclusions, brief summary and potential implications for policy/management and research.		Checklist	Yes	
5	Background	Background	Describe the rationale for the review in the context of what is already known. Reviews must indicate why this study was necessary and what it aims to contribute to the field.	A theory of change and/or conceptual model should be presented that links the intervention or exposure to the outcome.	Checklist	Yes	
6	Stakeholder engagement	Stakeholder engagement	The actual role of stakeholders throughout the review process (e.g. in the formulation of the question) must be described and explained (using a broad definition of 'stakeholder', including e.g. researchers, funders and other decision-makers; see [3])		Checklist	No	No Stakeholder engagement
7	Objective of the review	Objective	Describe the primary question and secondary questions (when applicable).	The primary question is the main question of the review. The secondary questions are usually linked to sources of heterogeneity (effect modifiers).	Checklist	Yes	
8		Definition of the question components	Provide reference to the question key elements, e.g. population(s), intervention(s)/exposure(s), comparator(s), and outcome(s).	For other question types see [4,5]	Meta-data	Population: Subtropical and Temperate Coral Systems and Coral Reefs Exposure: Ocean Warming Comparison: Time-trends, including before-after studies Outcome: Mortality and bleaching, changes in coral habitat, acclimatisation measured by population growth.	
9	Methods	Protocol	Provide citation, DOI or open-access link to published protocol.	The protocol should be peer-reviewed and publicly available online (open access).	Meta-data	10.57808/proceed.2023.9	
10		Deviations from protocol	Describe any ways in which the final methods of the review deviate from those set out in the protocol along with a justification.		Checklist	Yes	
11	Searches	Search strategy	Detail the search strategy used, including: database names accessed, dates of searching, institutional subscriptions (or date ranges subscribed for each database), search options (e.g. 'topic words' or 'full text' search facility), efforts to source grey literature, other sources of evidence (e.g. hand searching, calls for evidence/submission of evidence by stakeholders).		Checklist	Yes	
12		Search string	Provide Boolean-style full search string and state the platform for which the string is formatted (e.g. Web of Science format)		Meta-data	Scopus: TITLE-ABS-KEY ((coral* OR "coral reef*")) AND TITLE-ABS-KEY (("ocean warming" OR "marine heatwave*" OR "marine heat wave*" OR mhw* OR "degree heating week*" OR dhw* OR "heat stress*" OR stress OR tropicali?ation OR temperature* OR "climate change")) AND TITLEABS- KEY ((mortal* OR surviv* OR health* OR diseas* OR grow* OR reprodu* OR cover* OR tropicali?* OR shift* OR habitat* OR increase* OR declin* OR decreas* OR impact* OR threat* OR bleach* OR acclimati?* OR respons*)) AND TITLE-ABS-KEY ((marginal OR "high latitude" OR temperate OR subtropic* OR extratropic*)) Followed by a removal of articles published prior to 2010. Web of Science: (All Fields) ((coral* OR "coral reef*")) AND (All Fields) (("ocean warming" OR "marine heatwave*" OR "marine heat wave*" OR mhw* OR "degree heating week*" OR dhw* OR "heat stress*" OR stress OR tropicali?ation OR temperature* OR "climate change")) AND (All Fields) ((mortal* OR surviv* OR health* OR diseas* OR grow* OR reprodu* OR cover* OR tropicali?* OR shift* OR habitat* OR increase* OR declin* OR decreas* OR impact* OR threat* OR bleach* OR acclimati?* OR respons*)) AND (All Fields) ((marginal OR "high latitude" OR temperate OR subtropic* OR extratropic*)) Followed by removal of articles published prior to 2010.	
13		Languages - bibliographic databases	List languages used in bibliographic database searches		Meta-data	English	
14		Languages – grey literature	List languages used in organisational website searches and web-based search engines		Meta-data	English	
15		Bibliographic databases	Provide the number of bibliographic databases searched		Meta-data	2	
16		Web-based search engines	Provide the number of web-based search engines searched		Meta-data	1	
17		Organisational websites	Provide the number of organisational websites searched		Meta-data	0	
18		Estimating comprehensiveness of the search	Describe the process by which the comprehensiveness of the search strategy was assessed (i.e. list of benchmark articles)		Checklist	Yes	
19		Search update	Describe any update to searches undertaken during the conduct of the review	Compulsory (if update performed). A search update is good practice if original searches were performed more than two years prior to review completion.	Checklist	Yes	
20	Article screening and study inclusion criteria	Screening strategy	Describe the methodology for screening articles/studies for relevance. Methods for consistency of screening decisions (at title, abstract, and full texts levels) checking must be described.		Checklist	Yes	
21		Inclusion criteria	Describe the inclusion criteria used to assess relevance of identified articles/studies. These must be broken down into the question key elements (e.g. relevant subject(s), intervention(s)/exposure(s), comparator(s), outcome(s), study design(s)) and any other restrictions (e.g. date ranges or languages).		Checklist	Yes	
22	Critical appraisal	Critical appraisal strategy	Describe here the method used for critical appraisal of study validity (including assessment of individual studies and the evidence base as a whole). Describe how repeatability of critical appraisal of study validity was tested.	Optional	Checklist	No	No critical appraisal
23		Critical appraisal used in synthesis	Describe how the information from critical appraisal was used in synthesis.	Compulsory if critical appraisal performed	Checklist	No	No critical appraisal
24	Meta-data extraction and coding strategy	Meta-data extraction and coding strategy	Describe the method for meta-data extraction and coding for studies, providing lists of variables that will be extracted as meta-data and those that will be coded. Describe how repeatability of meta-data/data extraction and coding was tested.		Checklist	Yes	
25		Approaches to missing data	Describe any process for obtaining and confirming missing or unclear information or data from authors.		Checklist	No	No missing full text
26	Data synthesis and presentation	Narrative synthesis strategy	Describe methods used for narratively synthesising the evidence base in the form of descriptive statistics, tables (including SM database) and figures.		Checklist	Yes	
27		Knowledge gap and cluster identification strategy	Describe the methods used to identify and/or prioritise key knowledge gaps (unrepresented or underrepresented subtopics that warrant further primary research) and knowledge clusters (well-represented subtopics that are amenable to full synthesis via systematic review).		Checklist	Yes	
28		Demonstrating procedural independence	Describe the role of systematic reviewers (who have also authored articles to be considered within the review) in decisions regarding inclusion or critical appraisal of their own work.	Reviewers who have authored articles to be considered within the review should be prevented from unduly influencing inclusion decisions, for example by delegating tasks appropriately.	Checklist	Yes	
29	Results (review findings)	Description of review process	Describe the review process including the volume of evidence identified from all sources and retained through each stage of the review. Must also display the number of articles/studies included at all stages of the review in a flow diagram, including the number of articles/studies excluded at each stage.		Checklist	Yes	
30		Number of search results	Provide the number of search results from bibliographic databases (including updates if conducted) prior to duplicate removal.	This number should not include web-based search engine or organisational website searches: this will help assessment of the efficiency of the primary search string.	Meta-data	1919	
31		Number of search results after duplicate removal	Provide the total number of search results from bibliographic database searches following duplicate removal.	This number should not include web-based search engine or organisational website searches: this will help assessment of the efficiency of the primary search string.	Meta-data	90	
32		Full text screening excludes	Additional file containing list of and reasons for full text exclusions.		Checklist	Yes	
33		Title screening results	Provide the number of articles retained following title screening.	Optional if screening titles and abstracts together	Meta-data	NA	
34		Abstract screening results	Provide the number of articles retained following abstract screening.	Optional if screening titles and abstracts together	Meta-data	NA	
35		Title and abstract screening results	Provide the number of articles retained following title and abstract screening.	Optional if screening titles and abstracts separately	Meta-data	197	
36		Retrieval results	Provide the number of articles retrieved at full text.		Meta-data	197	
37		Unobtainable articles	Additional file containing list of unobtainable articles.		Checklist	Yes	
38		Full text screening results	Provide the number of articles retained following full text screening.		Meta-data	90	
39		Consistency checking: screening	Results of consistency checking at all stages (screening, meta-data extraction and coding, critical appraisal) must be provided. Provide the number of titles, abstracts and full texts screened and checked for consistency by two or more reviewers as a fraction of the total (e.g. Title: 2000/20000; Abstract: 500/5000: Full text: 10/100).		Checklist	Yes	
40		Narrative synthesis	Describe the body of evidence identified using figures and tables, avoiding vote-counting (tallying of studies based on results; direction or significance). Each must be presented with descriptive information (meta-data). Describe the validity of individual studies and the evidence base as a whole (if critical appraisal conducted).		Checklist	Yes	
41		Systematic map database	Additional file containing meta-data and coding for included studies.		Checklist	Yes	
42		Limitations of the review	Discuss possible limitations in the methods used.		Checklist	Yes	
43		Limitations of the evidence base	Discuss possible limitations in the evidence base.		Checklist	Yes	
44	Conclusions	Knowledge gaps and clusters	Describe knowledge gaps (unrepresented or underrepresented subtopics that warrant further primary research) and knowledge clusters (well-represented subtopics that are amenable to full synthesis via systematic review)		Checklist	Yes	
45		Implications for policy/management	Summarise the state of the evidence base and discuss the way in which the identified evidence may inform policy/practice decision making in relation to the review/map question.	Reviews must not include practical environmental management recommendations or advocacy.	Checklist	Yes	
46		Implications for research	Discuss the way in which the identified evidence may inform research including options for increasing the reliability of study design that could improve future research.	In this section some advocacy for future research on the reviewed topic is permissible provided it is clearly justified by the review outcome/critical appraisal of study validity.	Checklist	Yes	
47	Declarations	Competing interests	Describe of any financial or non-financial competing interests that the review authors may have.		Checklist	Yes	

References

[1] James, K.L., Randall, N.P. and Haddaway, N.R., 2016. A methodology for systematic mapping in environmental sciences. Environmental Evidence, 5(1), p.7.
[2] Bayliss, H.R., Haddaway, N.R., Eales, J., Frampton, G.K. and James, K.L., 2016. Updating and amending systematic reviews and systematic maps in environmental management. Environmental Evidence, 5(1), p.20.
[3] Haddaway, N.R., Kohl, C., da Silva, N.R., Schiemann, J., Spök, A., Stewart, R., Sweet, J.B. and Wilhelm, R., 2017. A framework for stakeholder engagement during systematic reviews and maps in environmental management. *Environmental Evidence*, 6 (1), p.11.
[4] Collaboration for Environmental Evidence. 2018. Guidelines and Standards for Evidence synthesis in Environmental Management. Version 5.0. www.environmentalevidence.org/information-for-authors.
[5] Leeds Institute of Health Sciences. https://medhealth.leeds.ac.uk/info/639/information_specialists/1500/search_concept_tools. Accessed 12/11/2017.
